# Supplementary figures and images for: MARK2 regulates Golgi apparatus reorientation by phosphorylation of CAMSAP2 in directional cell migratio
Source: eLife. 2025 May 7;14:RP105977. doi: 10.7554/eLife.105977 (PMC12058119; doi:10.7554/eLife.105977)

Supplementary figure1B

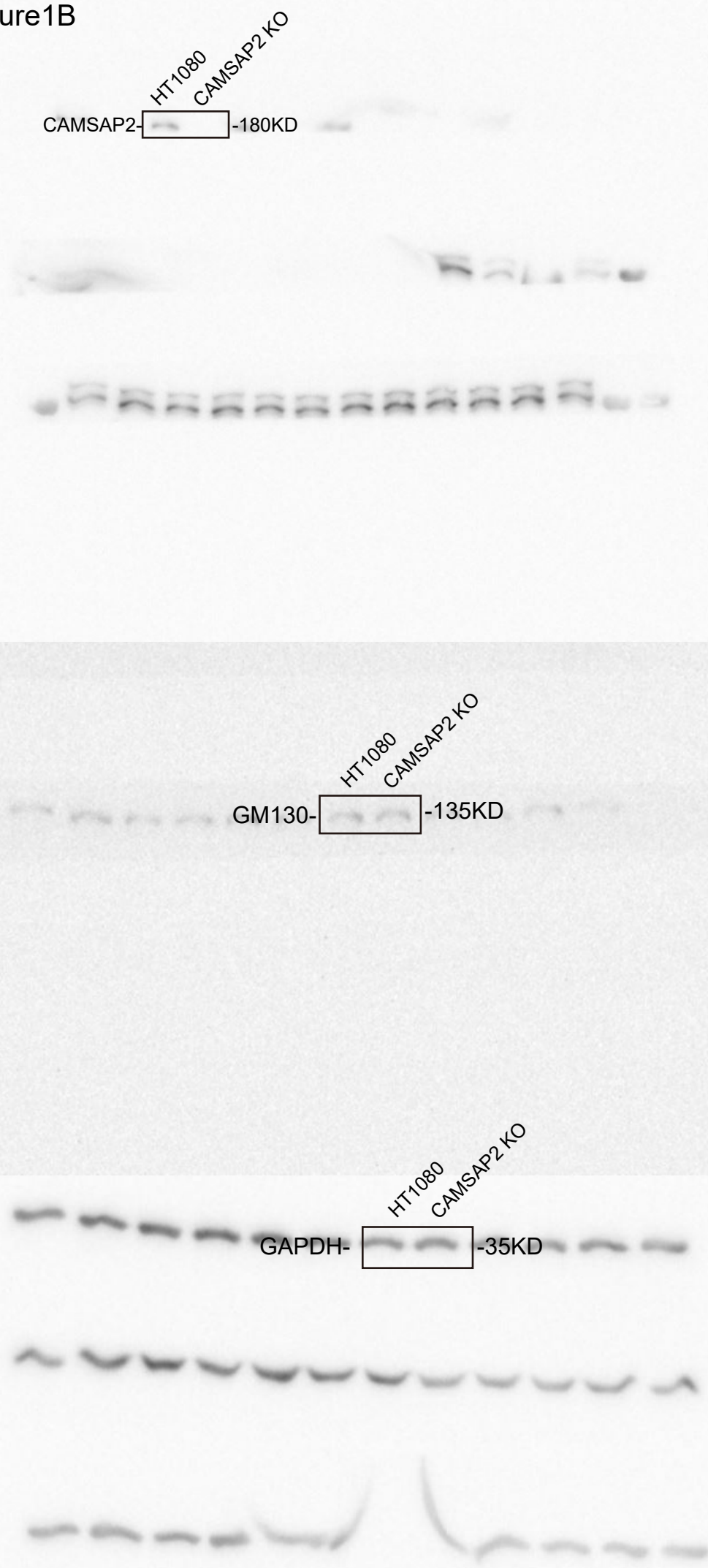

Supplement: Figure 1—figure supplement 1—source data 1. [file elife-105977-fig1-figsupp1-data1.zip › Figure 1-figure supplement 1 source data 1/Supplementary figure1B-source data 1-PDF/Supplementary figure1B-source data1 PDF.pdf]

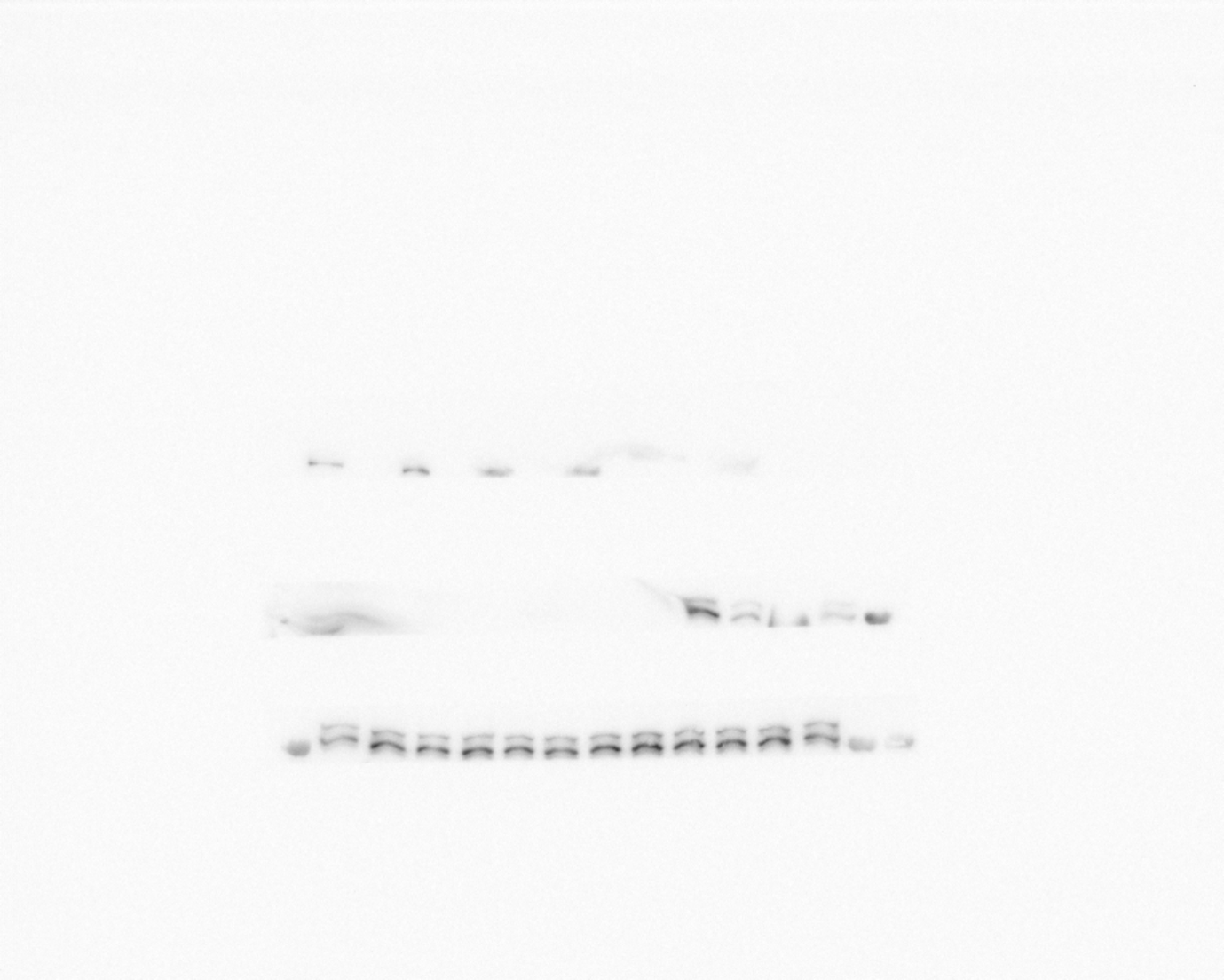

Supplement: Figure 1—figure supplement 1—source data 2. [file elife-105977-fig1-figsupp1-data2.zip › Figure 1-figure supplement 1 source data 2/Supplementary figure1B-source data 2/A-CAMSAP2-Cam20230924_124509_opt_14.TIF]

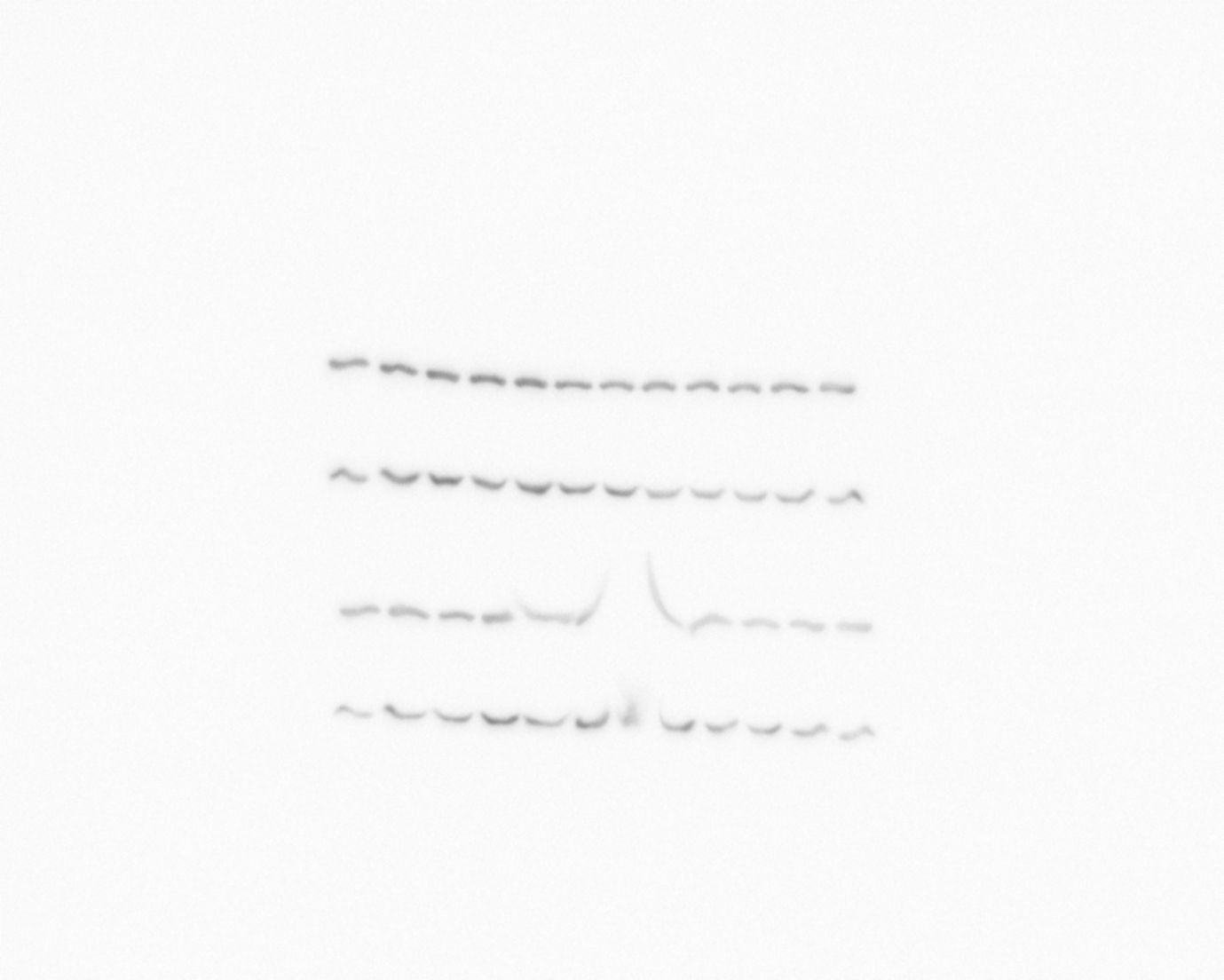

Supplement: Figure 1—figure supplement 1—source data 2. [file elife-105977-fig1-figsupp1-data2.zip › Figure 1-figure supplement 1 source data 2/Supplementary figure1B-source data 2/A-GAPDH-Cam20230924_124203_opt_12.TIF]

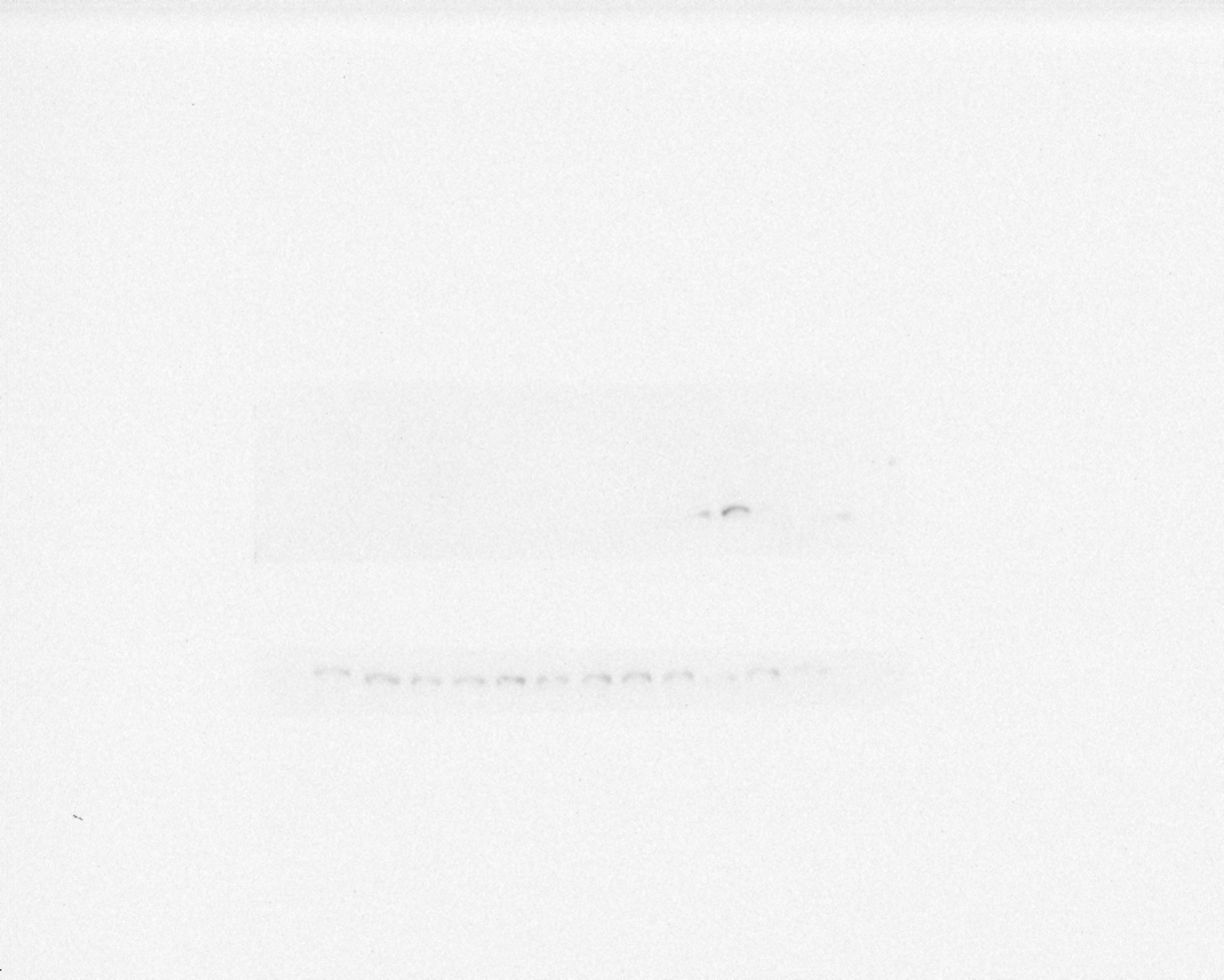

Supplement: Figure 1—figure supplement 1—source data 2. [file elife-105977-fig1-figsupp1-data2.zip › Figure 1-figure supplement 1 source data 2/Supplementary figure1B-source data 2/A-GM130-Cam20230924_125154_opt_27.TIF]

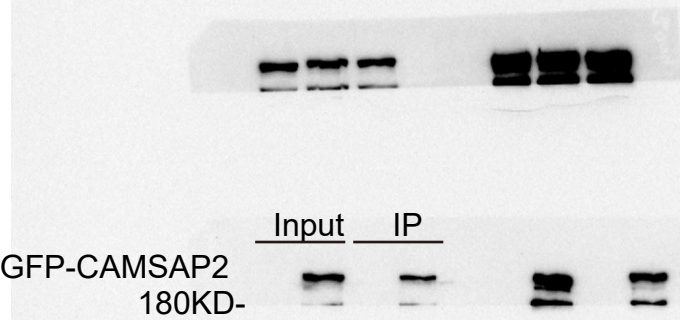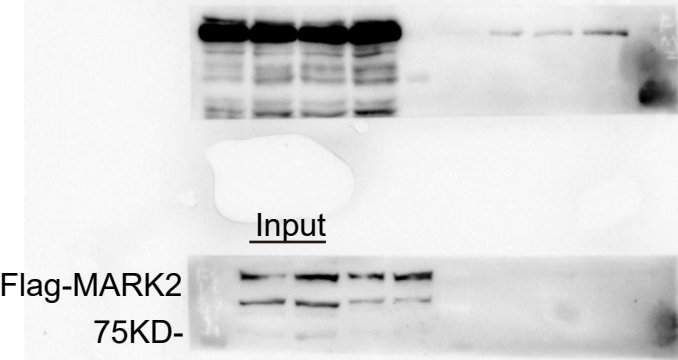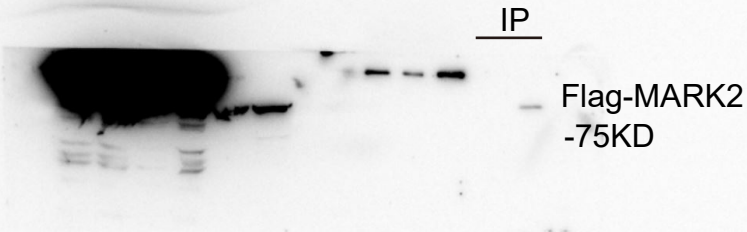

Supplement: Figure 2—source data 1. [file elife-105977-fig2-data1.zip › Figure 2 - Source data 1/Figure2C.pdf]

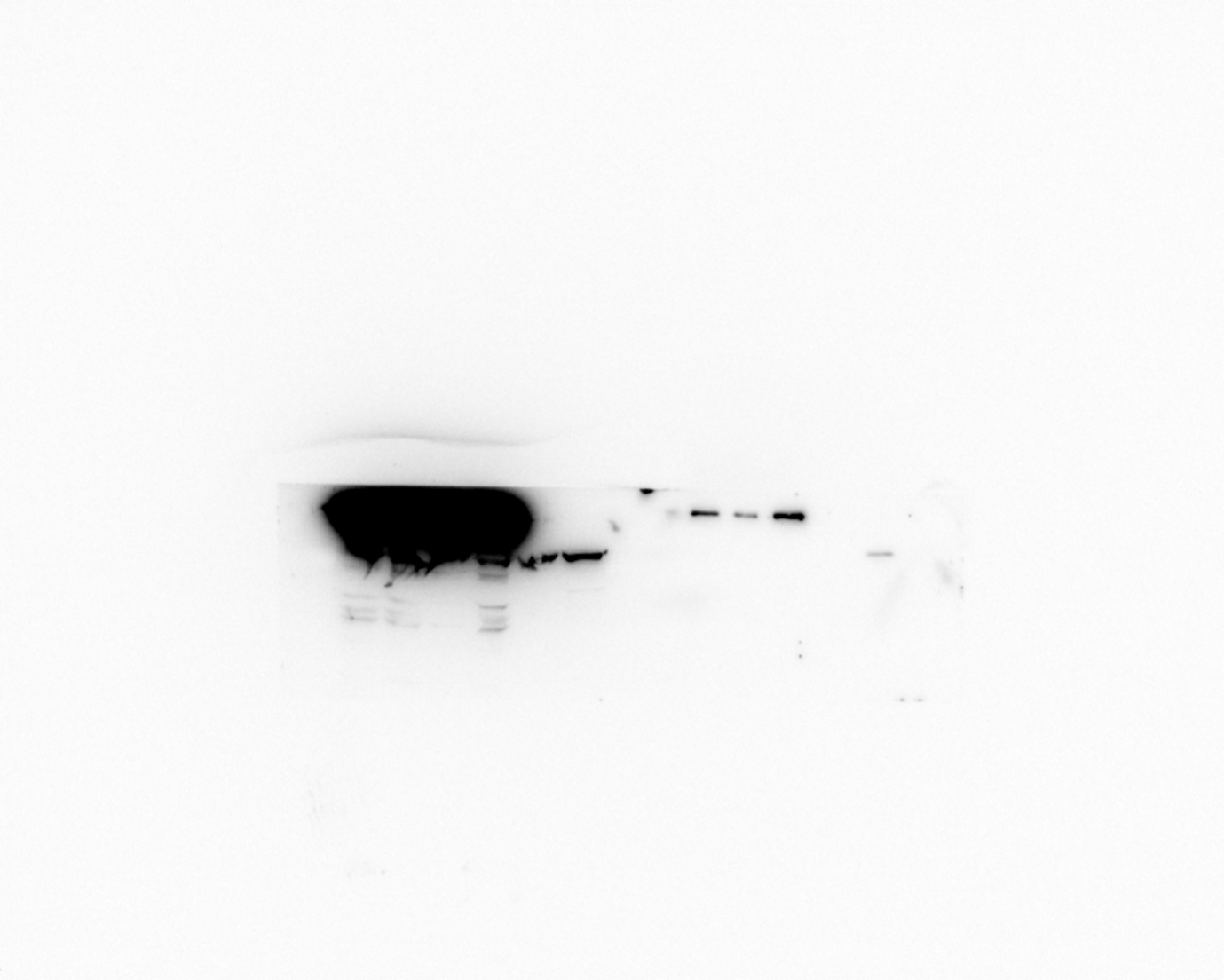

Supplement: Figure 2—source data 2. [file elife-105977-fig2-data2.zip › Figure 2 - Source data 2/A-Figure2C-IP-FLAG-MARK2Cam20231215_122336_opt-_11.TIF]

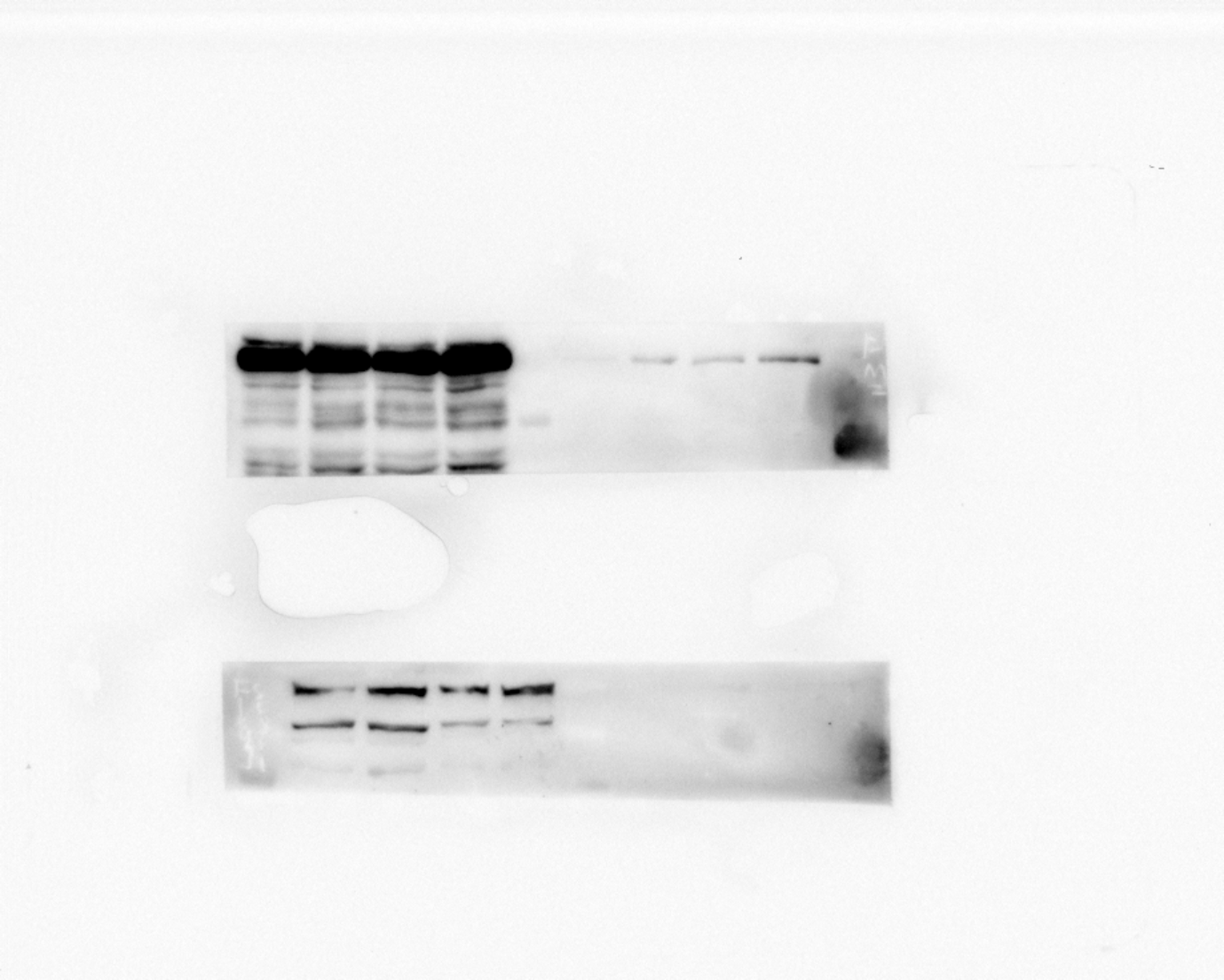

Supplement: Figure 2—source data 2. [file elife-105977-fig2-data2.zip › Figure 2 - Source data 2/A-Figure2C-Input-MARK2-Cam20231216_130232_opt_16.TIF]

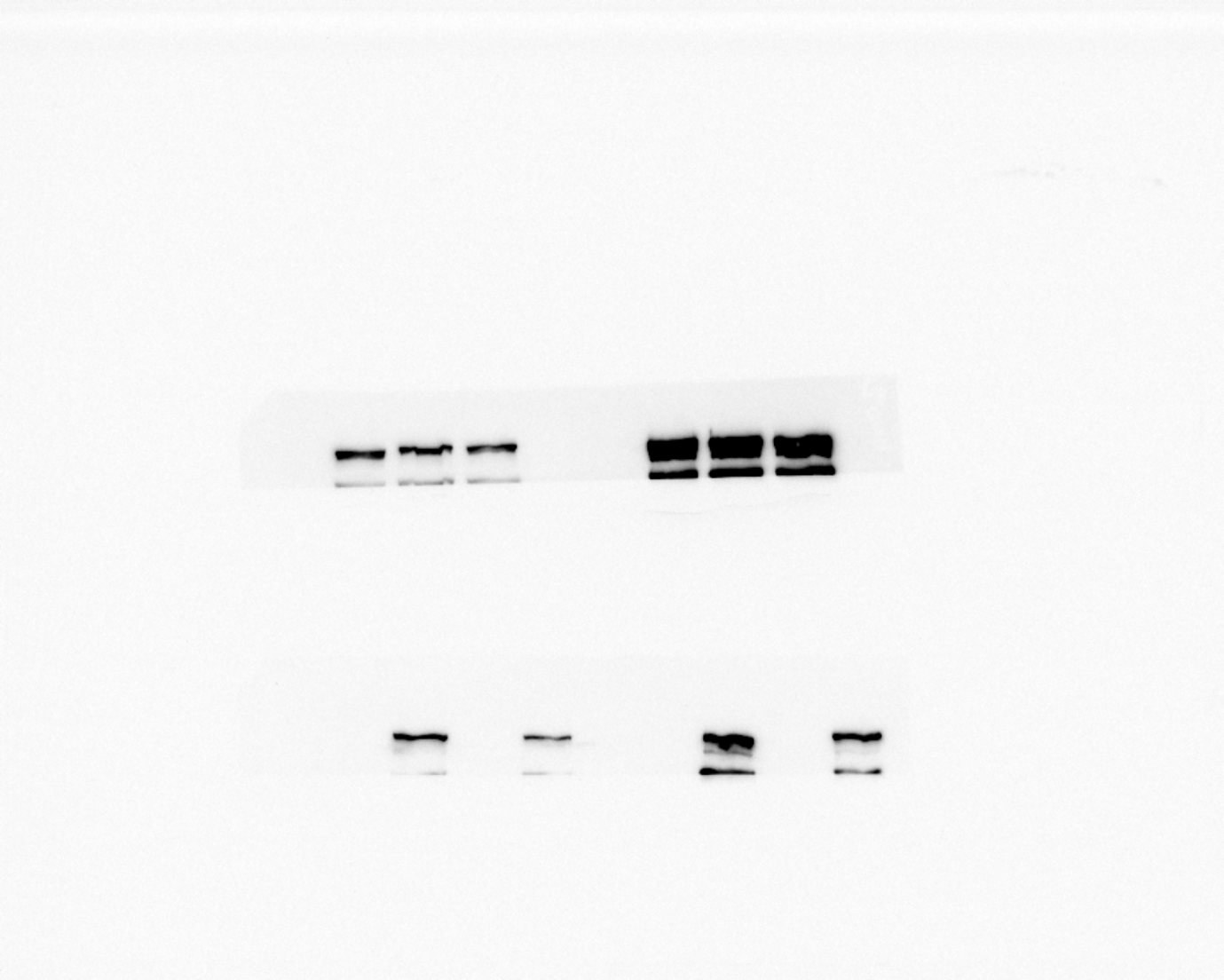

Supplement: Figure 2—source data 2. [file elife-105977-fig2-data2.zip › Figure 2 - Source data 2/A-Figure2C-input-IP-GFP-CAMSAP2-Cam20231216_130811_opt_15.TIF]

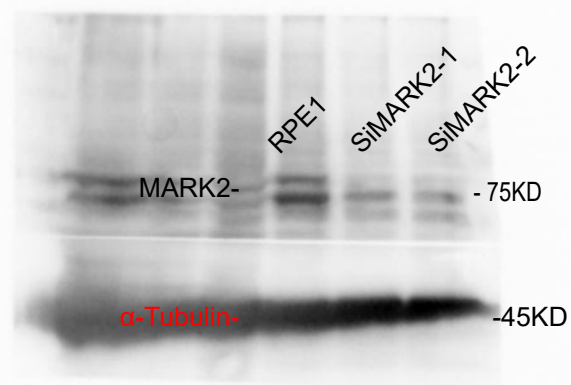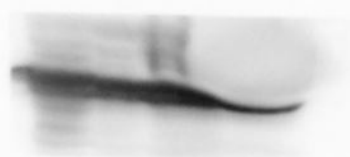

Supplement: Figure 2—figure supplement 1—source data 1. [file elife-105977-fig2-figsupp1-data1.zip › Figure 2-figure supplement 1 source data 1/Figure 2-figure supplement 1F source data .pdf]

Supplement Figure3B

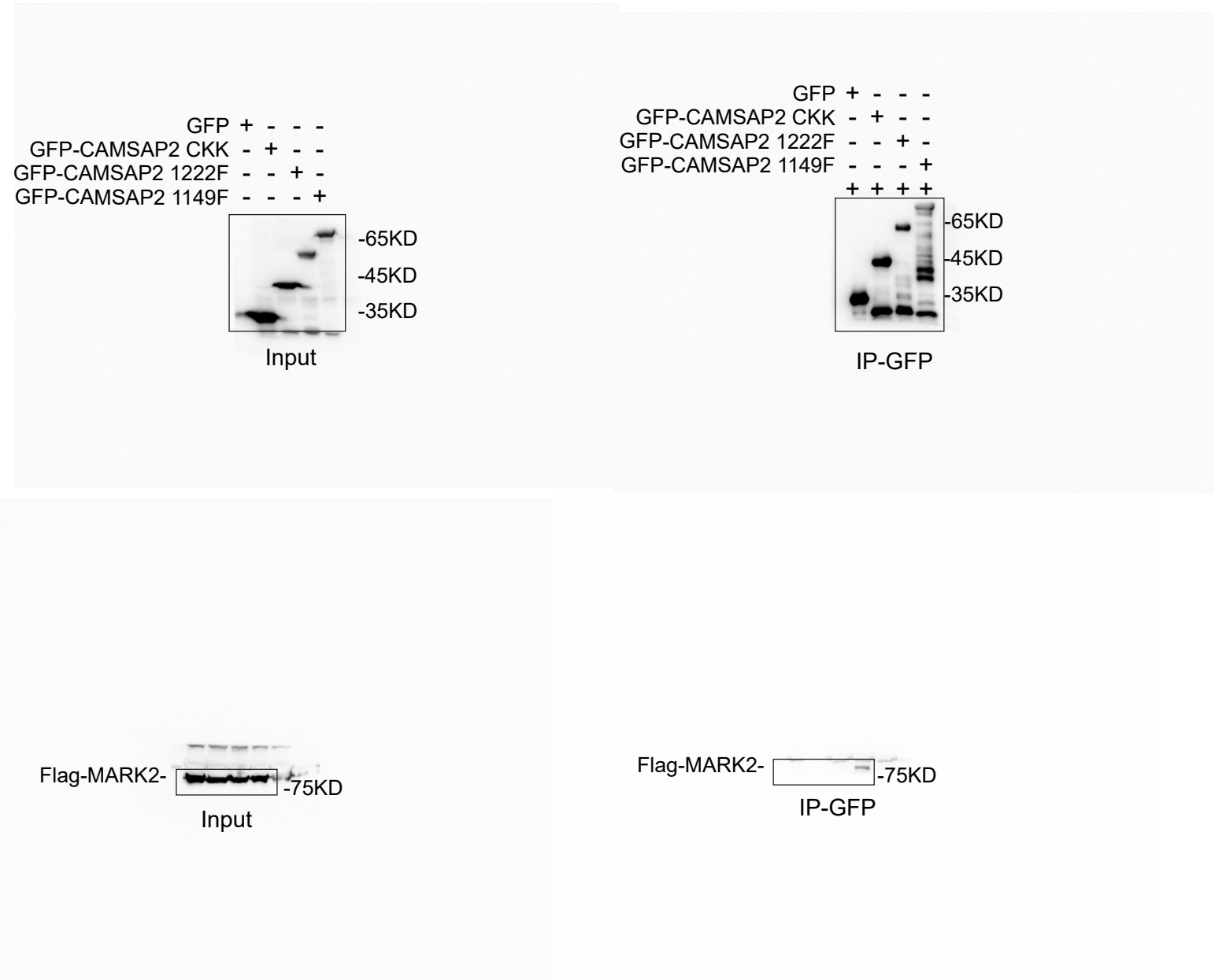

Supplement: Figure 2—figure supplement 1—source data 1. [file elife-105977-fig2-figsupp1-data1.zip › Figure 2-figure supplement 1 source data 1/Figure 2-figure supplement 1B source data 1.pdf]

Supplementary figure3F

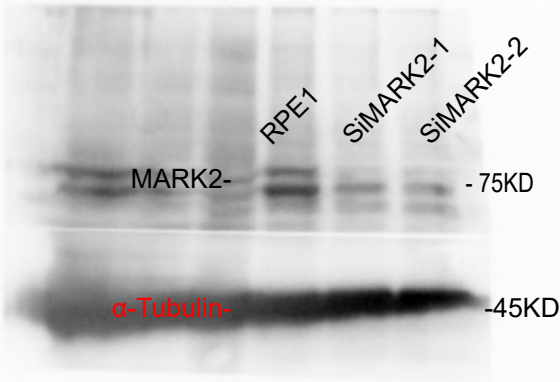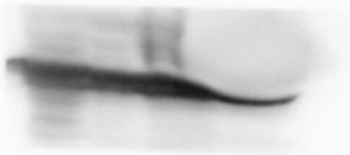

Supplement: Figure 2—figure supplement 1—source data 1. [file elife-105977-fig2-figsupp1-data1.zip › Figure 2-figure supplement 1 source data 1/Figure 2-figure supplement 1F source data 1.pdf]

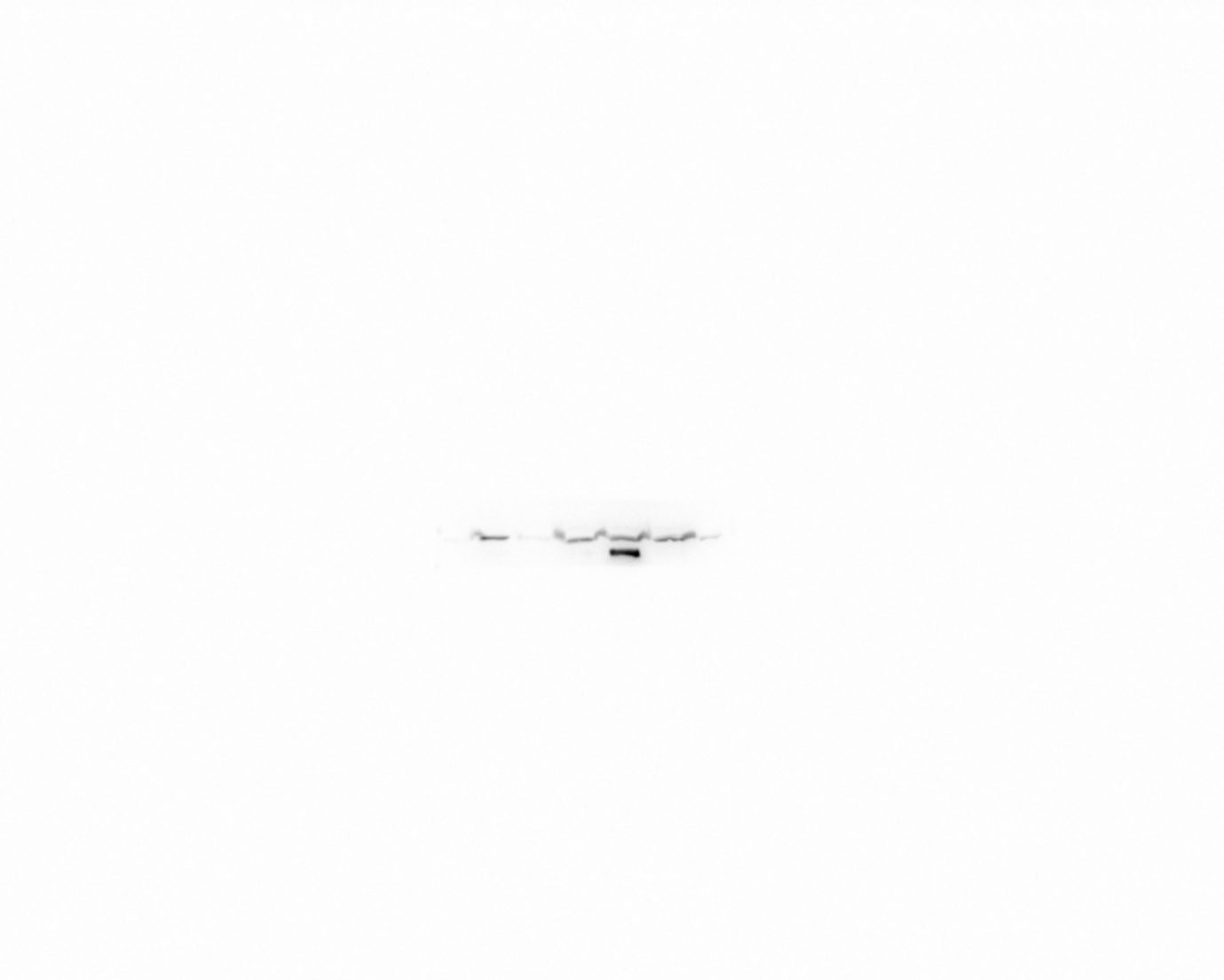

Supplement: Figure 2—figure supplement 1—source data 2. [file elife-105977-fig2-figsupp1-data2.zip › Figure 2-figure supplement 1 source data 2/Figure 2-figure supplement 1B source data 2/IP-Flag-Cam20241002_121544_opt-1.tif]

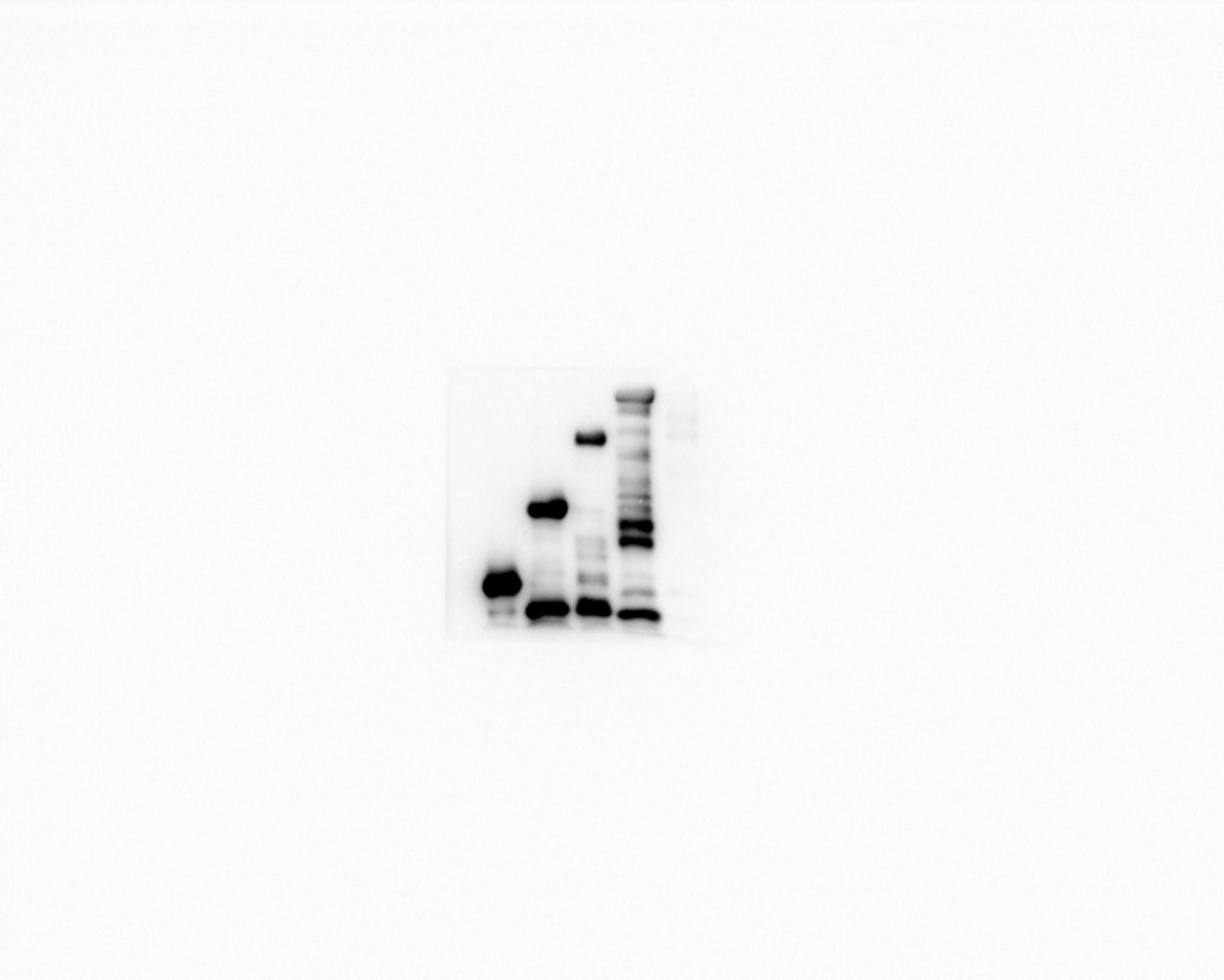

Supplement: Figure 2—figure supplement 1—source data 2. [file elife-105977-fig2-figsupp1-data2.zip › Figure 2-figure supplement 1 source data 2/Figure 2-figure supplement 1B source data 2/IP-GFP-Cam20241002_120558_opt-10.tif]

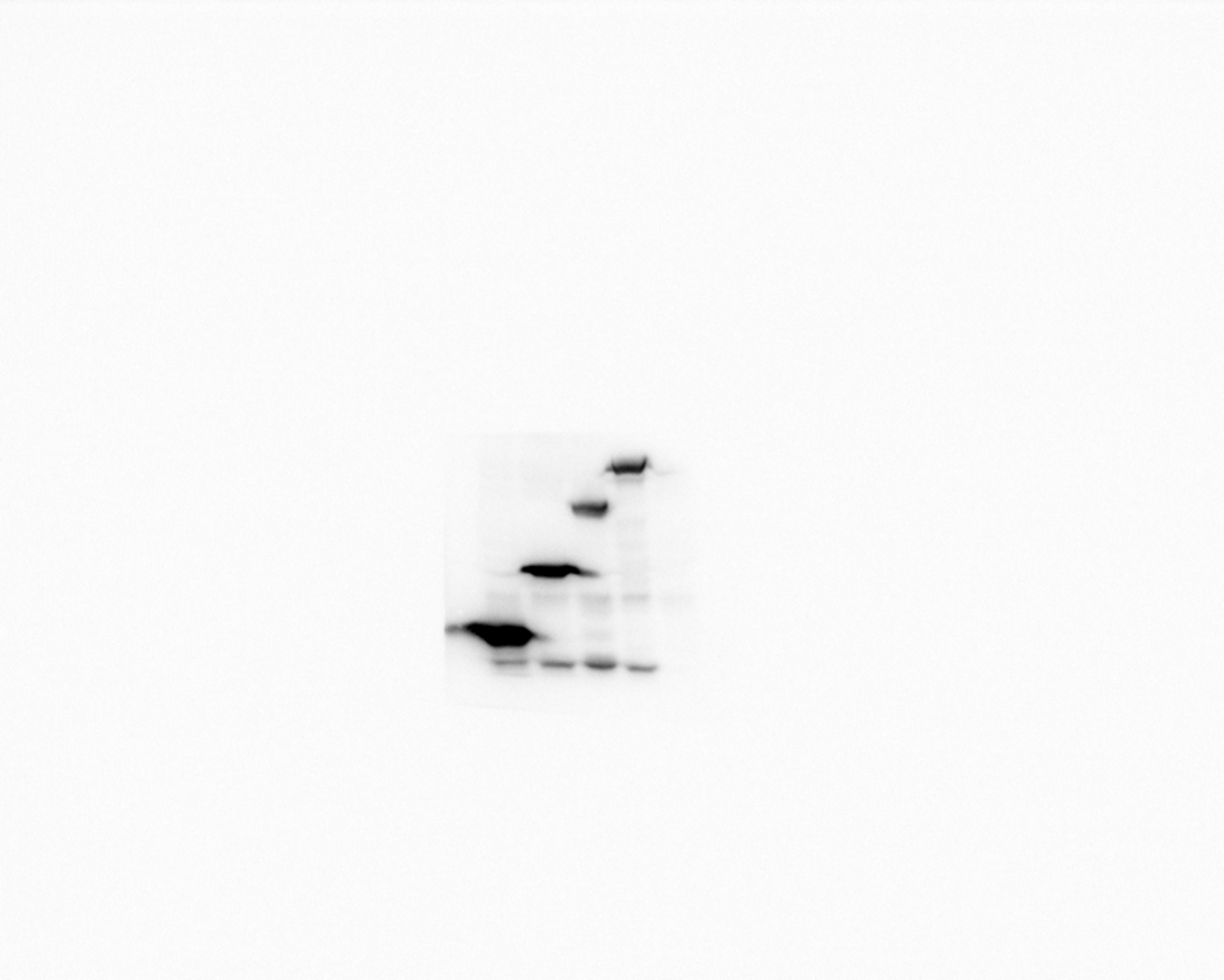

Supplement: Figure 2—figure supplement 1—source data 2. [file elife-105977-fig2-figsupp1-data2.zip › Figure 2-figure supplement 1 source data 2/Figure 2-figure supplement 1B source data 2/Input-gfp-Cam20241002_120319_opt-10.tif]

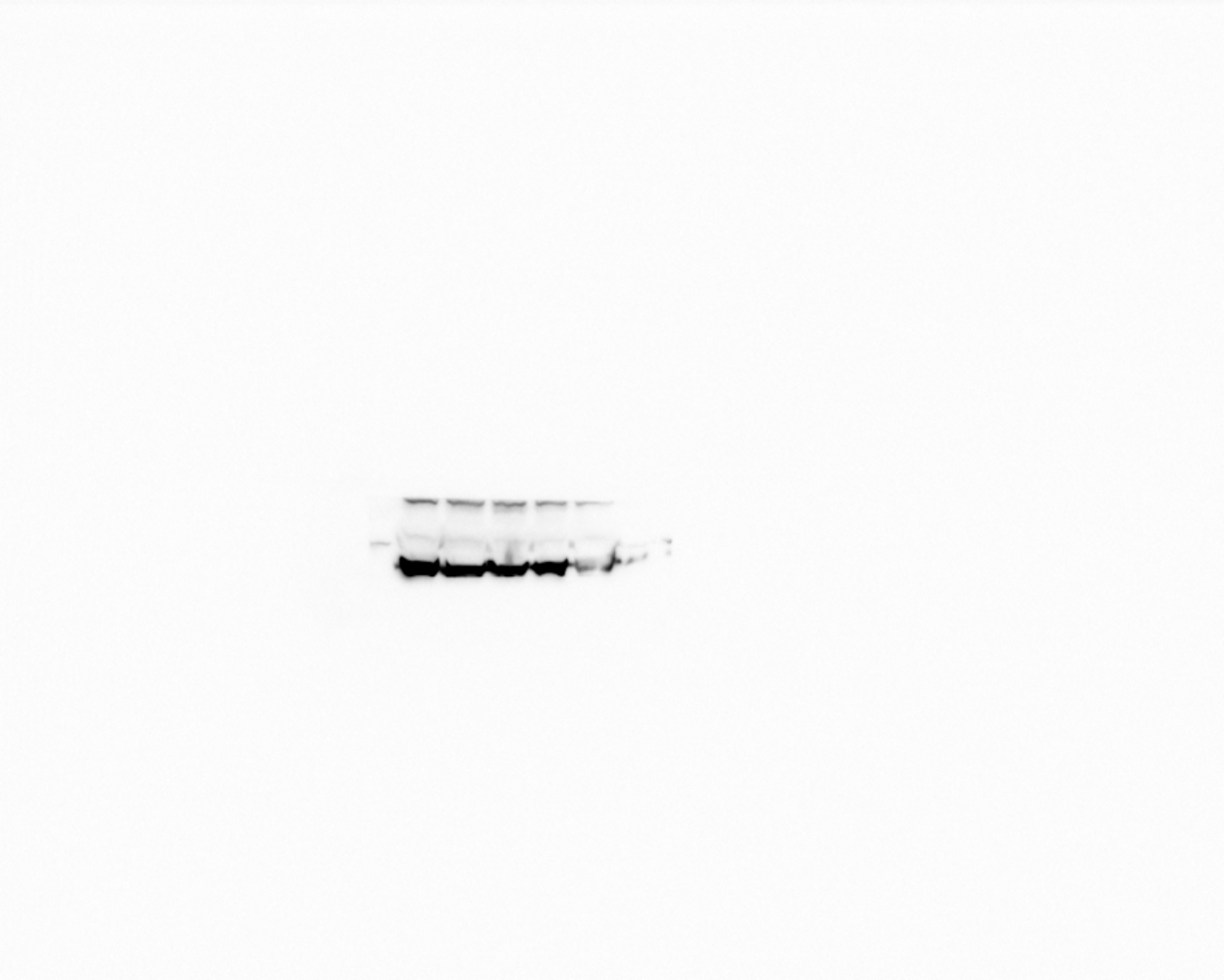

Supplement: Figure 2—figure supplement 1—source data 2. [file elife-105977-fig2-figsupp1-data2.zip › Figure 2-figure supplement 1 source data 2/Figure 2-figure supplement 1B source data 2/input-Flag-Cam20241002_120851_opt-7.tif]

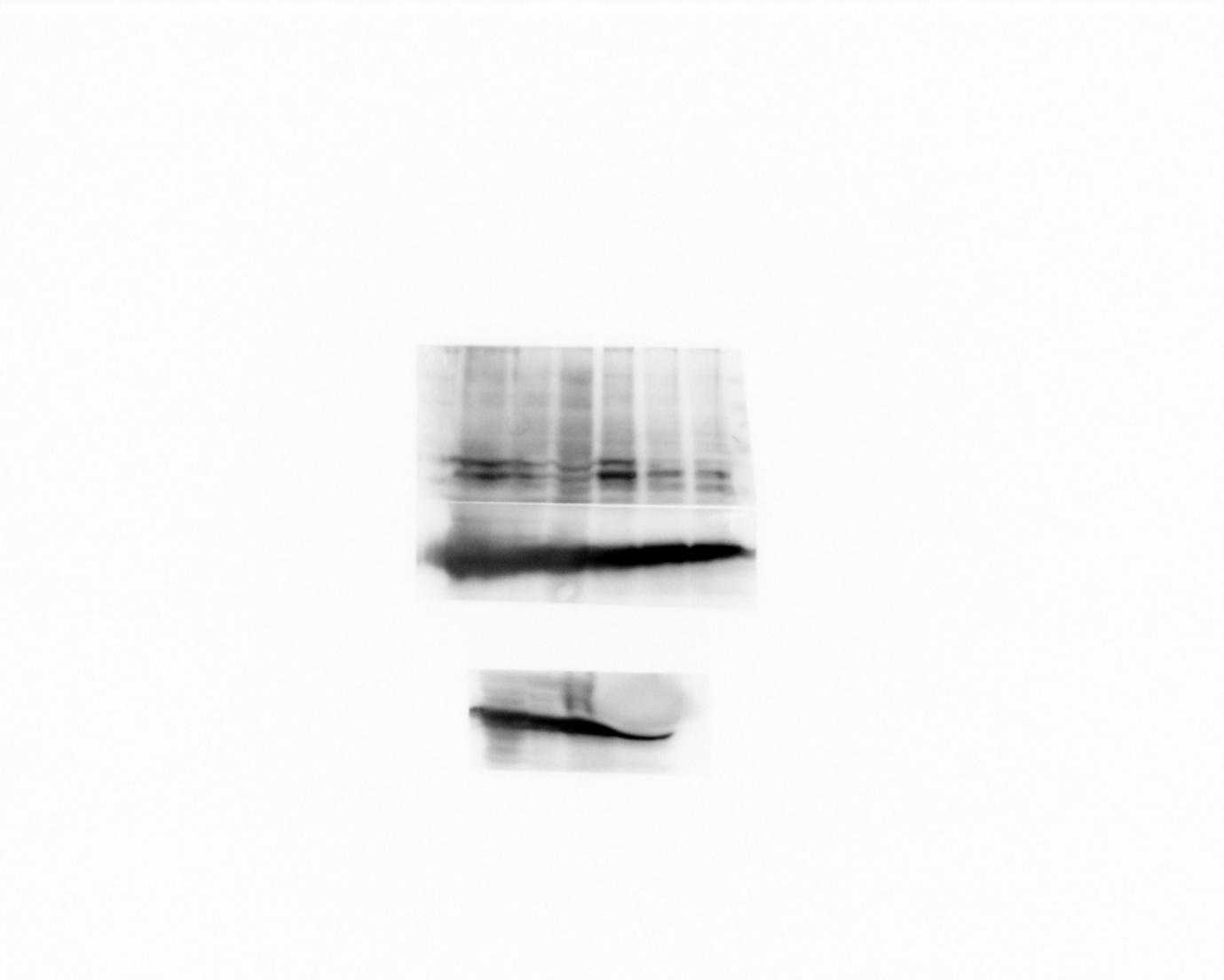

Supplement: Figure 2—figure supplement 1—source data 2. [file elife-105977-fig2-figsupp1-data2.zip › Figure 2-figure supplement 1 source data 2/Figure 2-figure supplement 1F source data 2/A-Cam20210609_103236_opt_5.TIF]

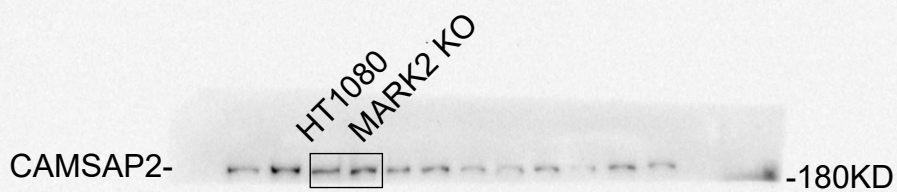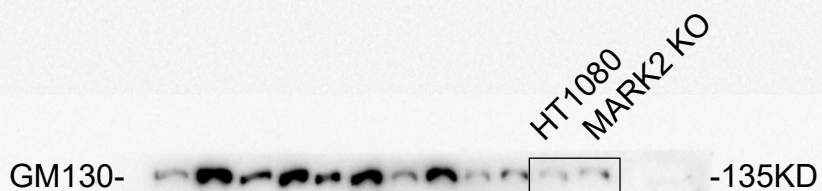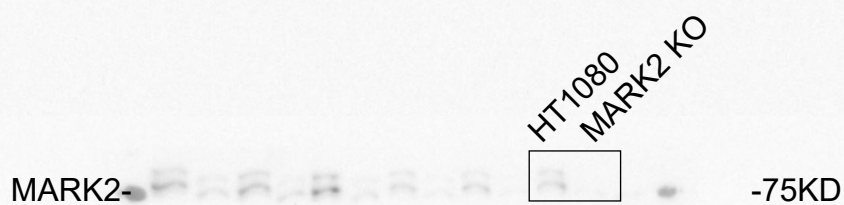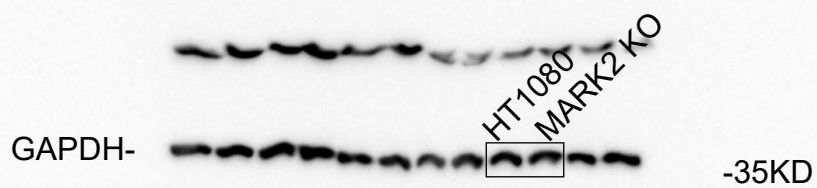

Supplement: Figure 3—source data 1. [file elife-105977-fig3-data1.zip › Figure 3 - source data 1/Figure3D-source-data1-PDF/Figure3Dí¬PDF.pdf]

Figure3H

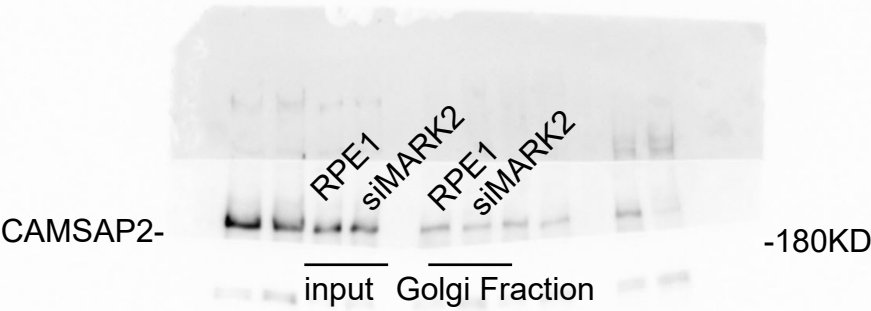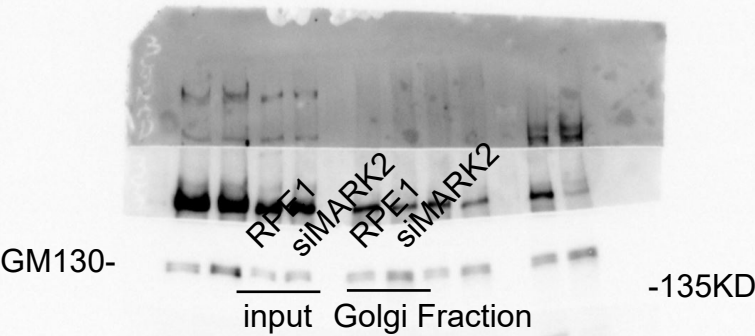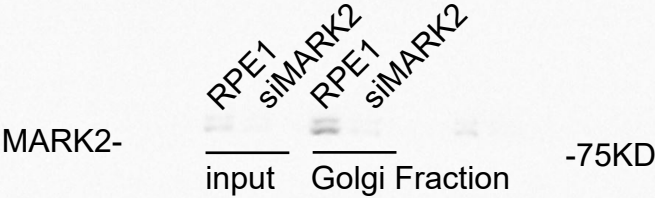

Supplement: Figure 3—source data 1. [file elife-105977-fig3-data1.zip › Figure 3 - source data 1/Figure3H-source-data1-PDF/3H.pdf]

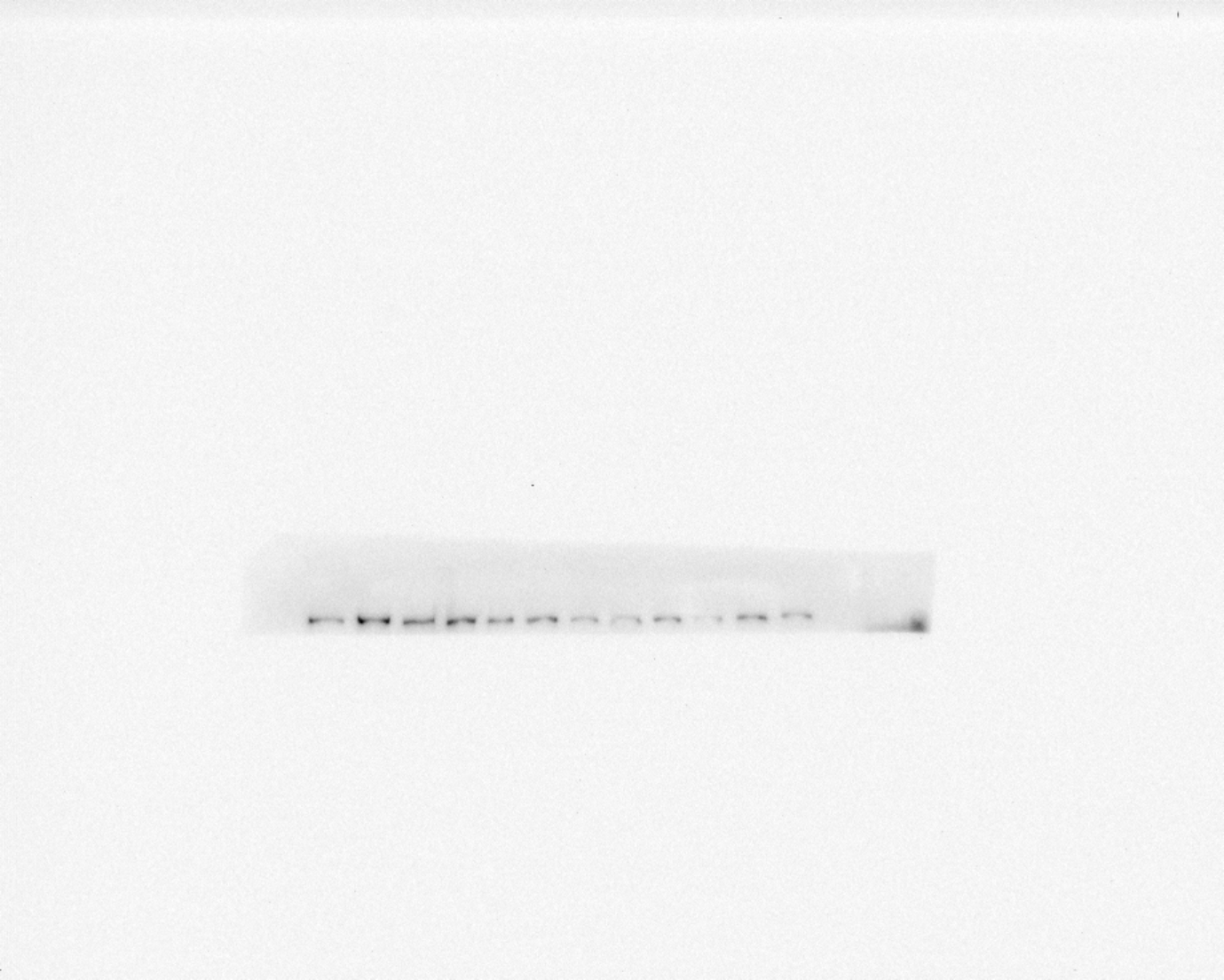

Supplement: Figure 3—source data 2. [file elife-105977-fig3-data2.zip › Figure 3 - source data 2/Figure3D-source-2/A-CAMSAP2-Cam20230926_110009_opt_25.TIF]

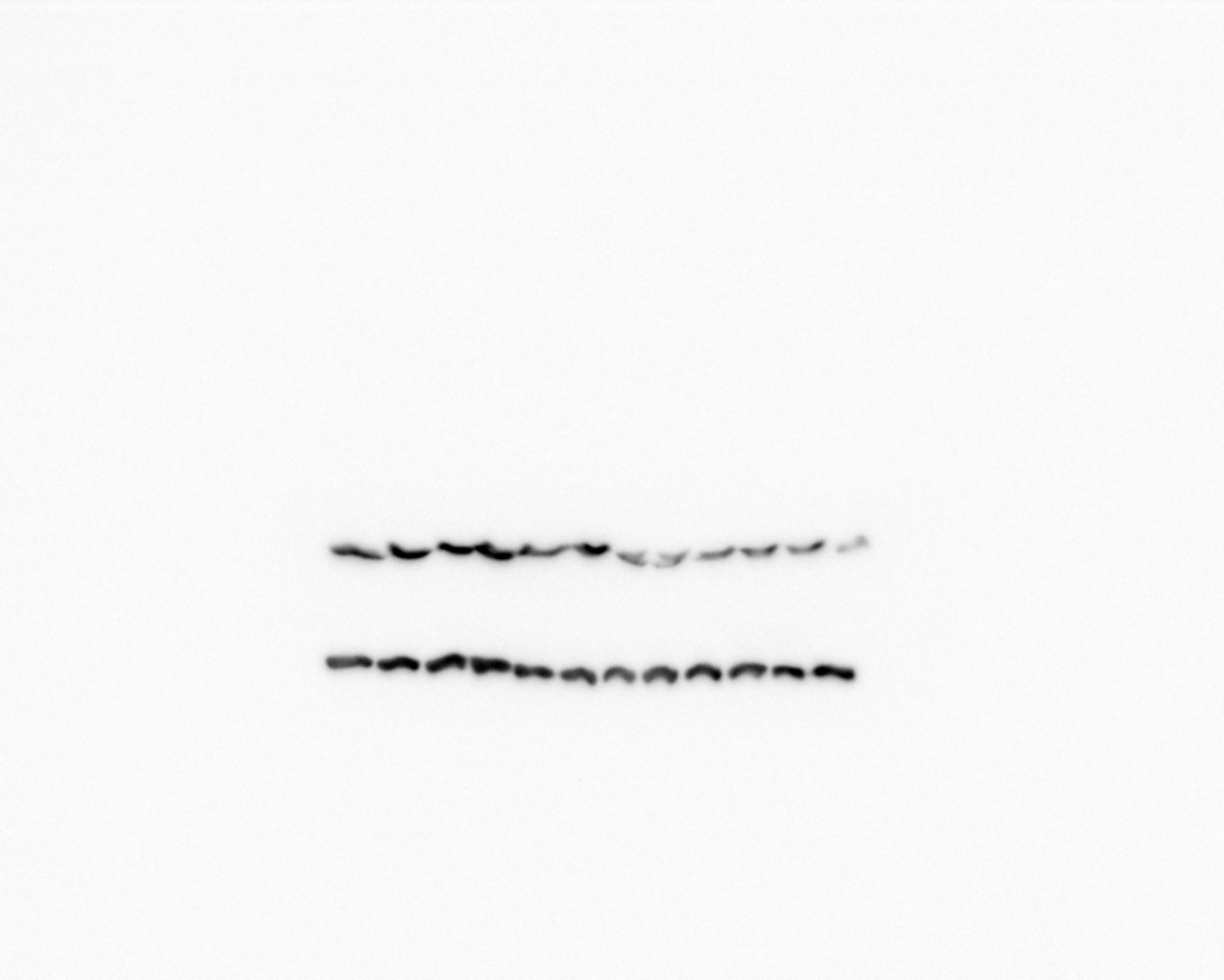

Supplement: Figure 3—source data 2. [file elife-105977-fig3-data2.zip › Figure 3 - source data 2/Figure3D-source-2/A-GAPDH-Cam20230926_105427_opt_14.TIF]

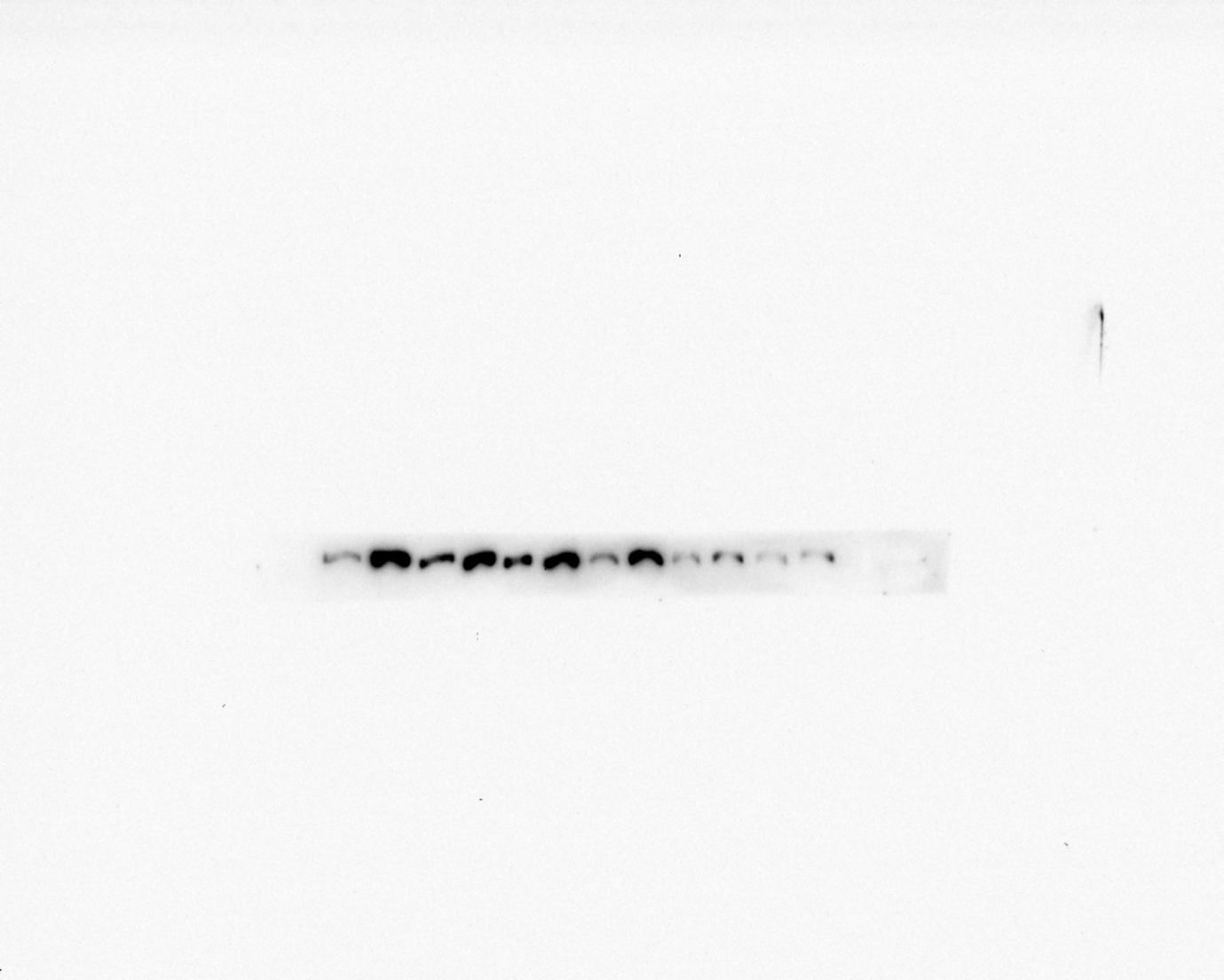

Supplement: Figure 3—source data 2. [file elife-105977-fig3-data2.zip › Figure 3 - source data 2/Figure3D-source-2/A-GM130-Cam20230926_110532_opt_20.TIF]

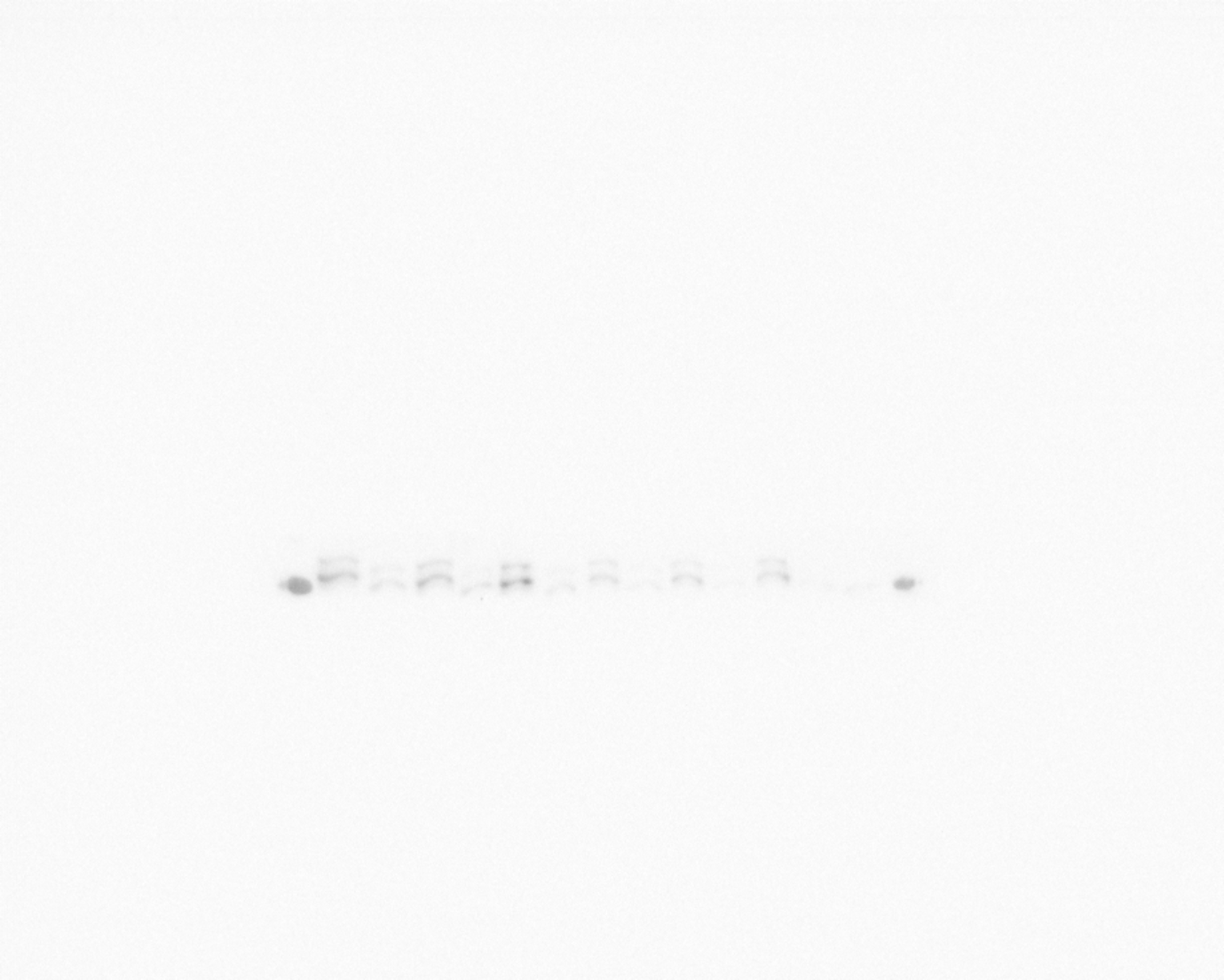

Supplement: Figure 3—source data 2. [file elife-105977-fig3-data2.zip › Figure 3 - source data 2/Figure3D-source-2/A-MARK2-Cam20230926_105150_opt_14.TIF]

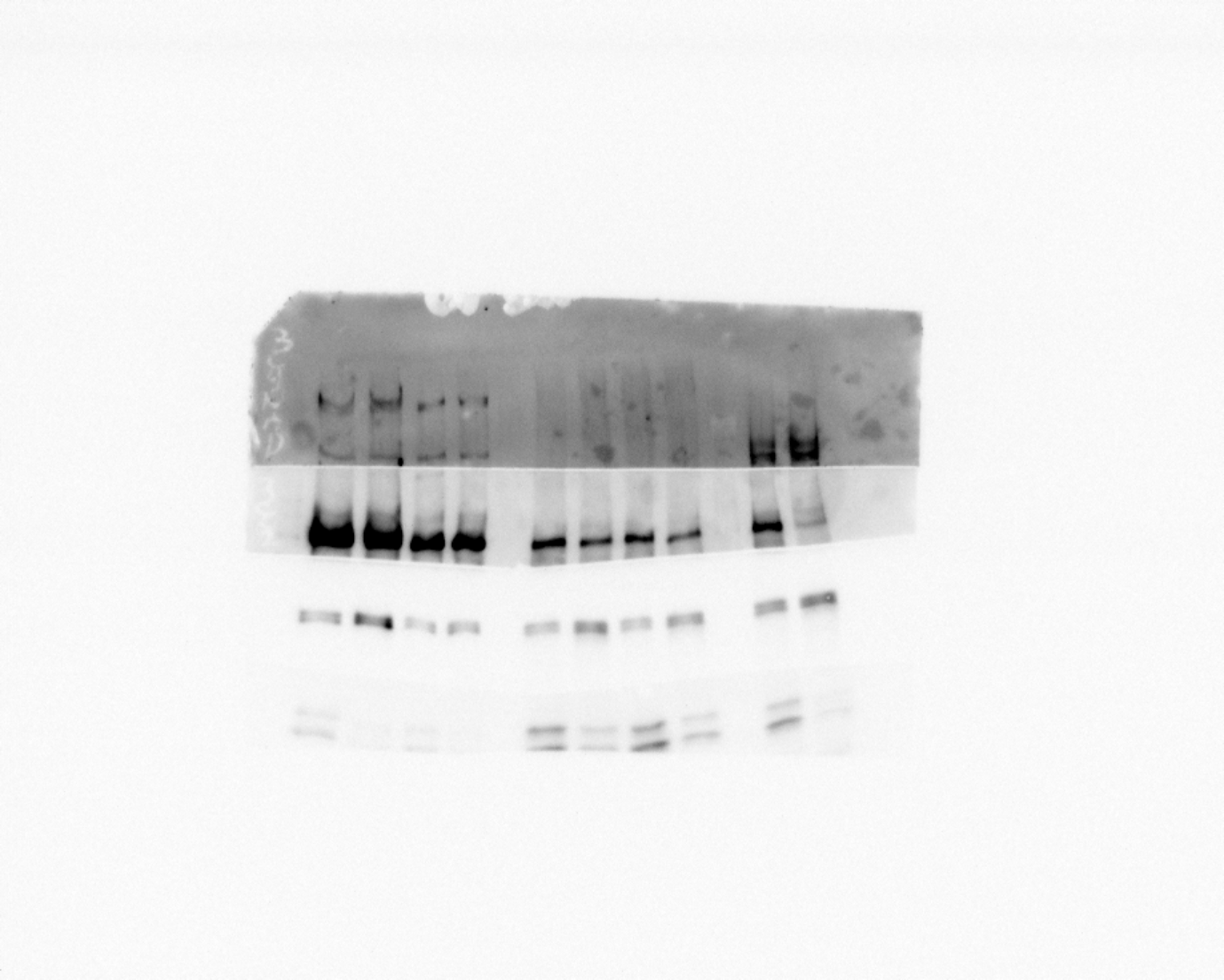

Supplement: Figure 3—source data 2. [file elife-105977-fig3-data2.zip › Figure 3 - source data 2/Figure3H-source-2/A-CAMSAP2-GM130-Cam20220617_165643_opt_16.TIF]

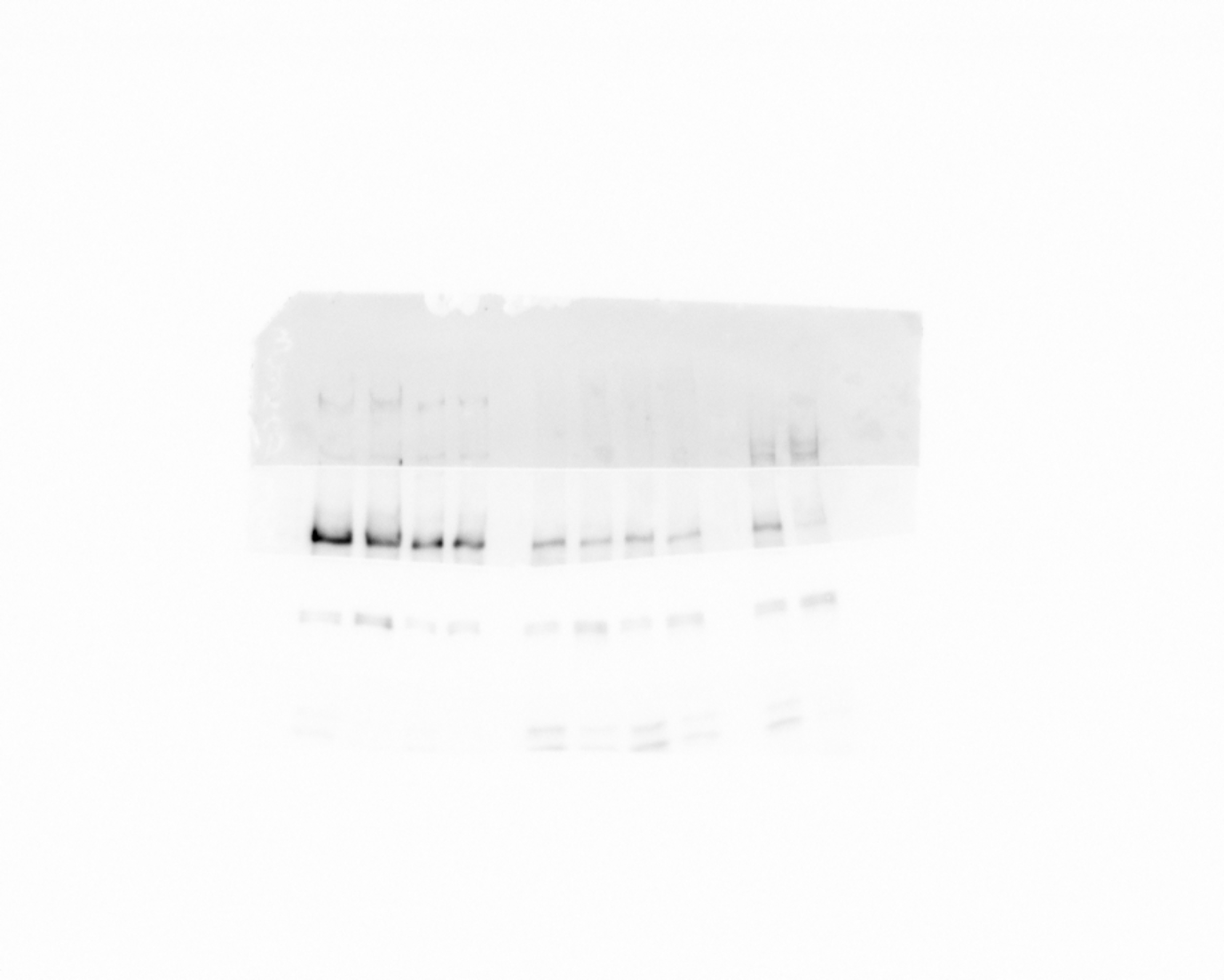

Supplement: Figure 3—source data 2. [file elife-105977-fig3-data2.zip › Figure 3 - source data 2/Figure3H-source-2/A-CAMSAP2-GM130-Cam20220617_165643_opt_4.TIF]

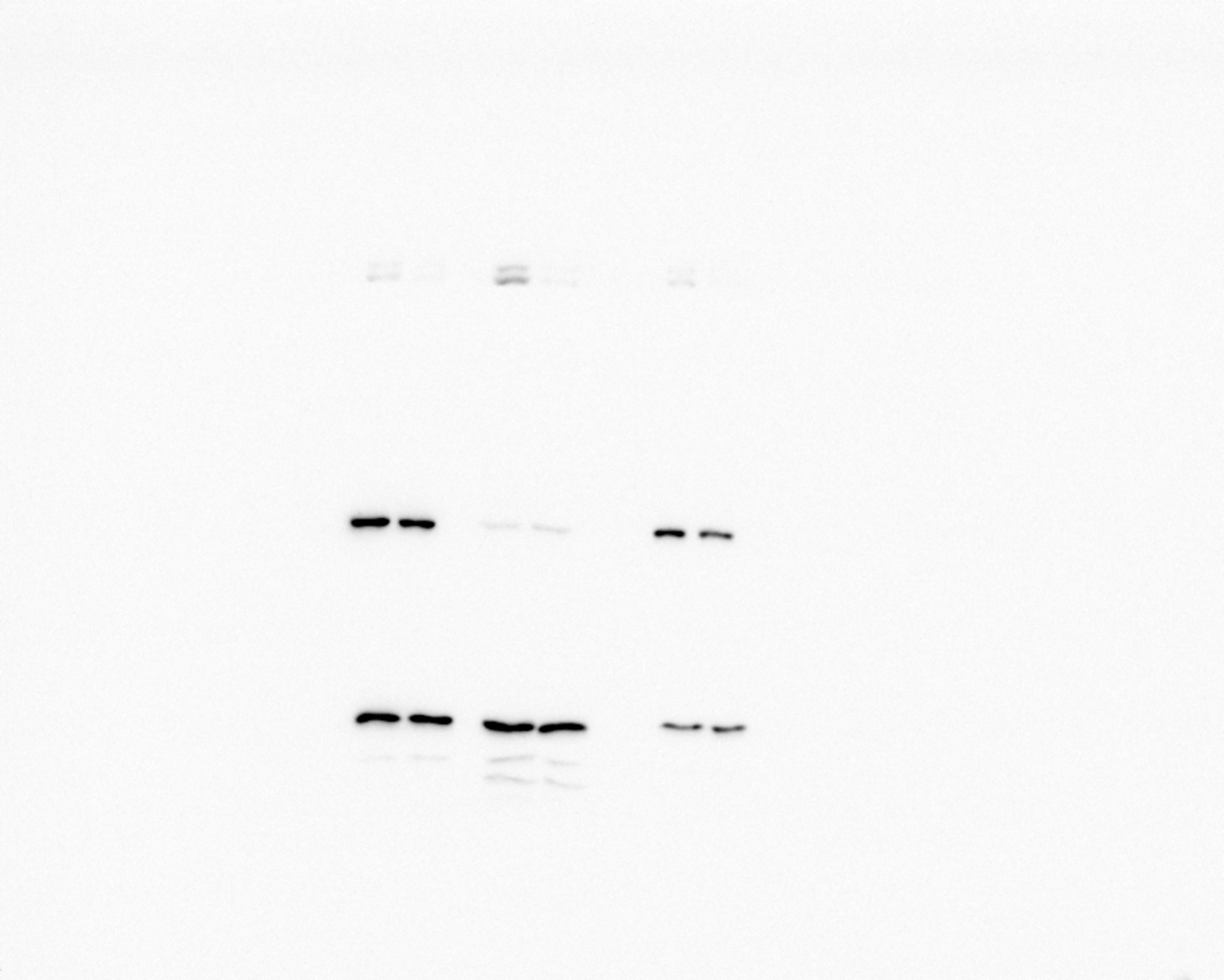

Supplement: Figure 3—source data 2. [file elife-105977-fig3-data2.zip › Figure 3 - source data 2/Figure3H-source-2/A-MARK2-Cam20220622_171847_opt_16.TIF]

|                 |   |   |   |
|-----------------|---|---|---|
| GST             | + | - | - |
| GST-MARK2       | - | + | - |
| His-GFP-CAMSAP2 | - | - | + |

His-GFP-CAMSAP2-

GST-MARK2-

GST-

-180KD  
-135KD  
-100KD  
-75KD  
-65KD  
-45KD  
-35KD  
-25KD

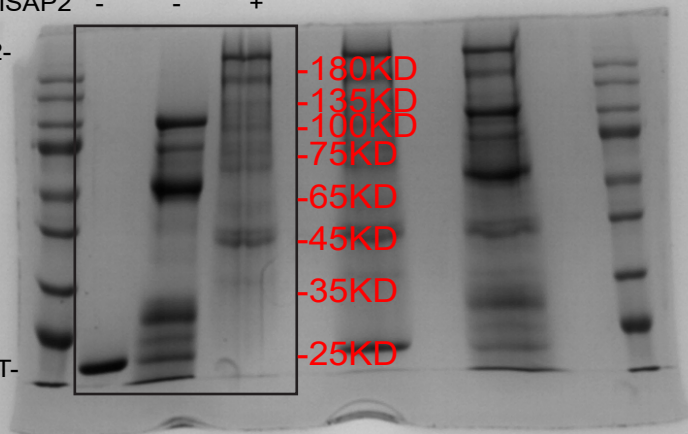

Supplement: Figure 3—figure supplement 1—source data 1. [file elife-105977-fig3-figsupp1-data1.zip › Figure 3-figure supplement 1 source data 1/Figure 3-figure supplement 1A source data 1/Figure 3-figure supplement 1A source data .pdf]

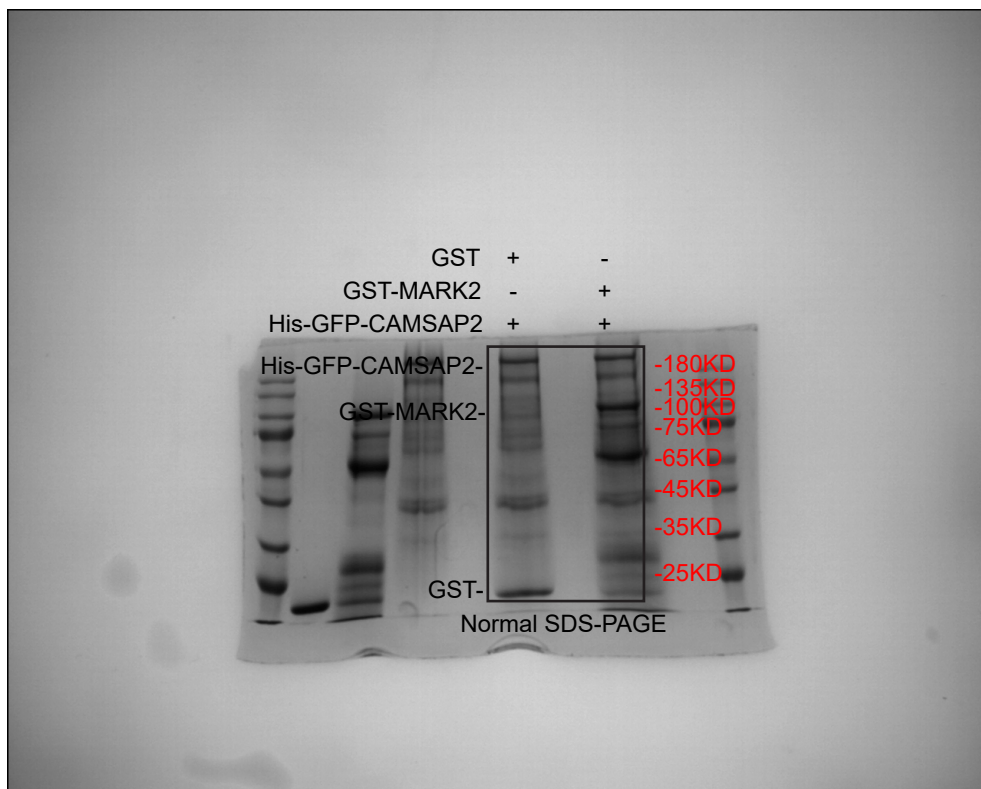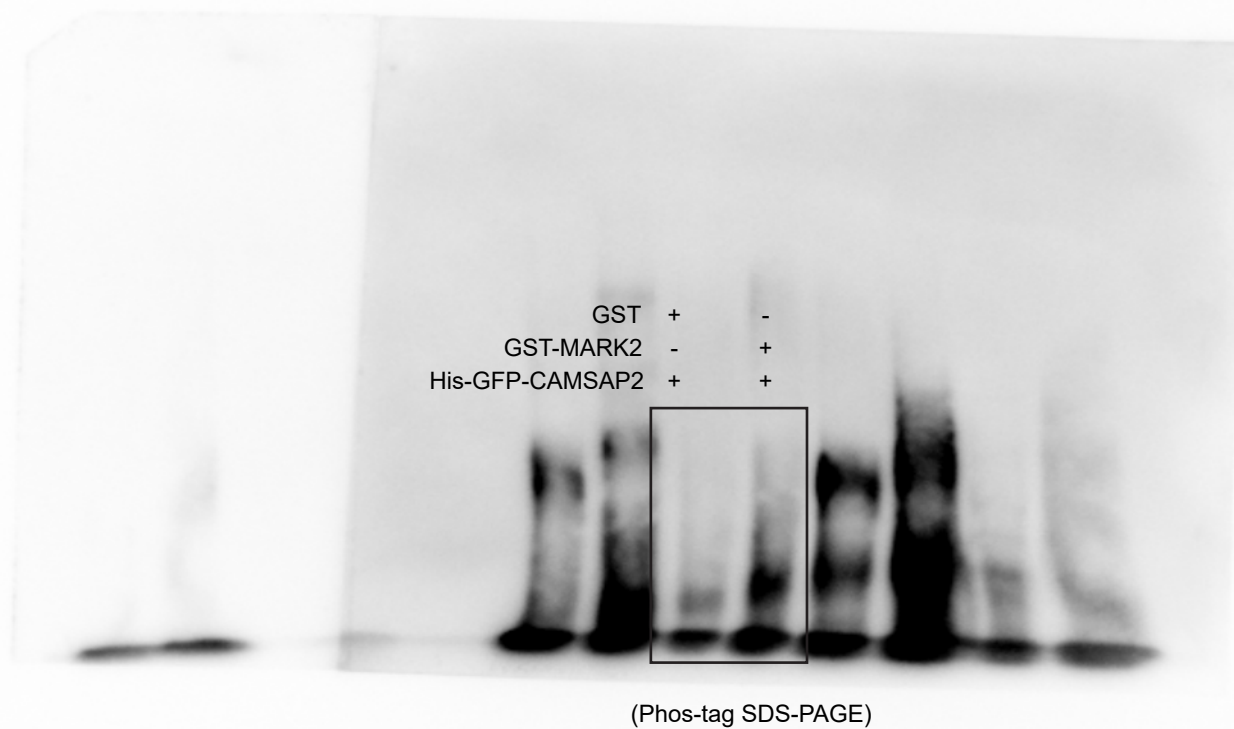

Supplement: Figure 3—figure supplement 1—source data 1. [file elife-105977-fig3-figsupp1-data1.zip › Figure 3-figure supplement 1 source data 1/Figure 3-figure supplement 1B source data 1/Figure 3-figure supplement 1B source data .pdf]

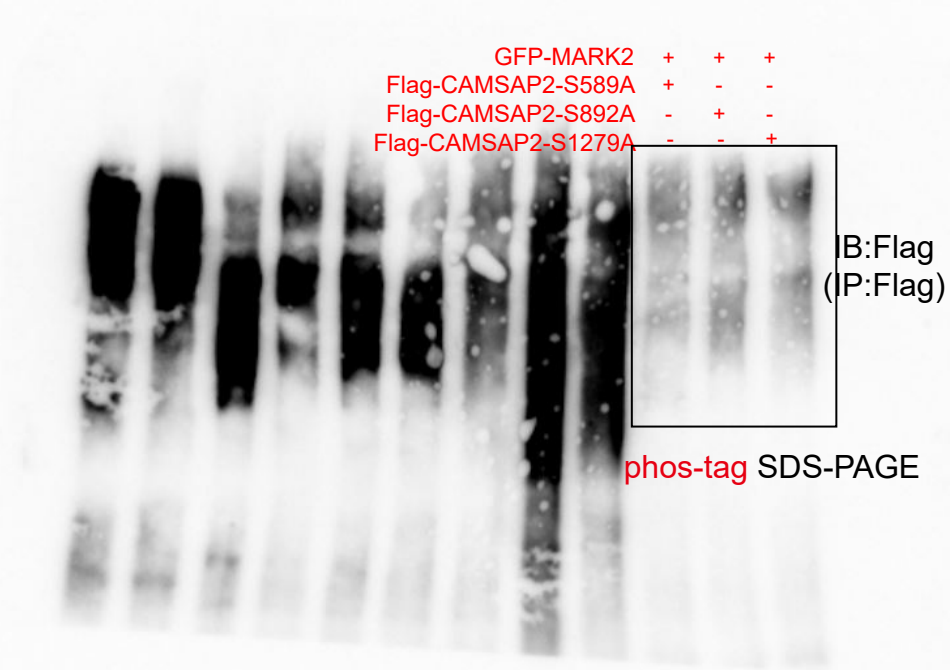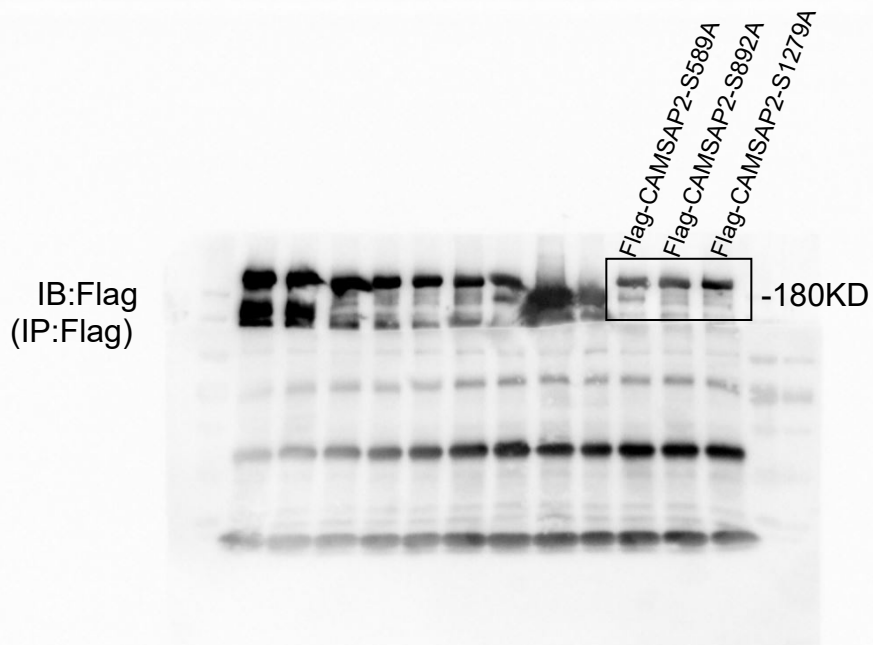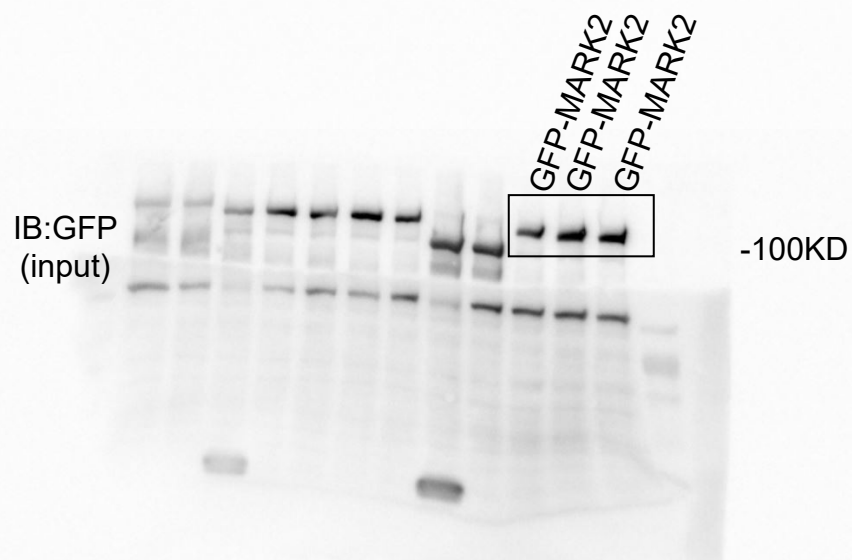

Supplement: Figure 3—figure supplement 1—source data 1. [file elife-105977-fig3-figsupp1-data1.zip › Figure 3-figure supplement 1 source data 1/Figure 3-figure supplement 1C source data 1/Figure 3-figure supplement 1C source data .pdf]

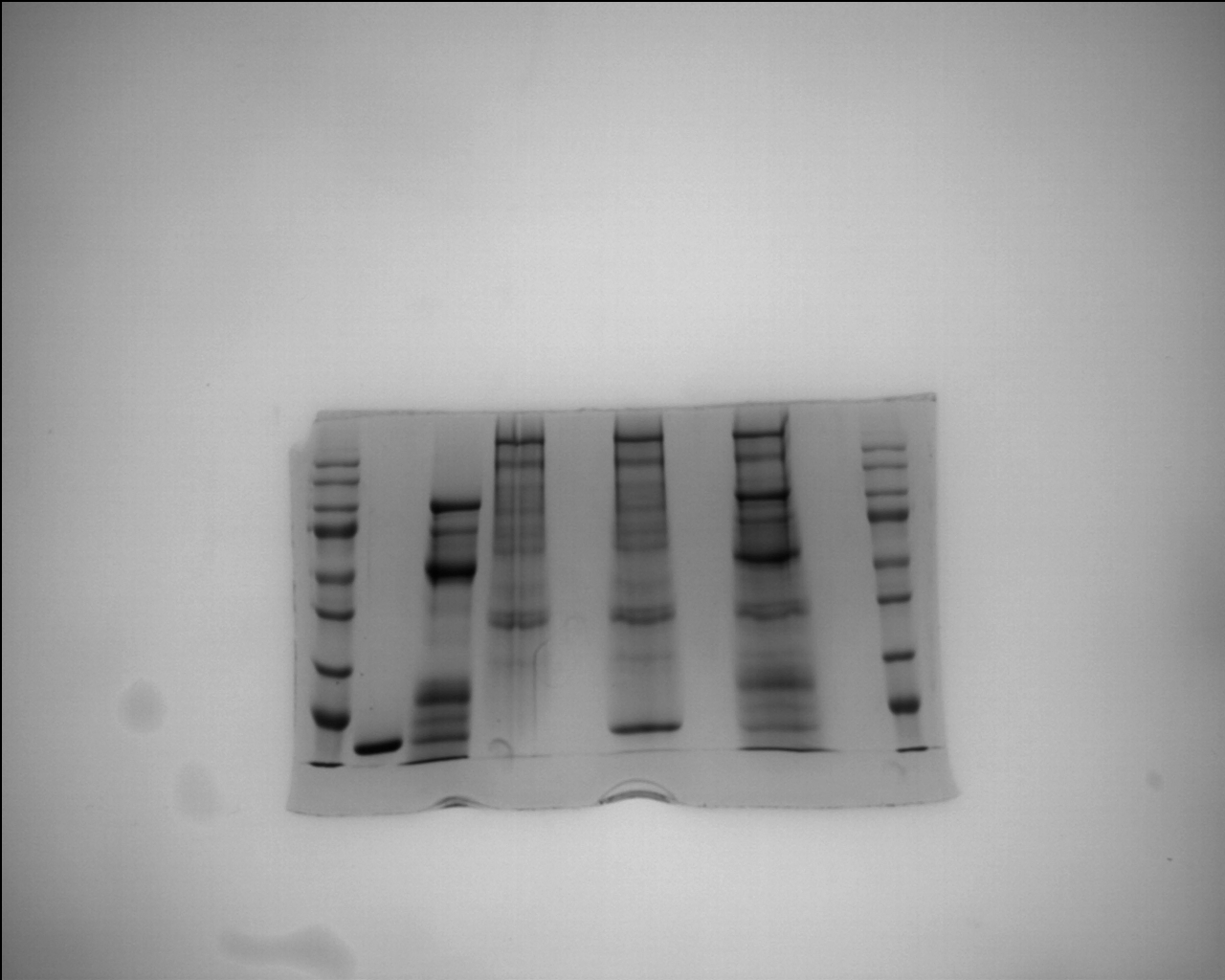

Supplement: Figure 3—figure supplement 1—source data 2. [file elife-105977-fig3-figsupp1-data2.zip › Figure 3-figure supplement 1 source data 2/Figure 3-figure supplement 1A source data 2/231210-2.tif]

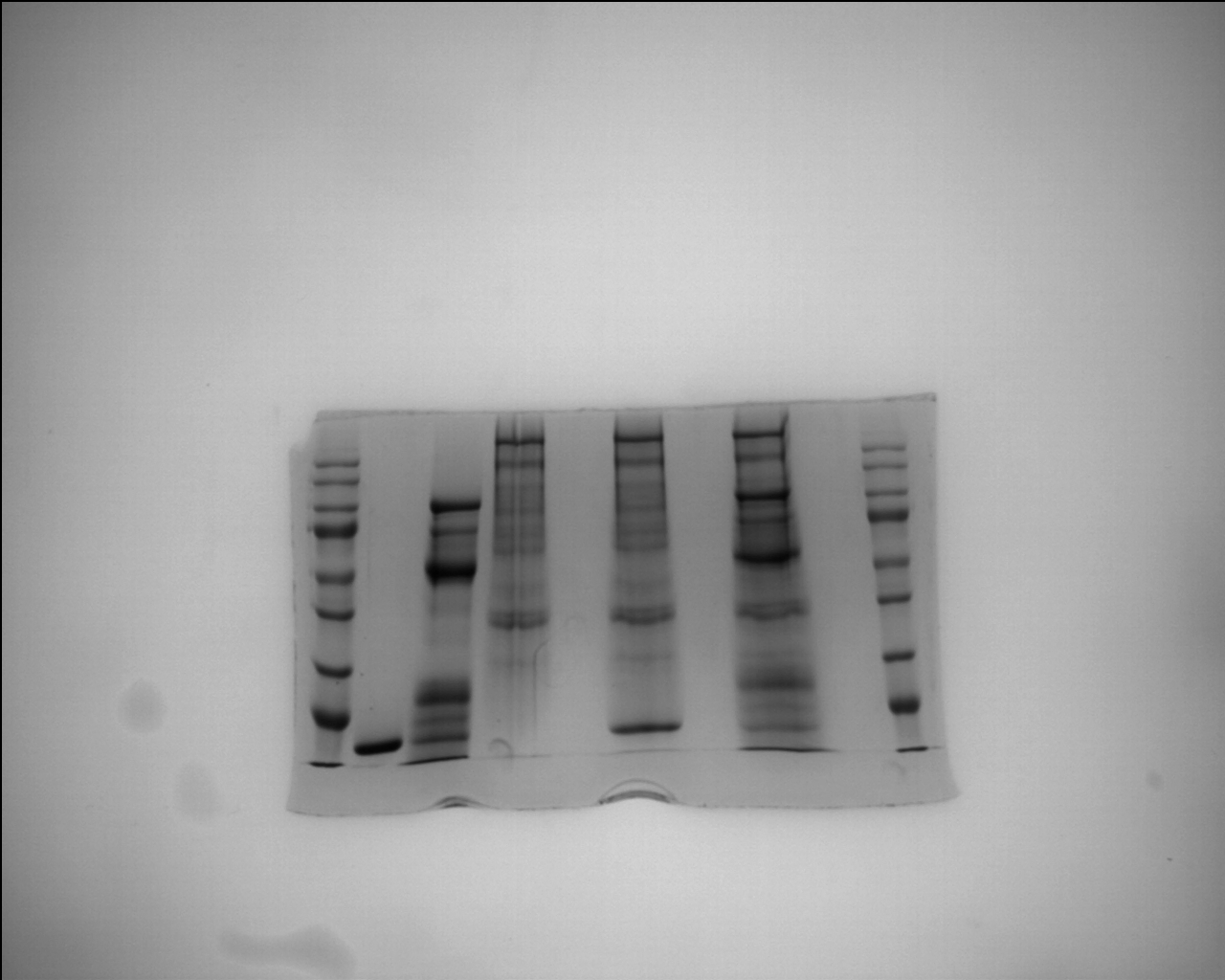

Supplement: Figure 3—figure supplement 1—source data 2. [file elife-105977-fig3-figsupp1-data2.zip › Figure 3-figure supplement 1 source data 2/Figure 3-figure supplement 1A source data 2/231210-2_1.TIF]

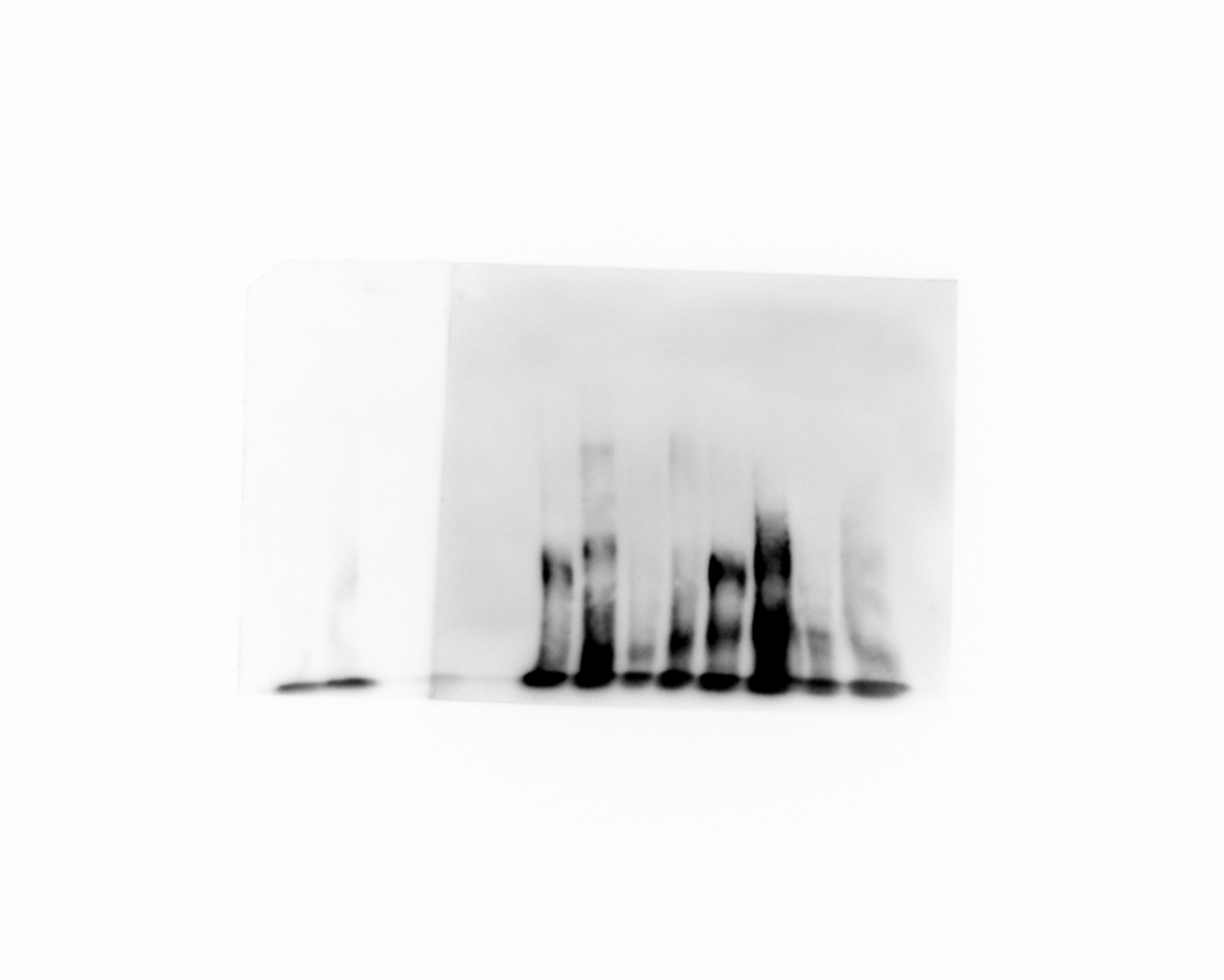

Supplement: Figure 3—figure supplement 1—source data 2. [file elife-105977-fig3-figsupp1-data2.zip › Figure 3-figure supplement 1 source data 2/Figure 3-figure supplement 4B source data 2/A-Cam20200819_115300_opt_2.TIF]

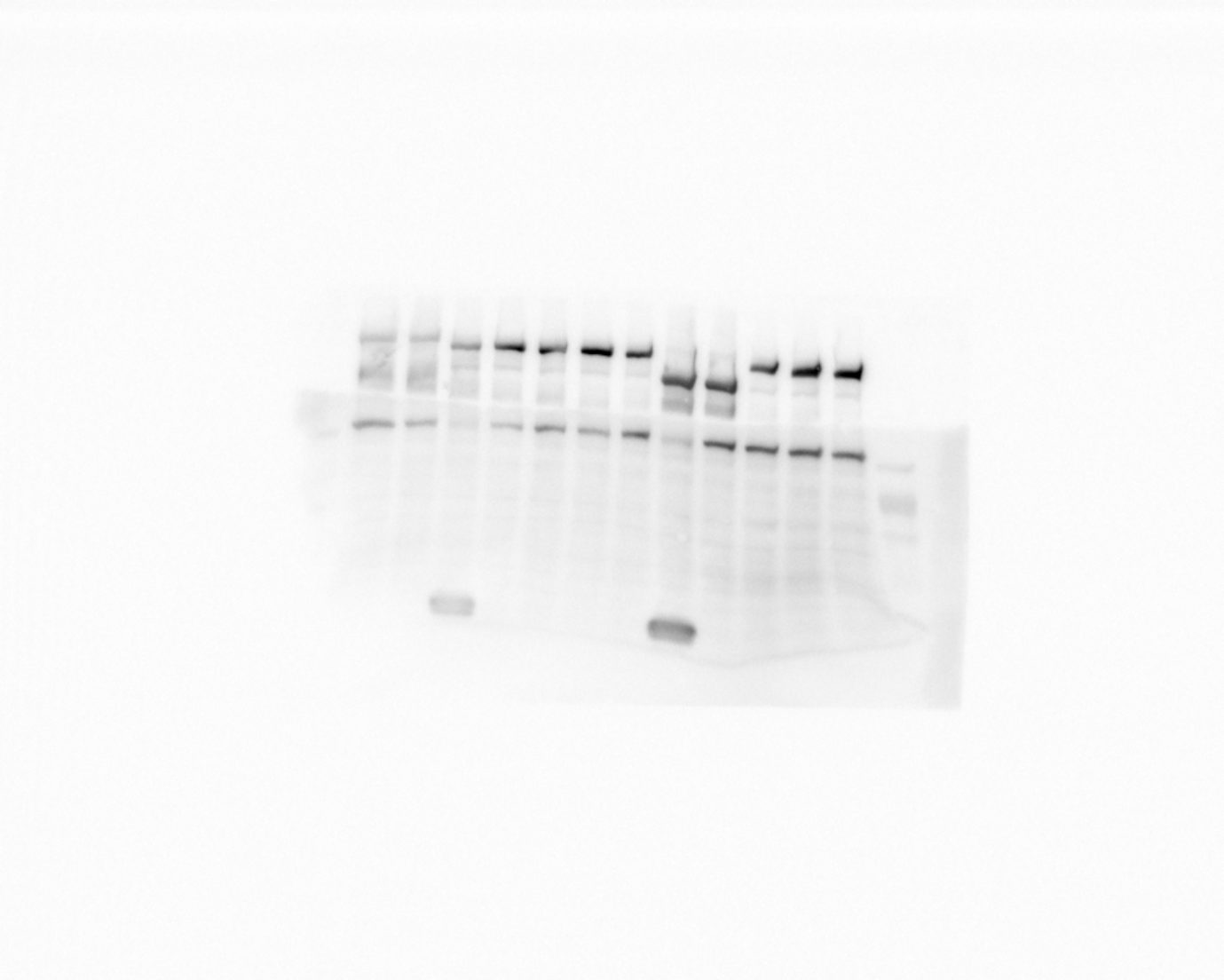

Supplement: Figure 3—figure supplement 1—source data 2. [file elife-105977-fig3-figsupp1-data2.zip › Figure 3-figure supplement 1 source data 2/Figure 3-figure supplement 4C source data 2/A-INPUT-GFP-MARK2-GFP-Cam20210202_115648_opt_9.TIF]

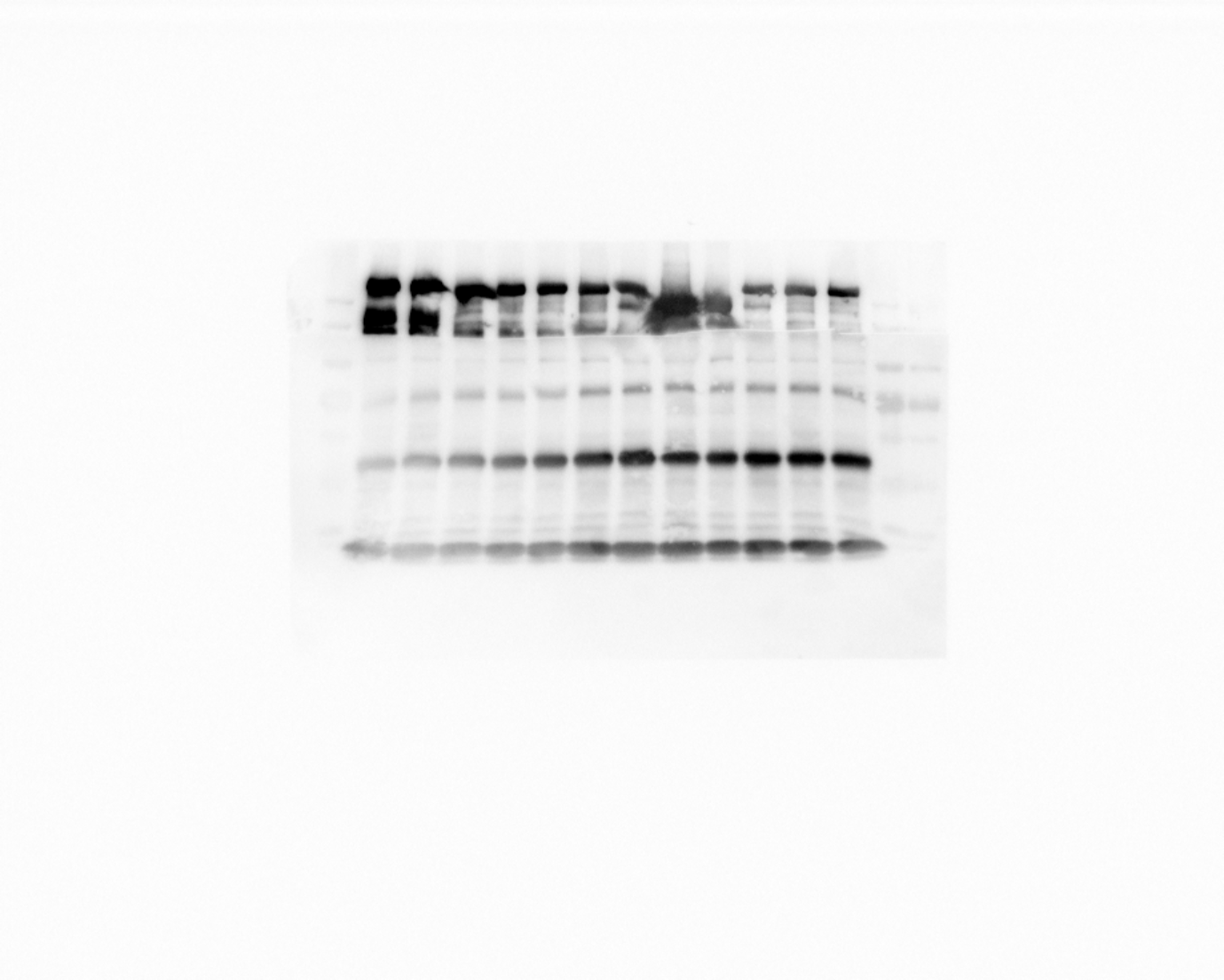

Supplement: Figure 3—figure supplement 1—source data 2. [file elife-105977-fig3-figsupp1-data2.zip › Figure 3-figure supplement 1 source data 2/Figure 3-figure supplement 4C source data 2/A-IP-FLAG-CAMSAP2-Cam20210202_115031_opt_7.TIF]

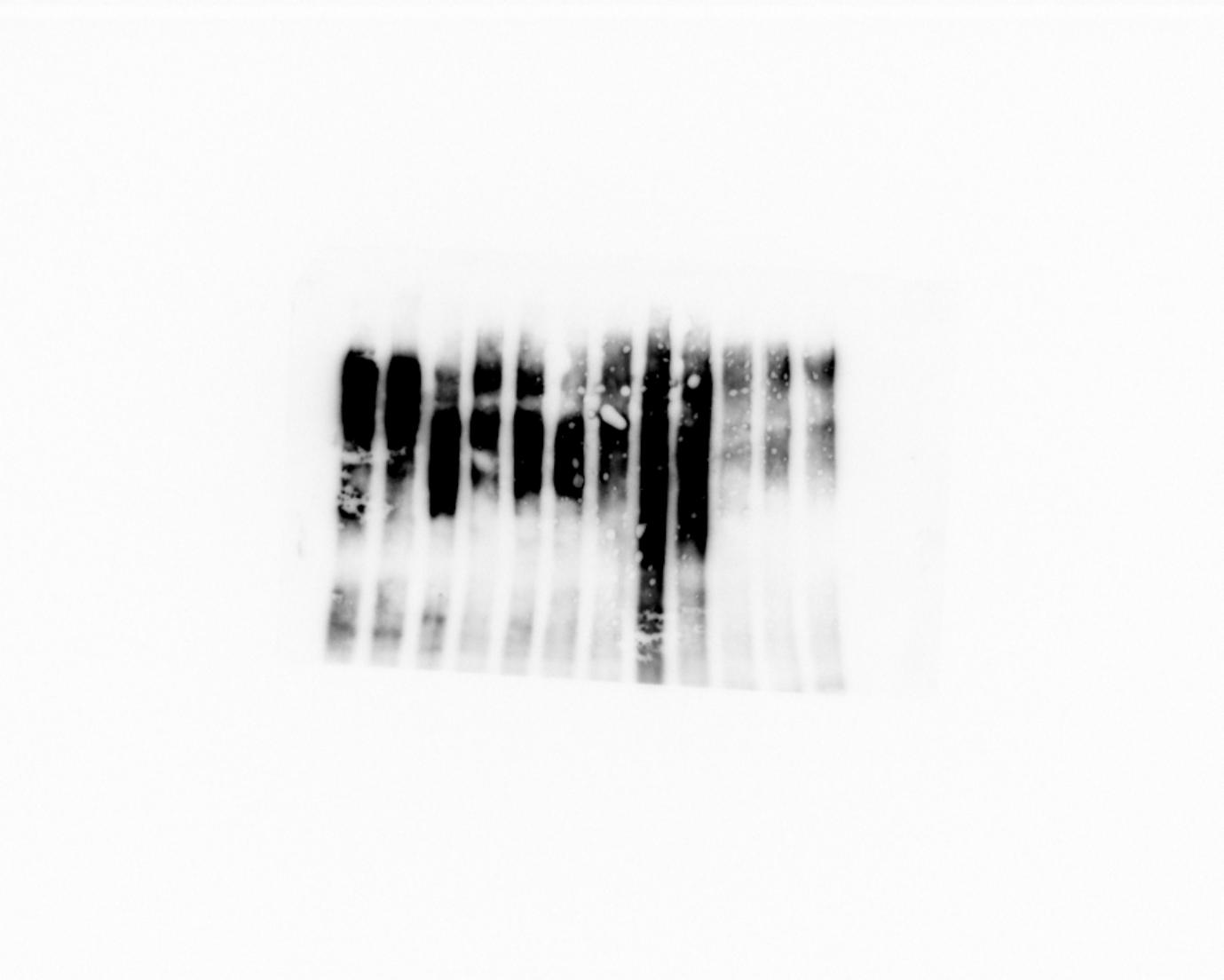

Supplement: Figure 3—figure supplement 1—source data 2. [file elife-105977-fig3-figsupp1-data2.zip › Figure 3-figure supplement 1 source data 2/Figure 3-figure supplement 4C source data 2/A-Phostag-Cam20210202_114206_opt_8.TIF]

Figure 4B

|               |   |   |   |   |
|---------------|---|---|---|---|
| GFP           | + | - | - | - |
| GFP-MARK2     | - | + | + | + |
| Flag-CAMSAP2  | + | + | - | - |
| Flag-CS2-397A | - | - | + | - |
| Flag-CS2-835A | - | - | - | + |

IB:Flag  
(IP:Flag)

phos-tag SDS-PAGE

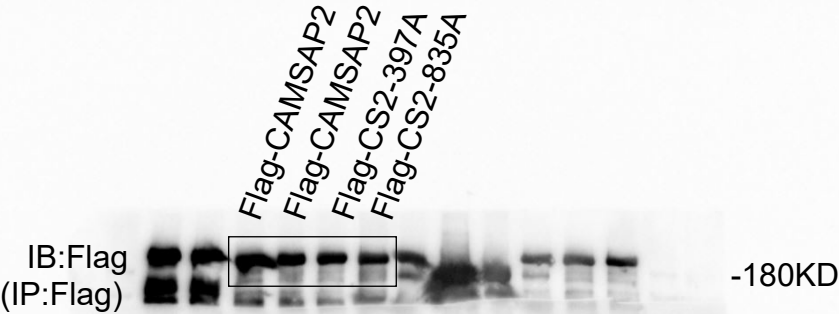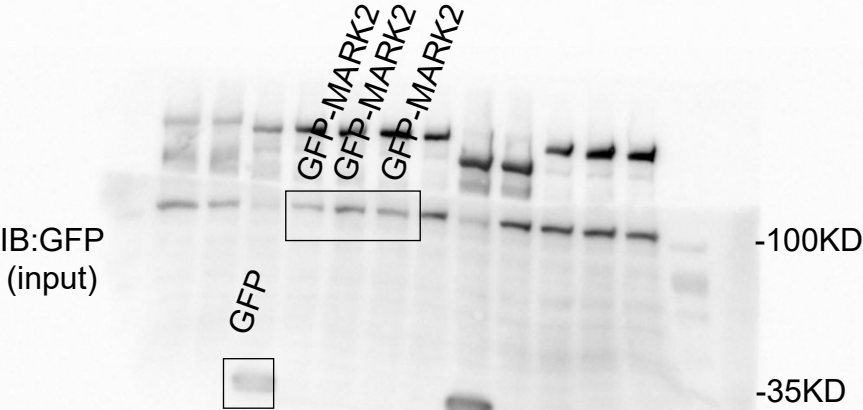

Supplement: Figure 4—source data 1. [file elife-105977-fig4-data1.zip › Figure 4 - Source data 1/Figure4B-source data 1-PDF/Figure4B-source data 1-PDF.pdf]

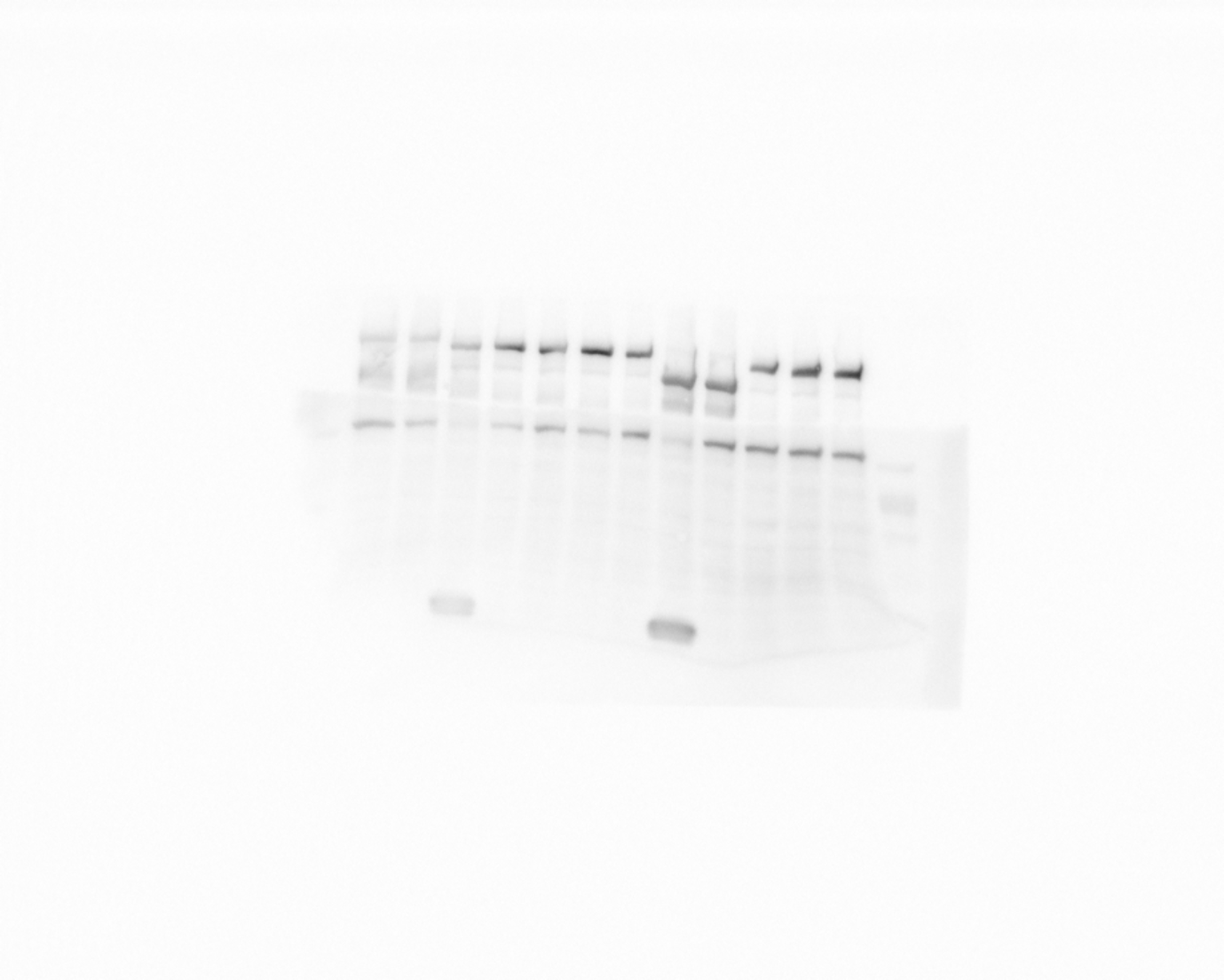

Supplement: Figure 4—source data 2. [file elife-105977-fig4-data2.zip › Figure 4 - Source data 2/Figure4B-source data 2/A-INPUT-GFP-MARK2-GFP-Cam20210202_115648_opt_6.TIF]

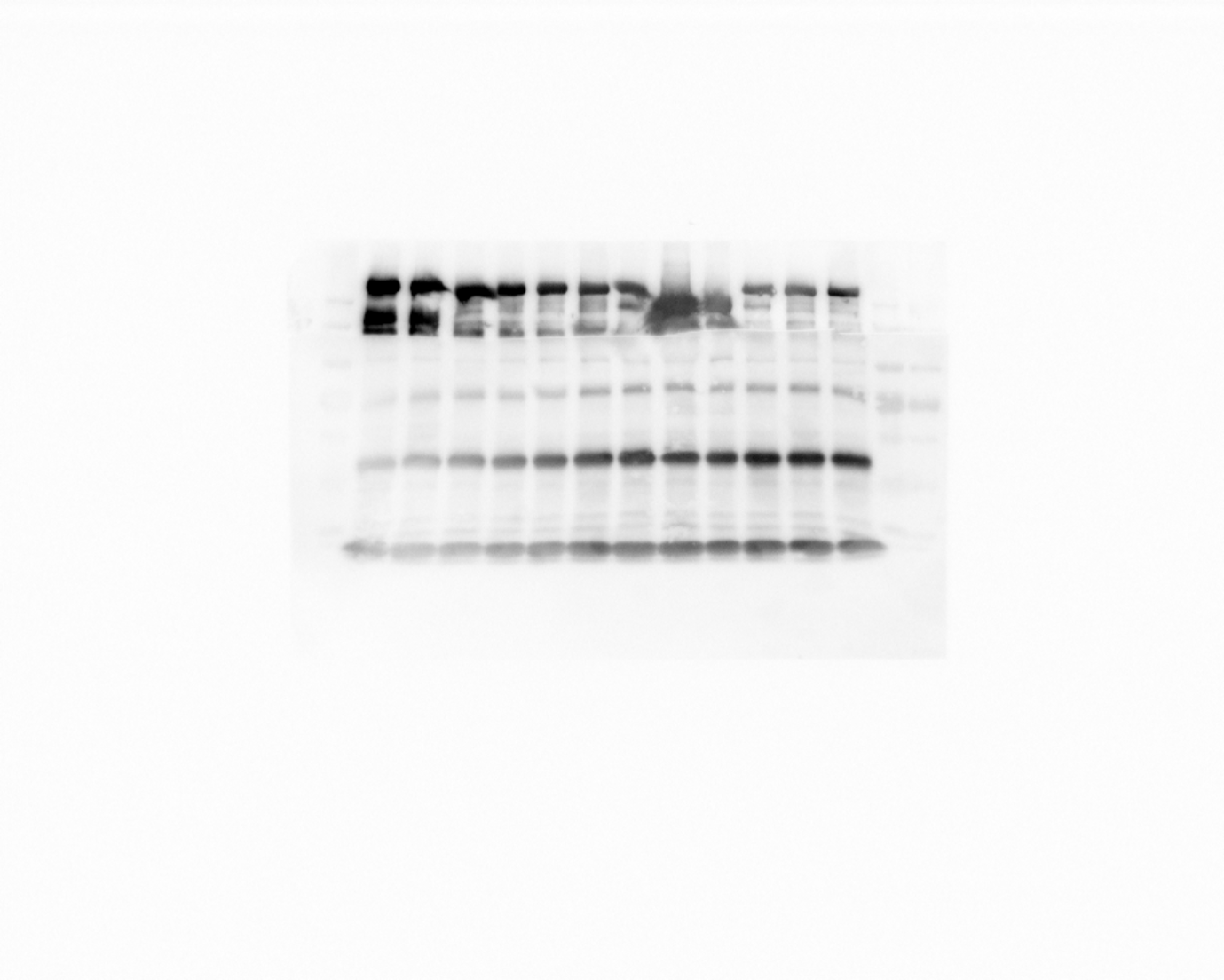

Supplement: Figure 4—source data 2. [file elife-105977-fig4-data2.zip › Figure 4 - Source data 2/Figure4B-source data 2/A-IP-FLAG-CAMSAP2-Cam20210202_115031_opt_6.TIF]

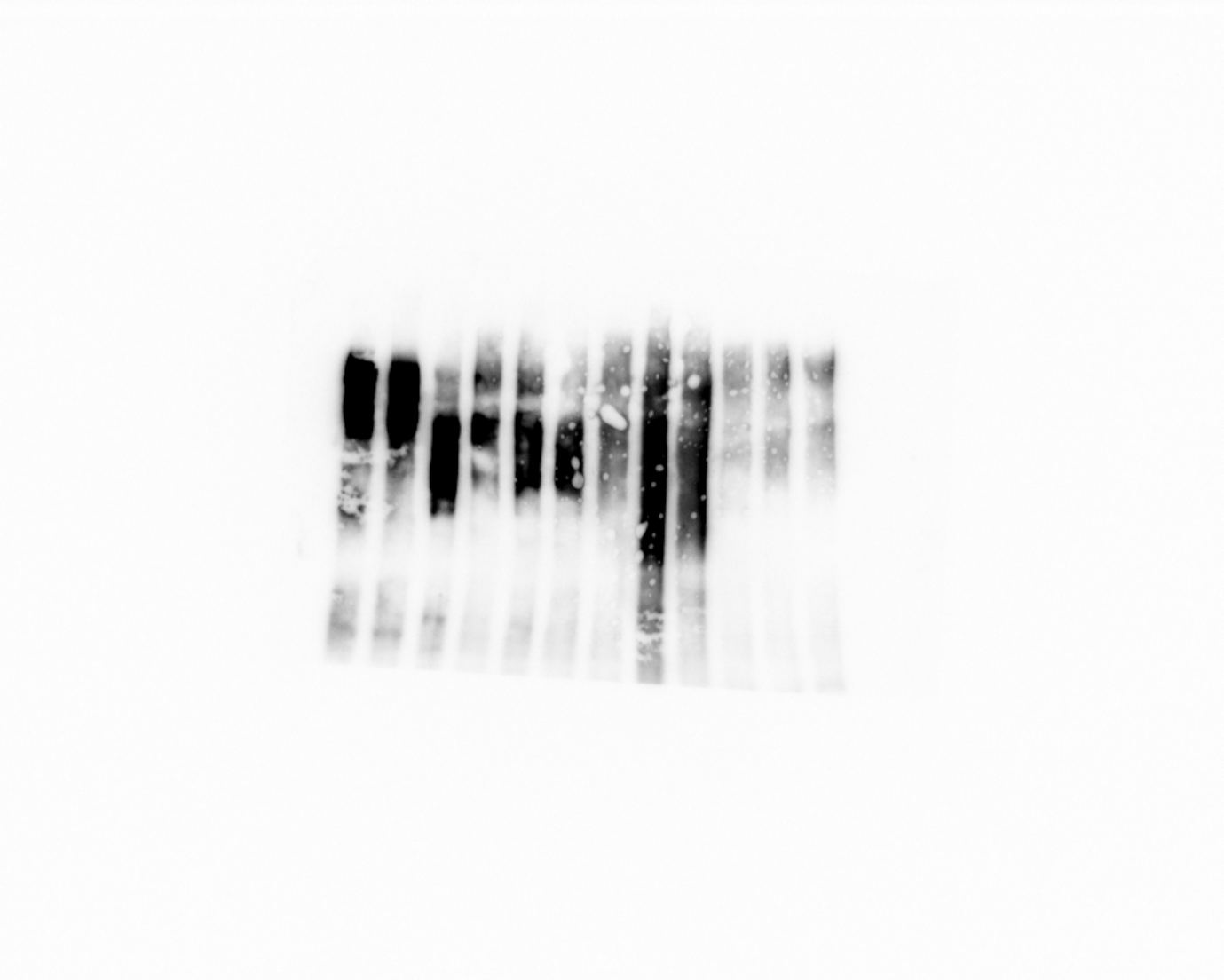

Supplement: Figure 4—source data 2. [file elife-105977-fig4-data2.zip › Figure 4 - Source data 2/Figure4B-source data 2/A-Phostag-Cam20210202_114206_opt_5.TIF]

Figure5D

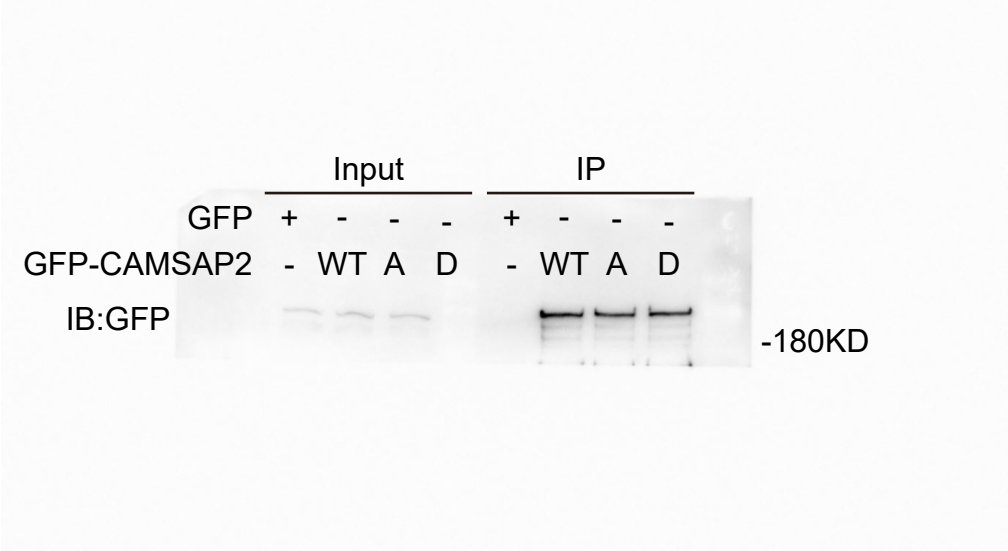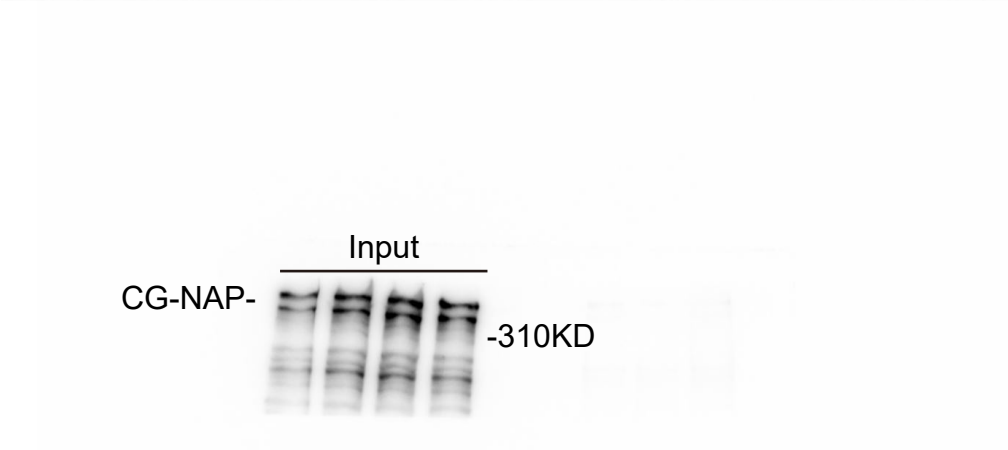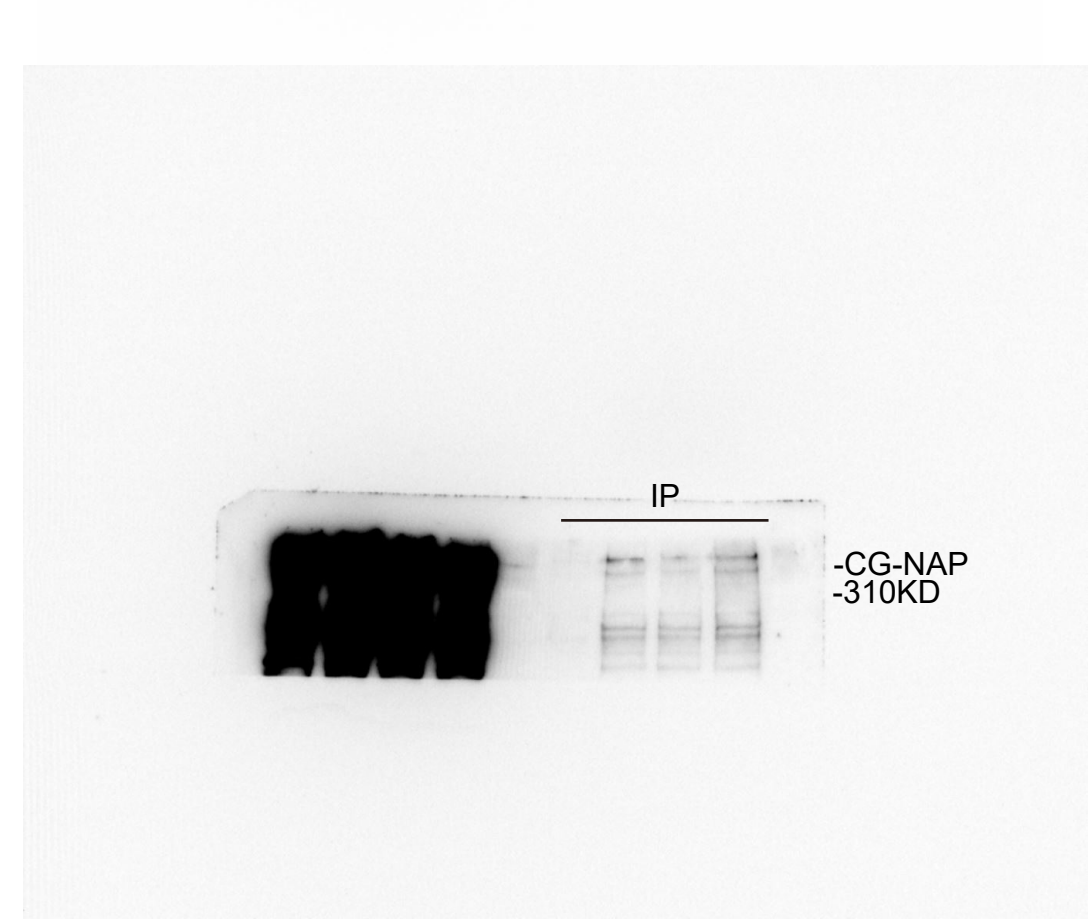

Supplement: Figure 5—source data 1. [file elife-105977-fig5-data1.zip › Figure 5 - Source data 1/Figure5D-source data 1-PDF/Figure5D-source data 1-PDF.pdf]

Figure 5F

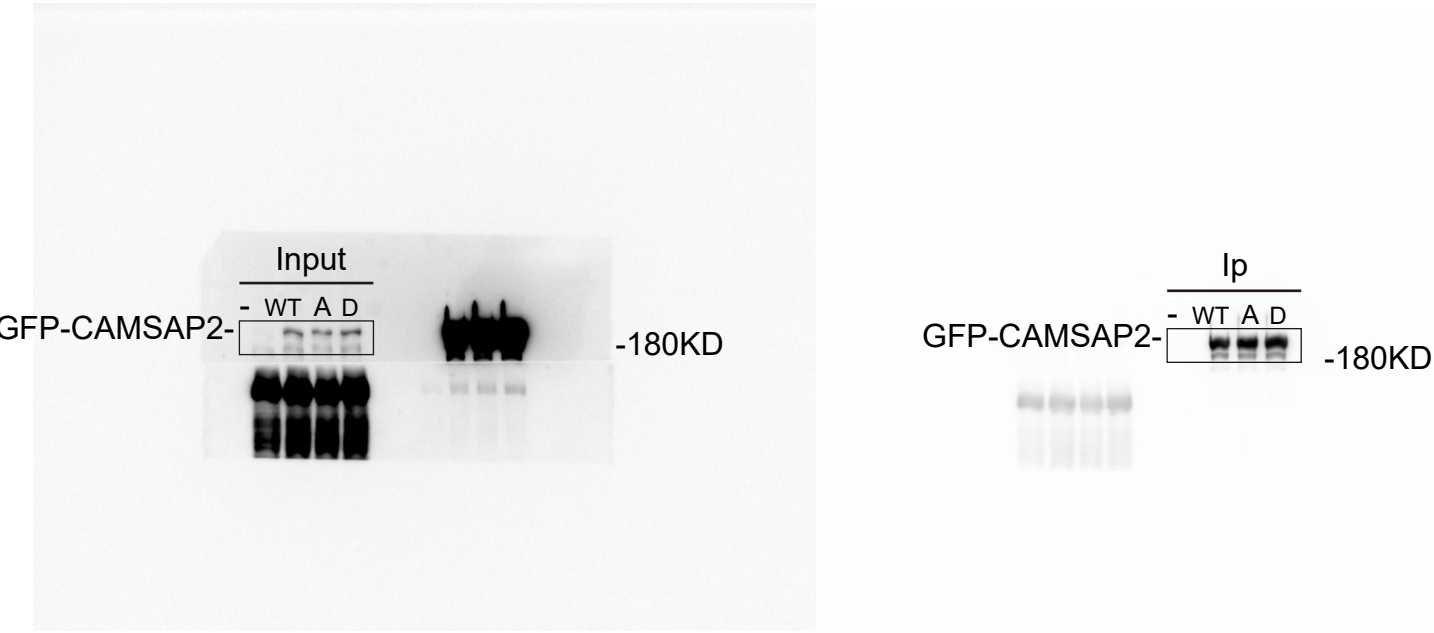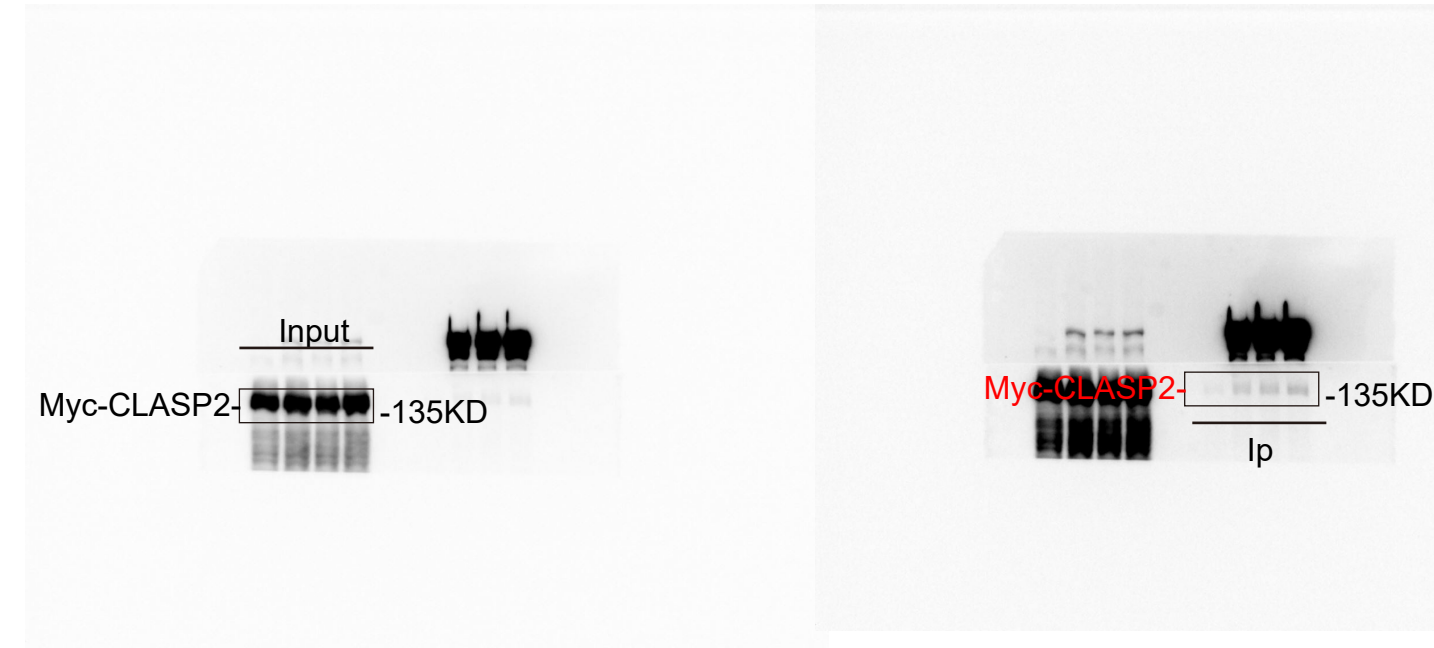

Supplement: Figure 5—source data 1. [file elife-105977-fig5-data1.zip › Figure 5 - Source data 1/Figure5F-source data 1-PDF/Figure5F-source data 1-PDF.pdf]

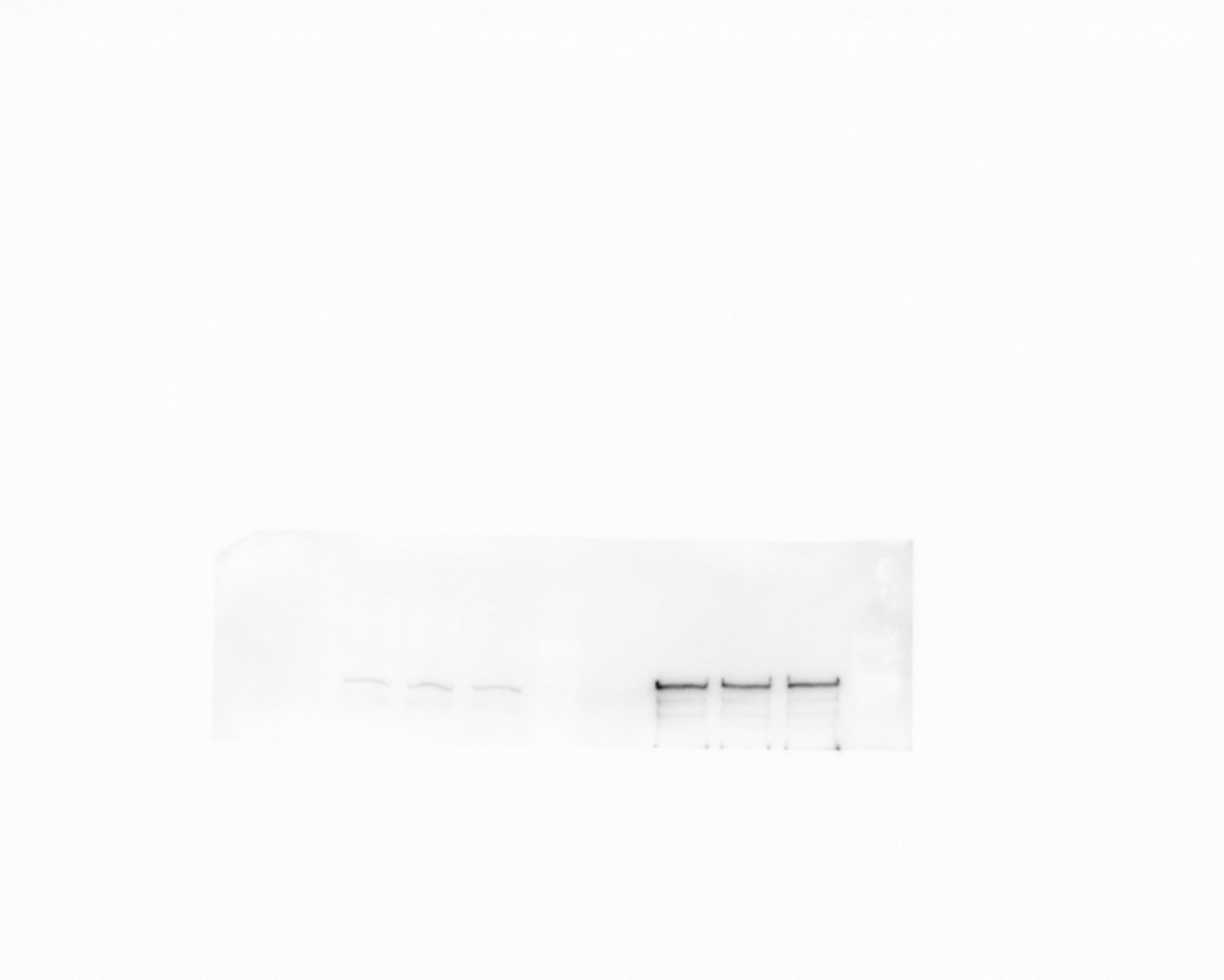

Supplement: Figure 5—source data 2. [file elife-105977-fig5-data2.zip › Figure 5 - Source data 2/Figure5D-source data 2/A-GFP-CAMSAP2-INPUT-IP-Cam20230720_200803_opt_5.TIF]

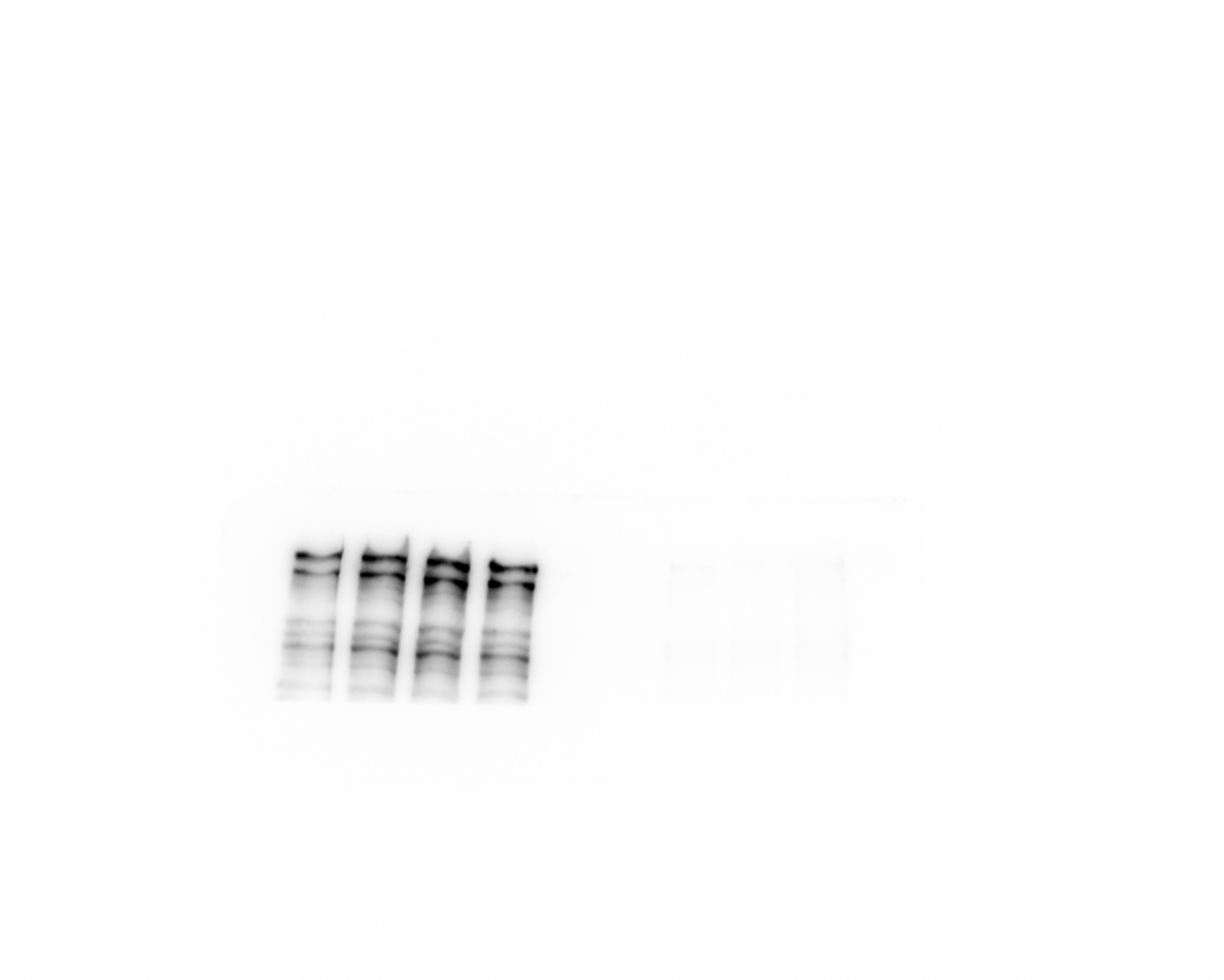

Supplement: Figure 5—source data 2. [file elife-105977-fig5-data2.zip › Figure 5 - Source data 2/Figure5D-source data 2/A-cgnap-Cam20230720_143947_opt_1.TIF]

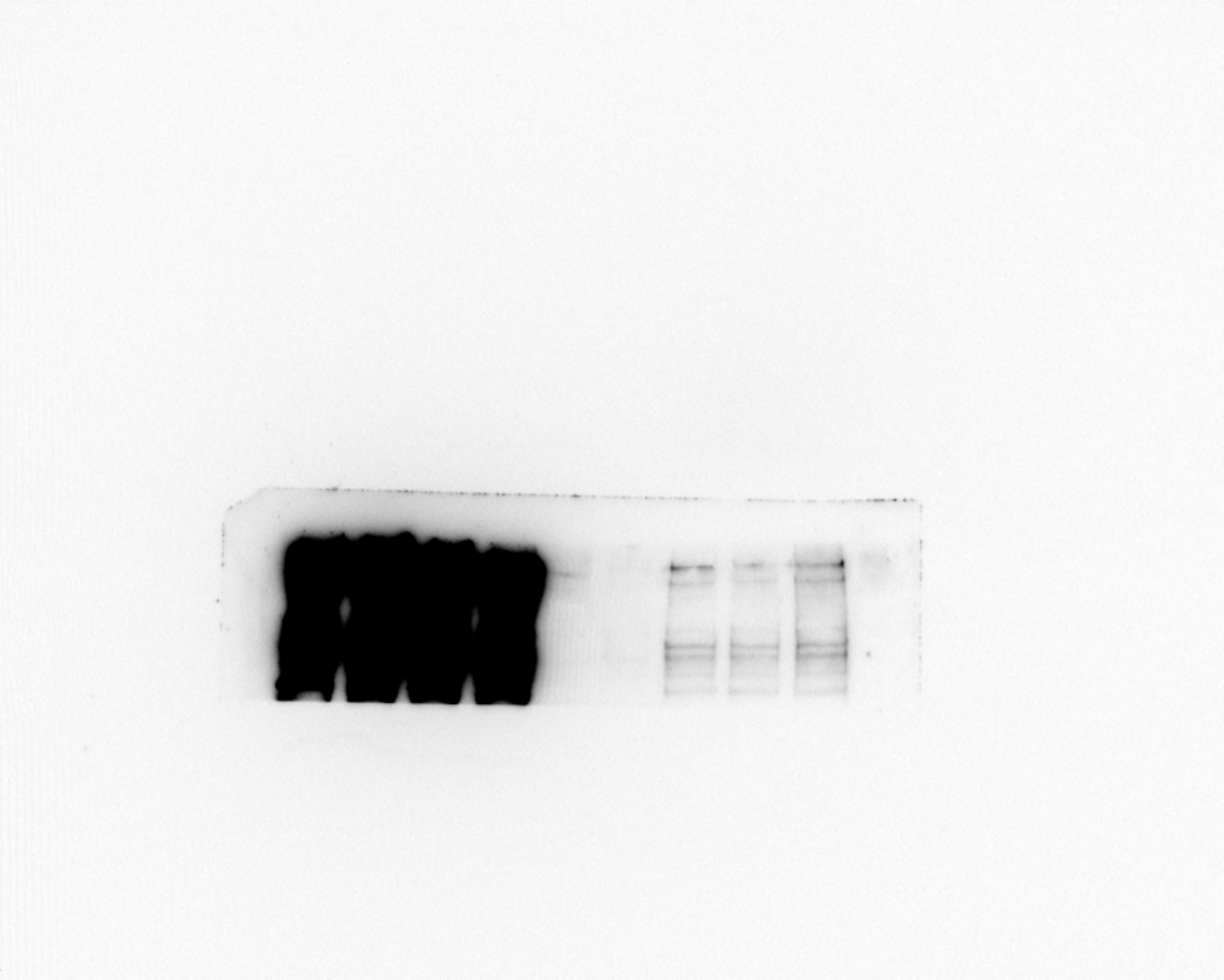

Supplement: Figure 5—source data 2. [file elife-105977-fig5-data2.zip › Figure 5 - Source data 2/Figure5D-source data 2/A-cgnap-Cam20230720_143947_opt_17.TIF]

Supplementary figure5A

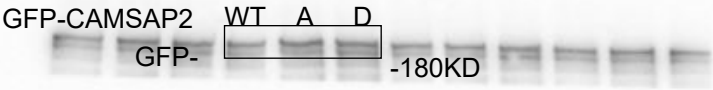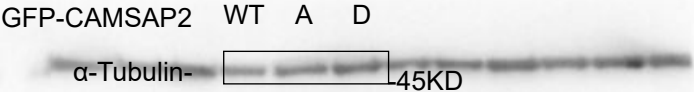

Supplement: Figure 5—figure supplement 1—source data 1. [file elife-105977-fig5-figsupp1-data1.zip › Figure 5-figure supplement 1 source data 1/Figure 5-figure supplement 1A source data 1.pdf]

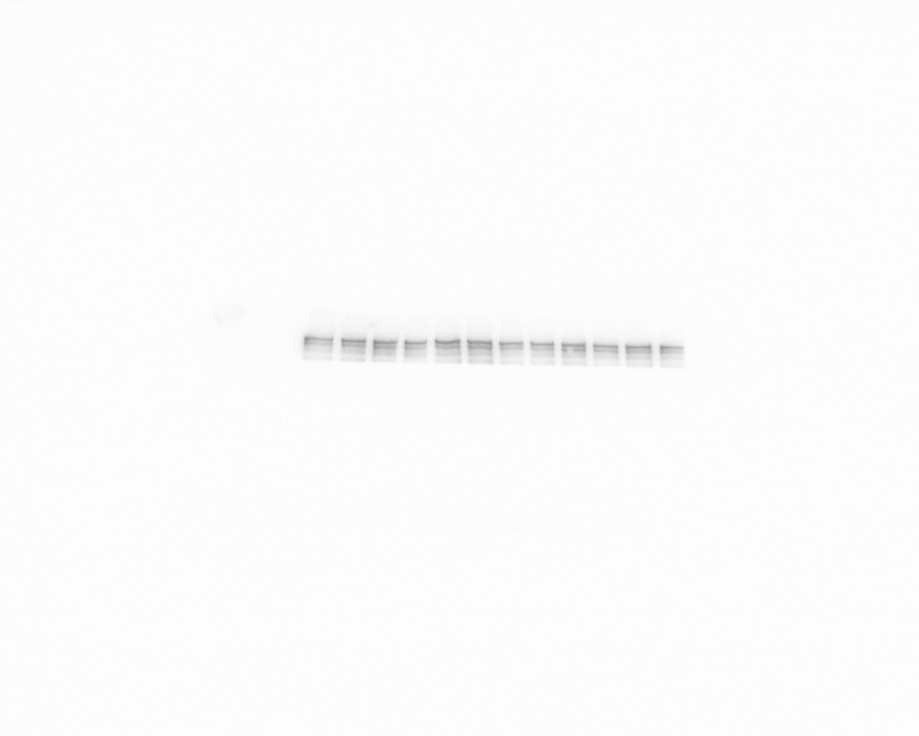

Supplement: Figure 5—figure supplement 1—source data 2. [file elife-105977-fig5-figsupp1-data2.zip › Figure 5-figure supplement 1 source data 2/Figure 5-figure supplement 1A source data 2/A-Cam20240108_203843_opt_4.TIF]

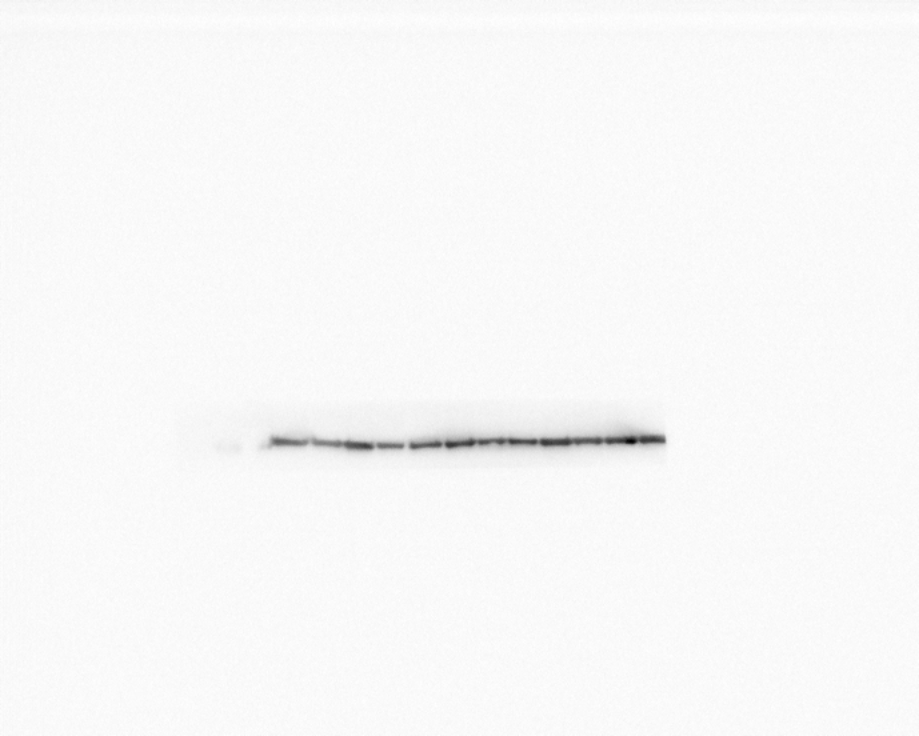

Supplement: Figure 5—figure supplement 1—source data 2. [file elife-105977-fig5-figsupp1-data2.zip › Figure 5-figure supplement 1 source data 2/Figure 5-figure supplement 1A source data 2/A-Cam20240108_204319_opt_13.TIF]

Figure6E

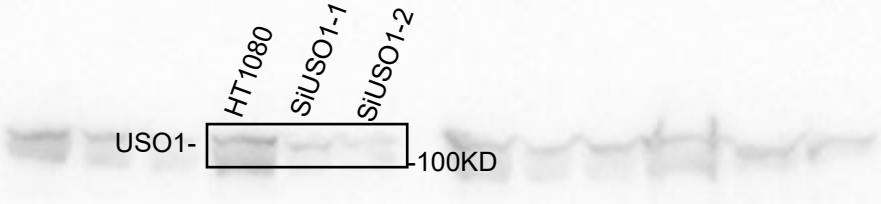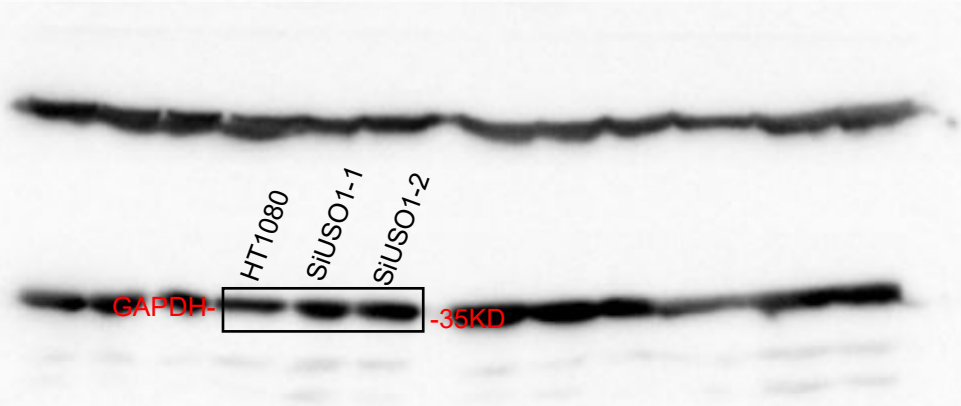

Supplement: Figure 6—source data 1. [file elife-105977-fig6-data1.zip › Figure 6 - Source data 1/Figure6E-source data 1-PDF/Figure6E-source data 1-PDF.pdf]

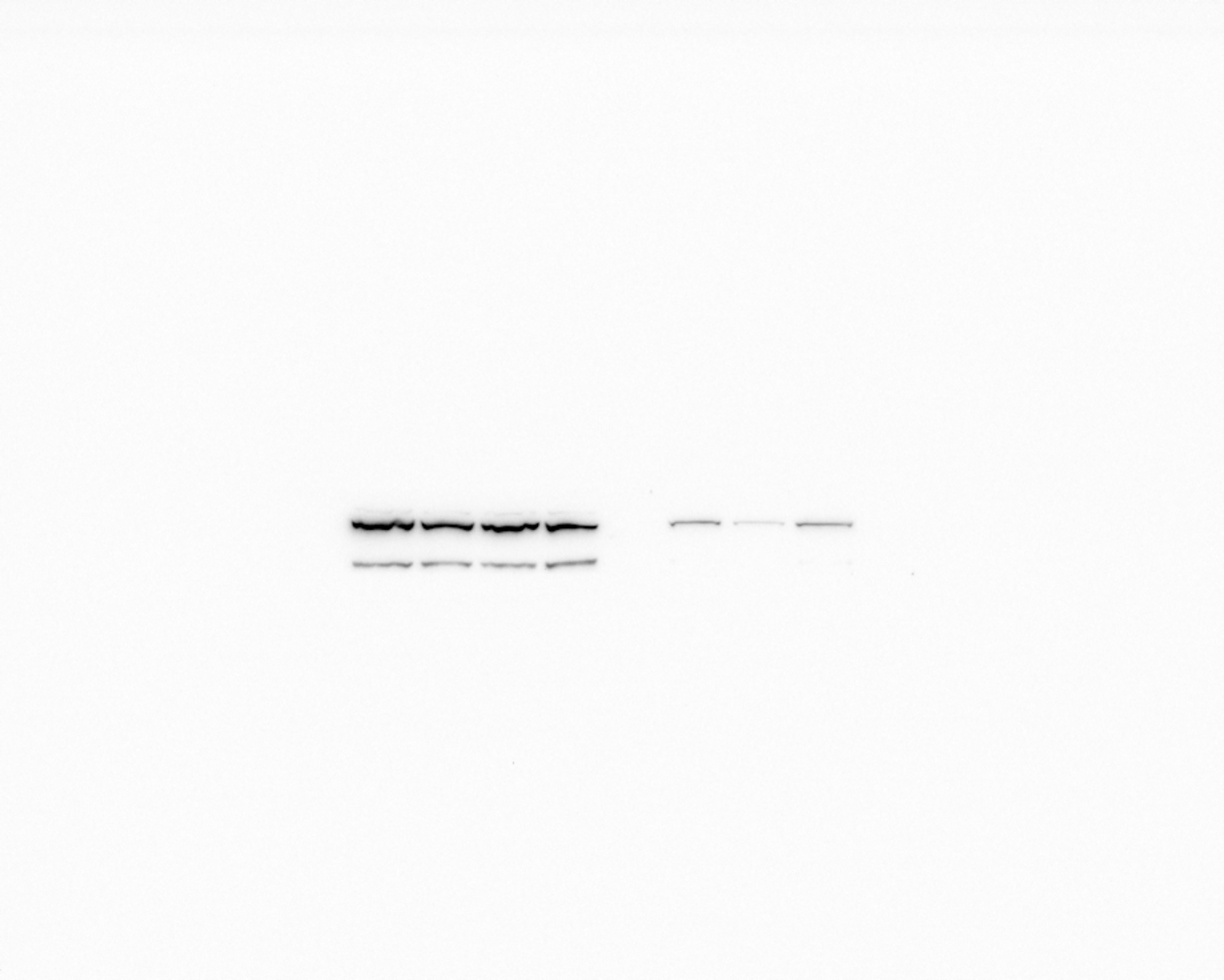

Supplement: Figure 6—source data 2. [file elife-105977-fig6-data2.zip › Figure 6 - Source data 2/Figure6C-source data 2/A-Cam20230707_105342_opt_9.TIF]

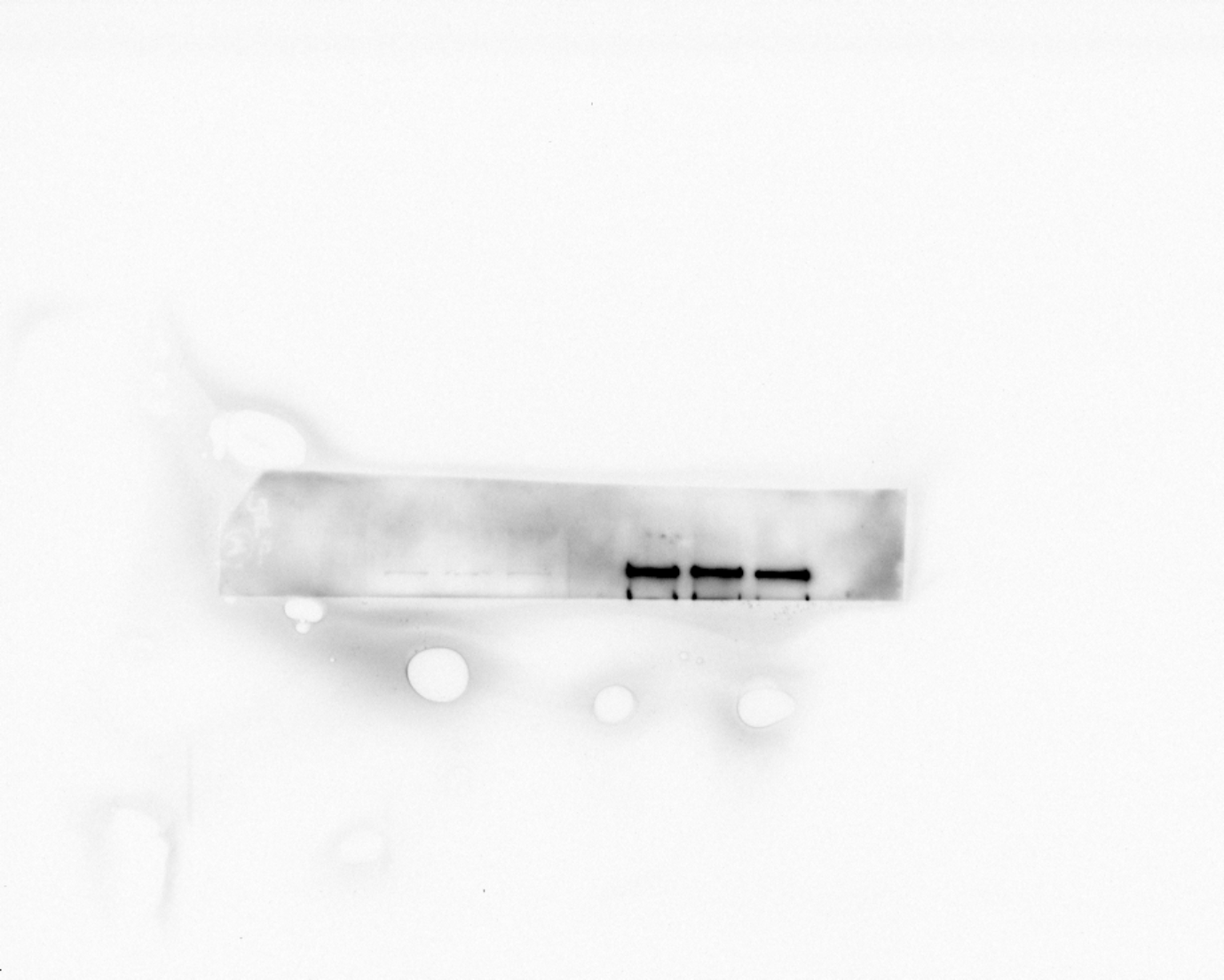

Supplement: Figure 6—source data 2. [file elife-105977-fig6-data2.zip › Figure 6 - Source data 2/Figure6C-source data 2/A-GFP-CAMSAP2AD-Cam20230707_105821_opt_14.TIF]

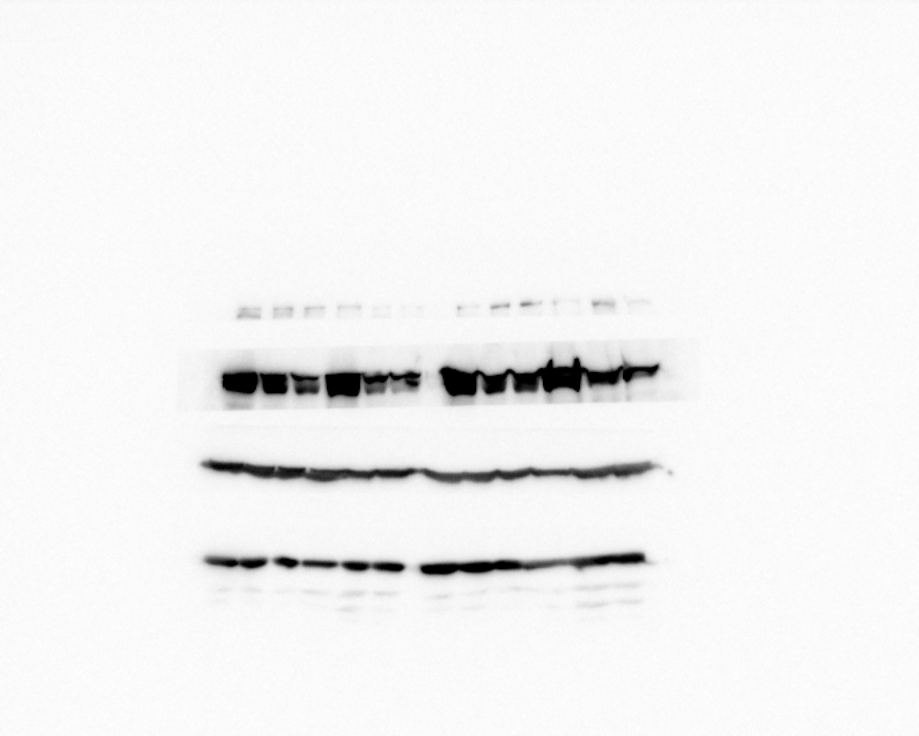

Supplement: Figure 6—source data 2. [file elife-105977-fig6-data2.zip › Figure 6 - Source data 2/Figure6E-source data 2/A-GAPDH-Cam20230726_213457_opt_11.TIF]

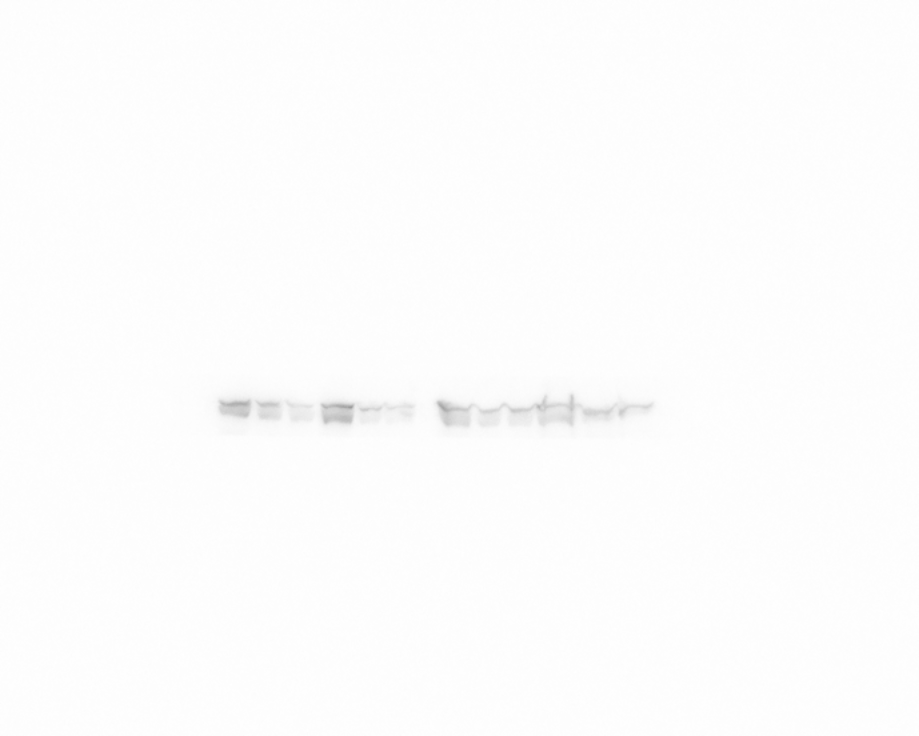

Supplement: Figure 6—source data 2. [file elife-105977-fig6-data2.zip › Figure 6 - Source data 2/Figure6E-source data 2/A-USO1-Cam20230726_213055_opt_1.TIF]

|                  |   |   |   |
|------------------|---|---|---|
| TurboID          | + | - | - |
| TurboID-CAMSAP2A | - | + | - |
| TurboID-CAMSAP2D | - | - | + |

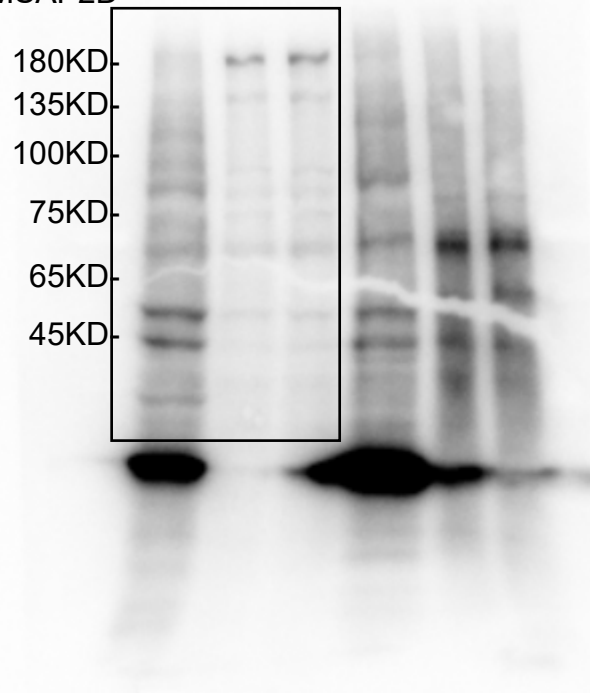

Supplement: Figure 6—figure supplement 1—source data 1. [file elife-105977-fig6-figsupp1-data1.zip › Figure 6-figure supplement 1 source data 1/Figure 6-figure supplement 1A source data .pdf]

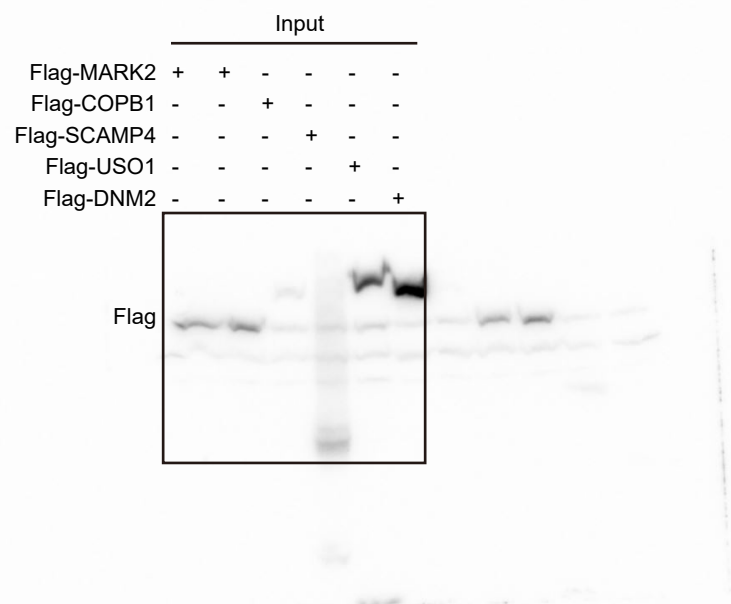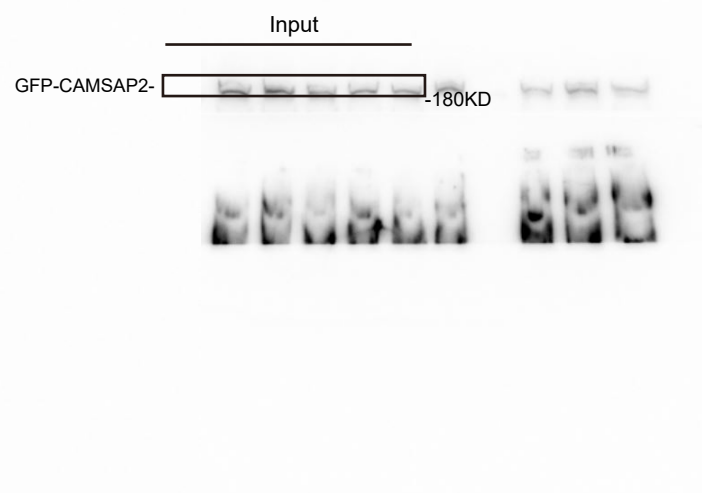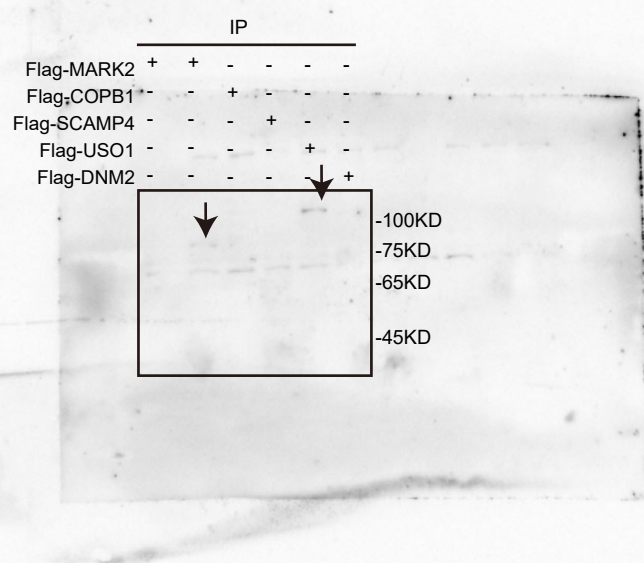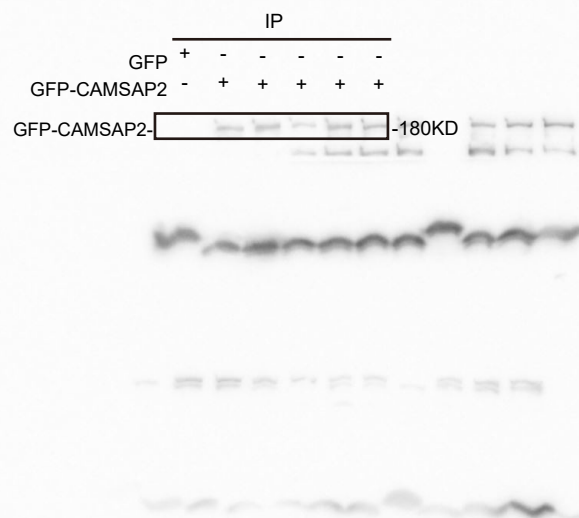

Supplement: Figure 6—figure supplement 1—source data 1. [file elife-105977-fig6-figsupp1-data1.zip › Figure 6-figure supplement 1 source data 1/Figure 6-figure supplement 1B source data .pdf]

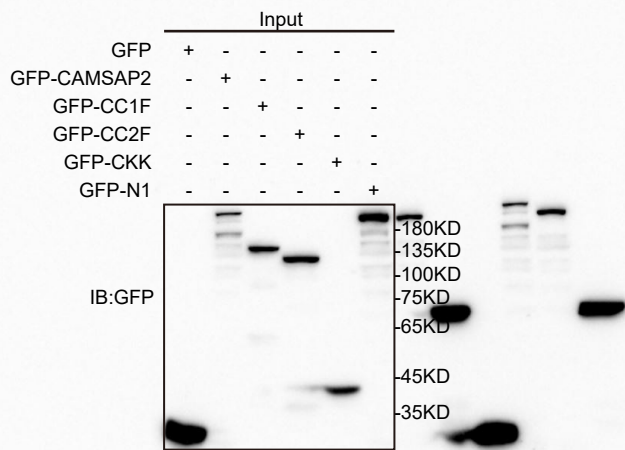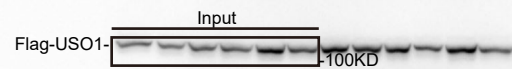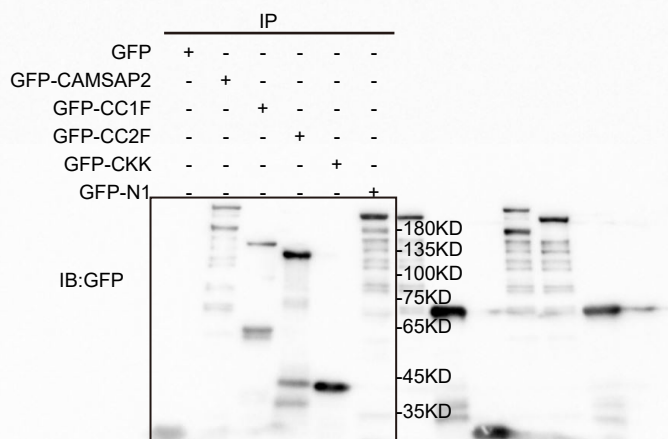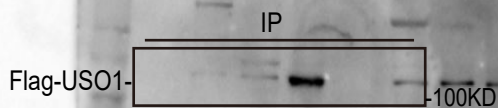

Supplement: Figure 6—figure supplement 1—source data 1. [file elife-105977-fig6-figsupp1-data1.zip › Figure 6-figure supplement 1 source data 1/Figure 6-figure supplement 1C source data .pdf]

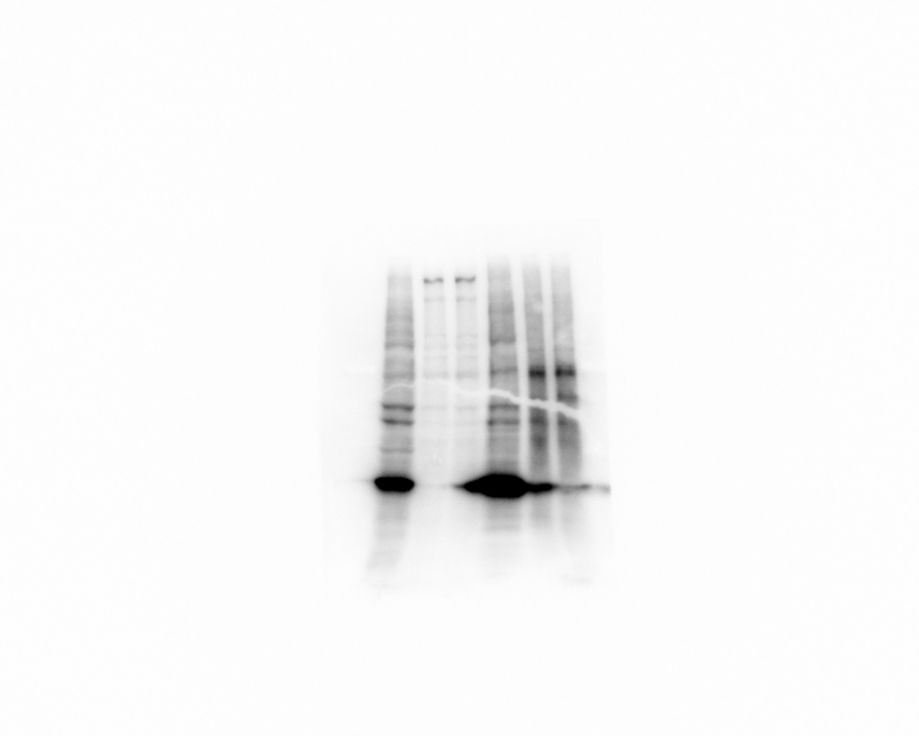

Supplement: Figure 6—figure supplement 1—source data 2. [file elife-105977-fig6-figsupp1-data2.zip › Figure 6-figure supplement 1 source data 2/Figure 6-figure supplement 1A source data 2/A-Cam20230831_220346_opt_2.TIF]

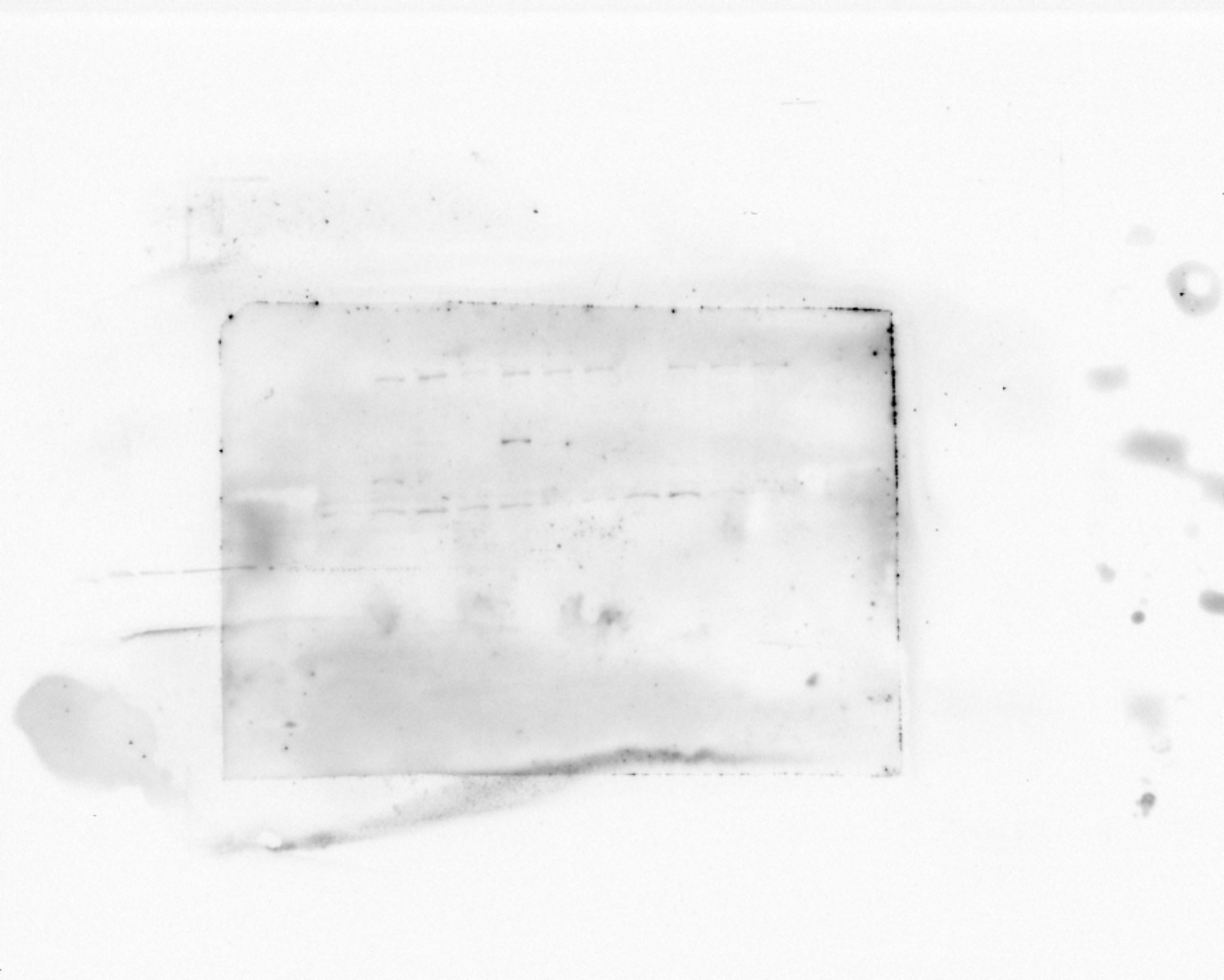

Supplement: Figure 6—figure supplement 1—source data 2. [file elife-105977-fig6-figsupp1-data2.zip › Figure 6-figure supplement 1 source data 2/Figure 6-figure supplement 1B source data 2/A-IP-Flag-Cam20230927_105259_opt_15.TIF]

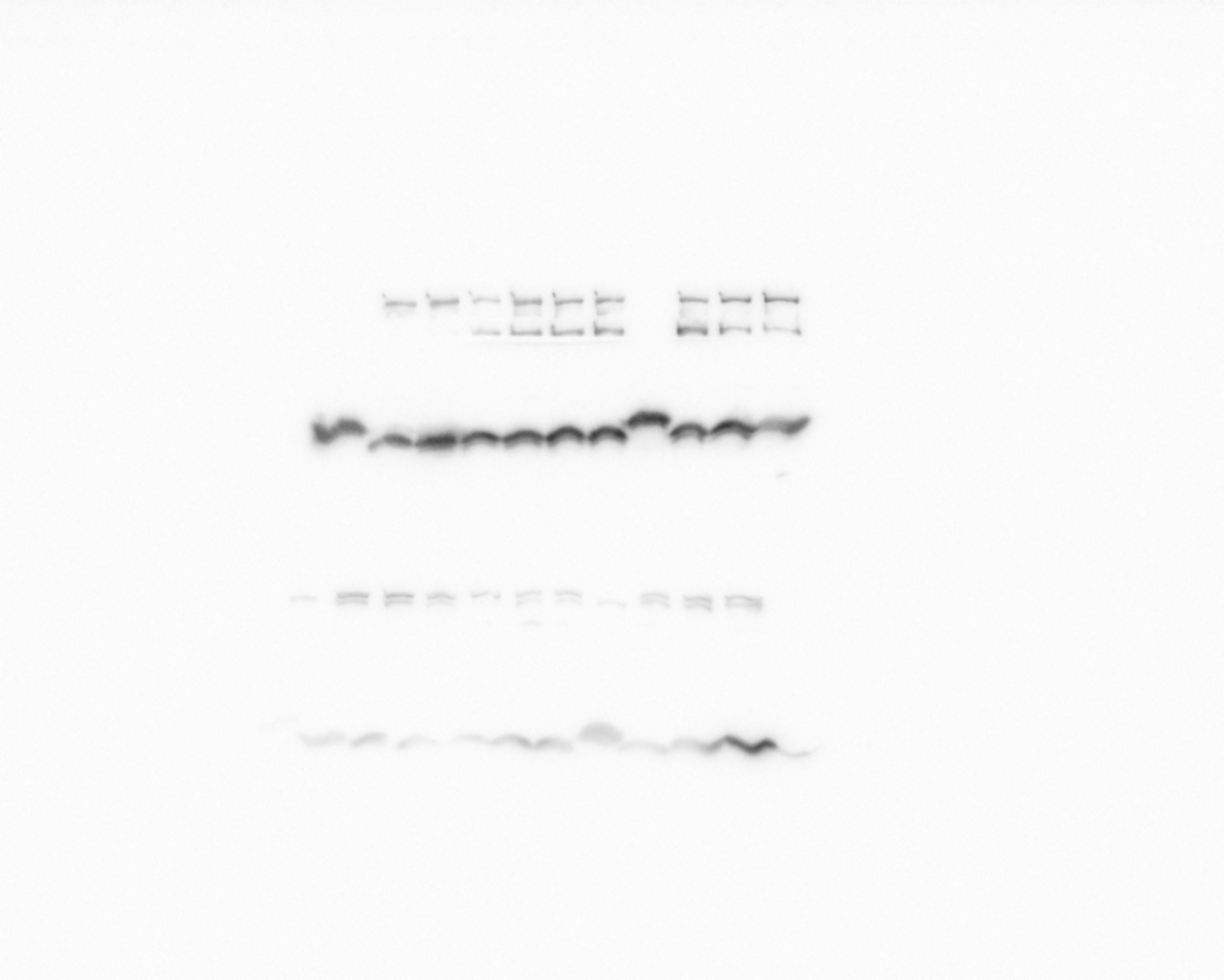

Supplement: Figure 6—figure supplement 1—source data 2. [file elife-105977-fig6-figsupp1-data2.zip › Figure 6-figure supplement 1 source data 2/Figure 6-figure supplement 1B source data 2/A-IP-GFP-CAMSAP2-Cam20230927_190641_opt_9.TIF]

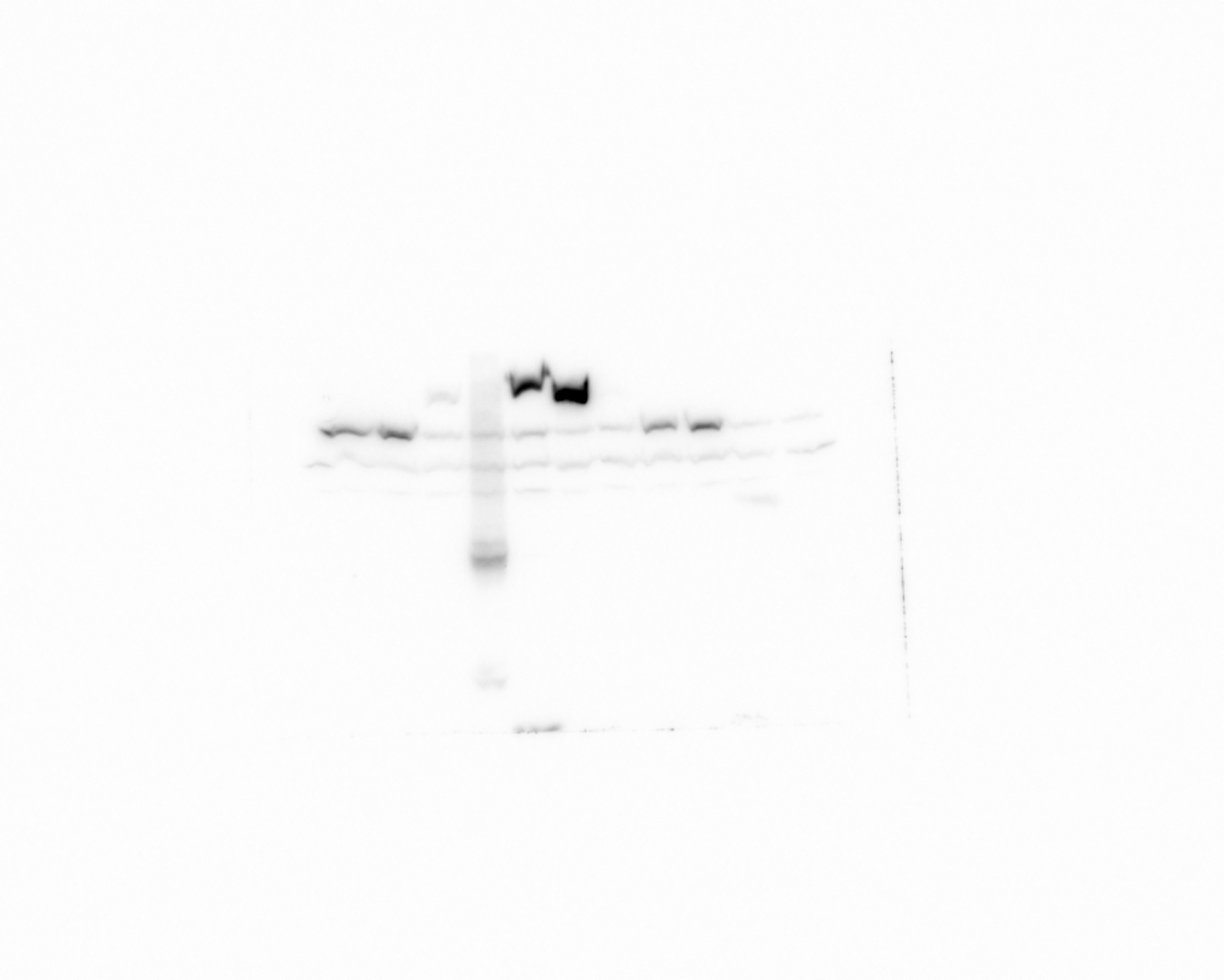

Supplement: Figure 6—figure supplement 1—source data 2. [file elife-105977-fig6-figsupp1-data2.zip › Figure 6-figure supplement 1 source data 2/Figure 6-figure supplement 1B source data 2/A-Input-Flag-Cam20230928_122615_opt_4.TIF]

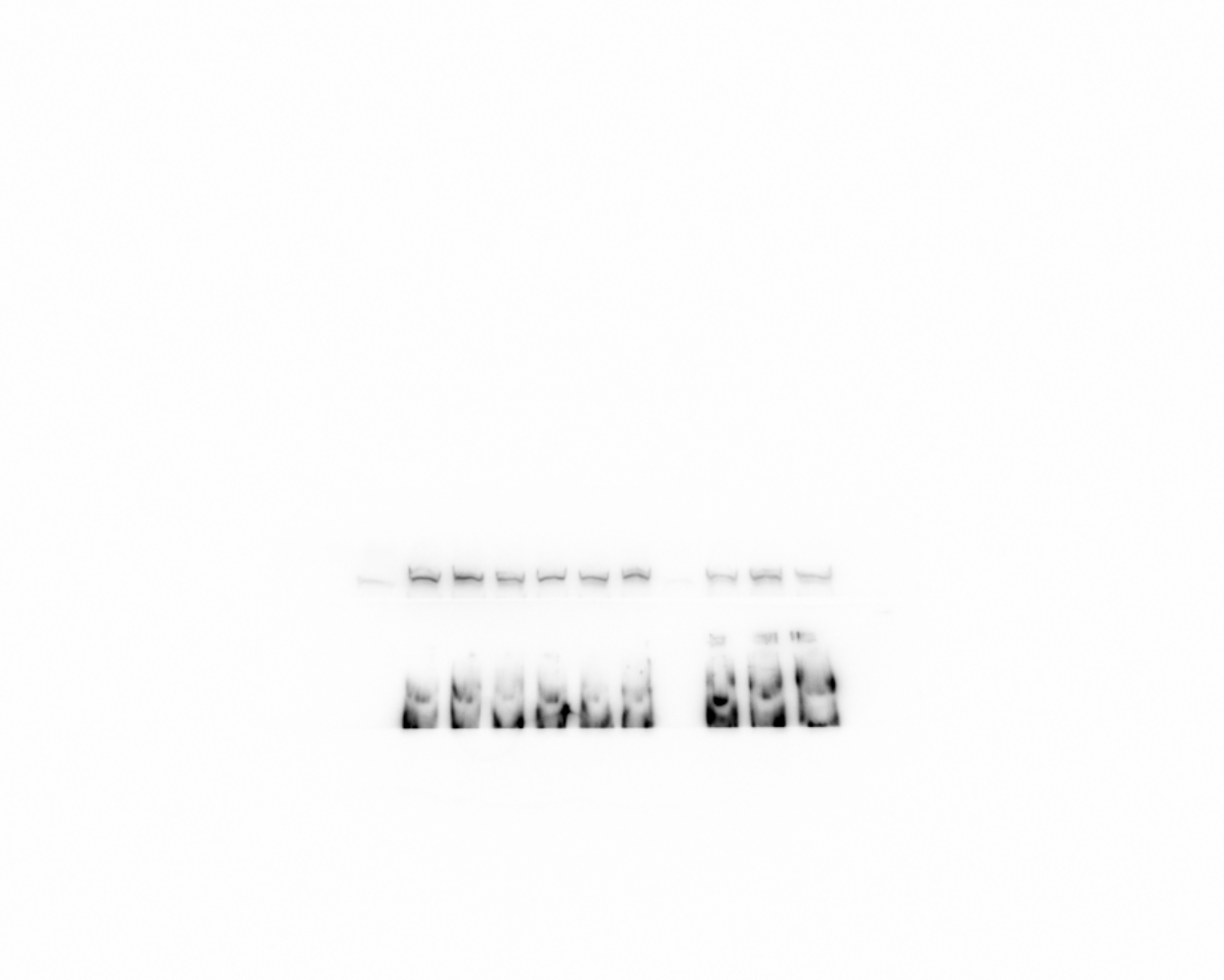

Supplement: Figure 6—figure supplement 1—source data 2. [file elife-105977-fig6-figsupp1-data2.zip › Figure 6-figure supplement 1 source data 2/Figure 6-figure supplement 1B source data 2/A-Input-gfp-camsap2-Cam20230928_123115_opt_2.TIF]

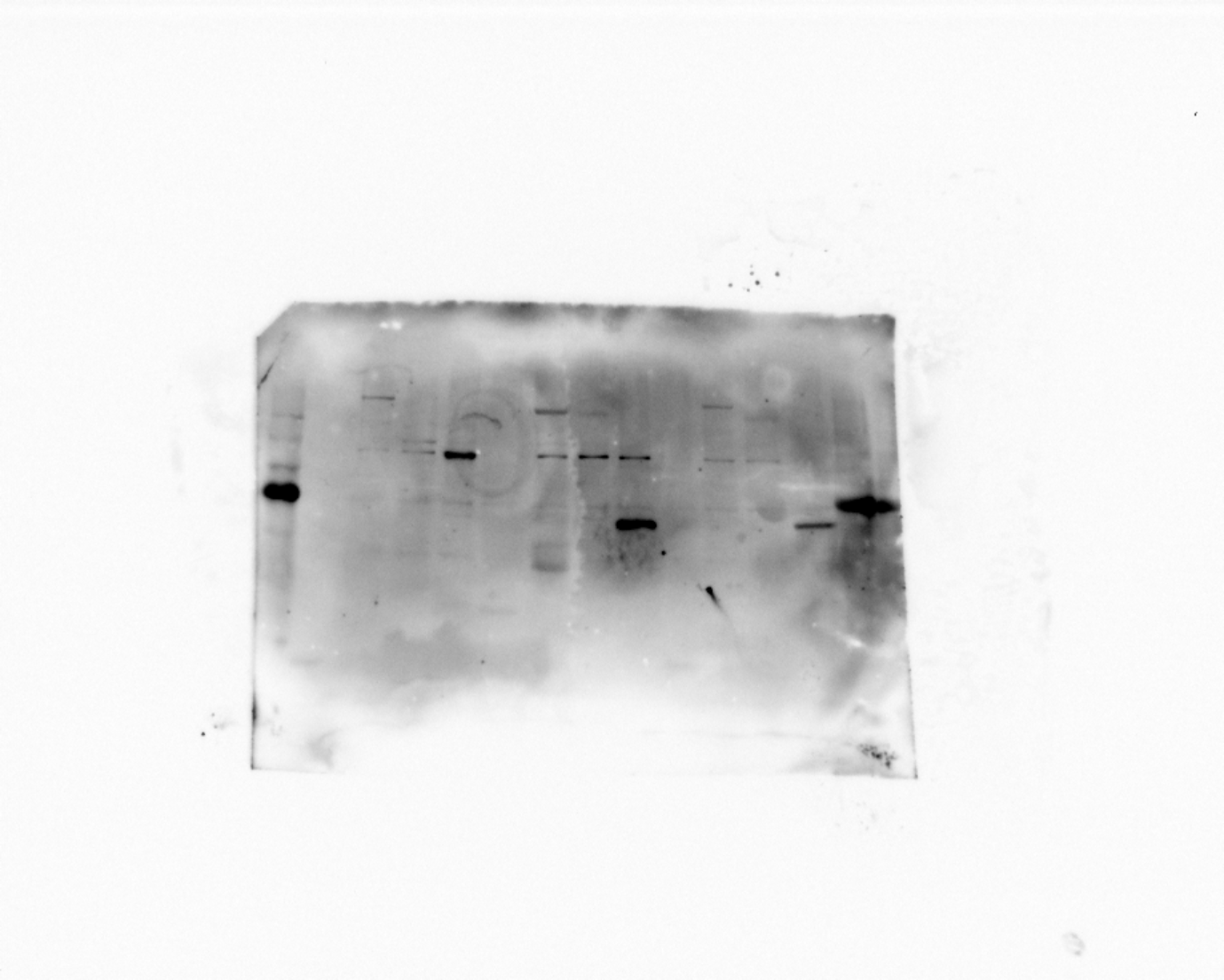

Supplement: Figure 6—figure supplement 1—source data 2. [file elife-105977-fig6-figsupp1-data2.zip › Figure 6-figure supplement 1 source data 2/Figure 6-figure supplement 1C source data 2/A-IP-FLAG-Cam20231218_163725_opt_11.TIF]

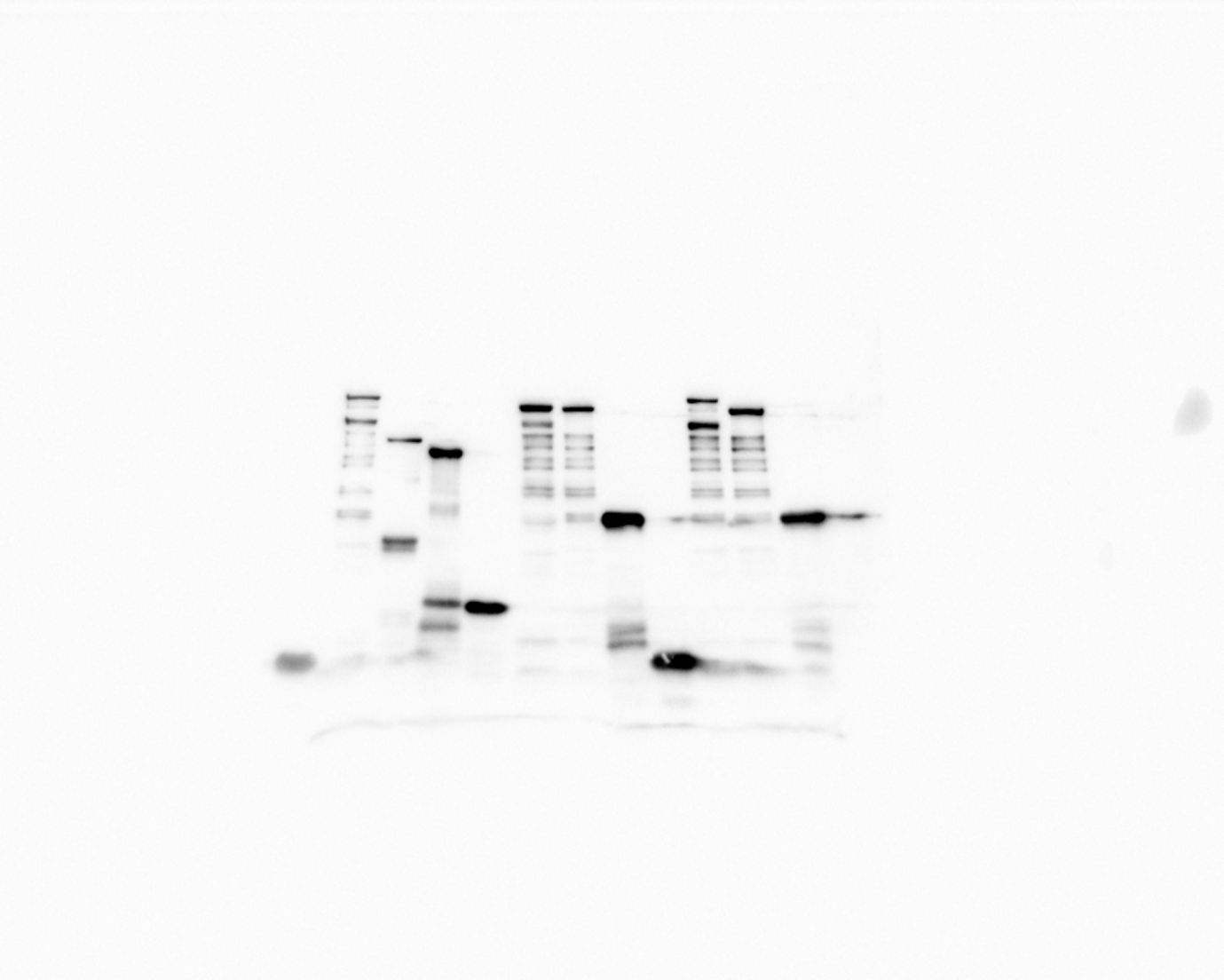

Supplement: Figure 6—figure supplement 1—source data 2. [file elife-105977-fig6-figsupp1-data2.zip › Figure 6-figure supplement 1 source data 2/Figure 6-figure supplement 1C source data 2/A-IP-GFP-Cam20231219_115241_opt_9.TIF]

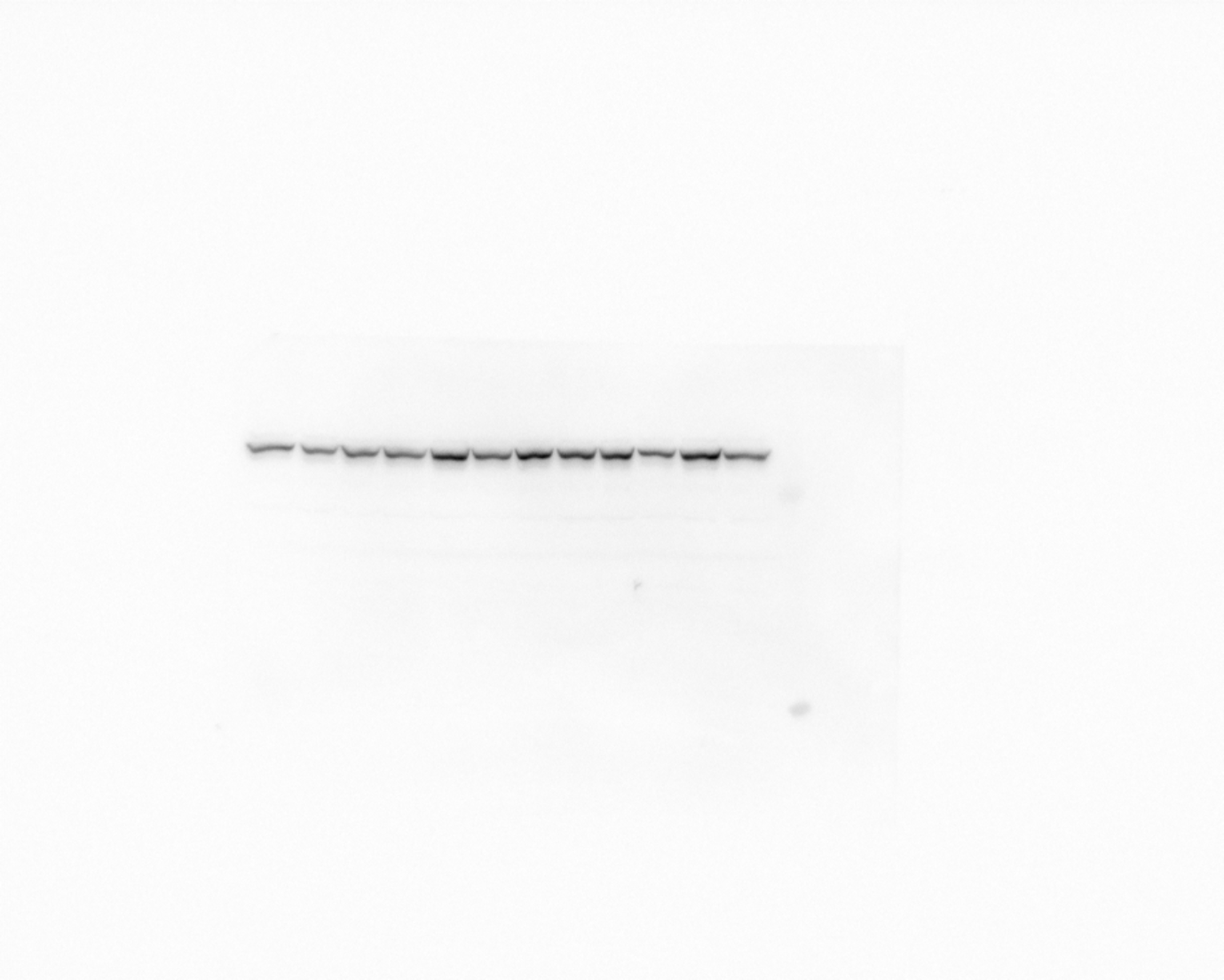

Supplement: Figure 6—figure supplement 1—source data 2. [file elife-105977-fig6-figsupp1-data2.zip › Figure 6-figure supplement 1 source data 2/Figure 6-figure supplement 1C source data 2/A-Input-Flag-Cam20231218_163214_opt_8.TIF]

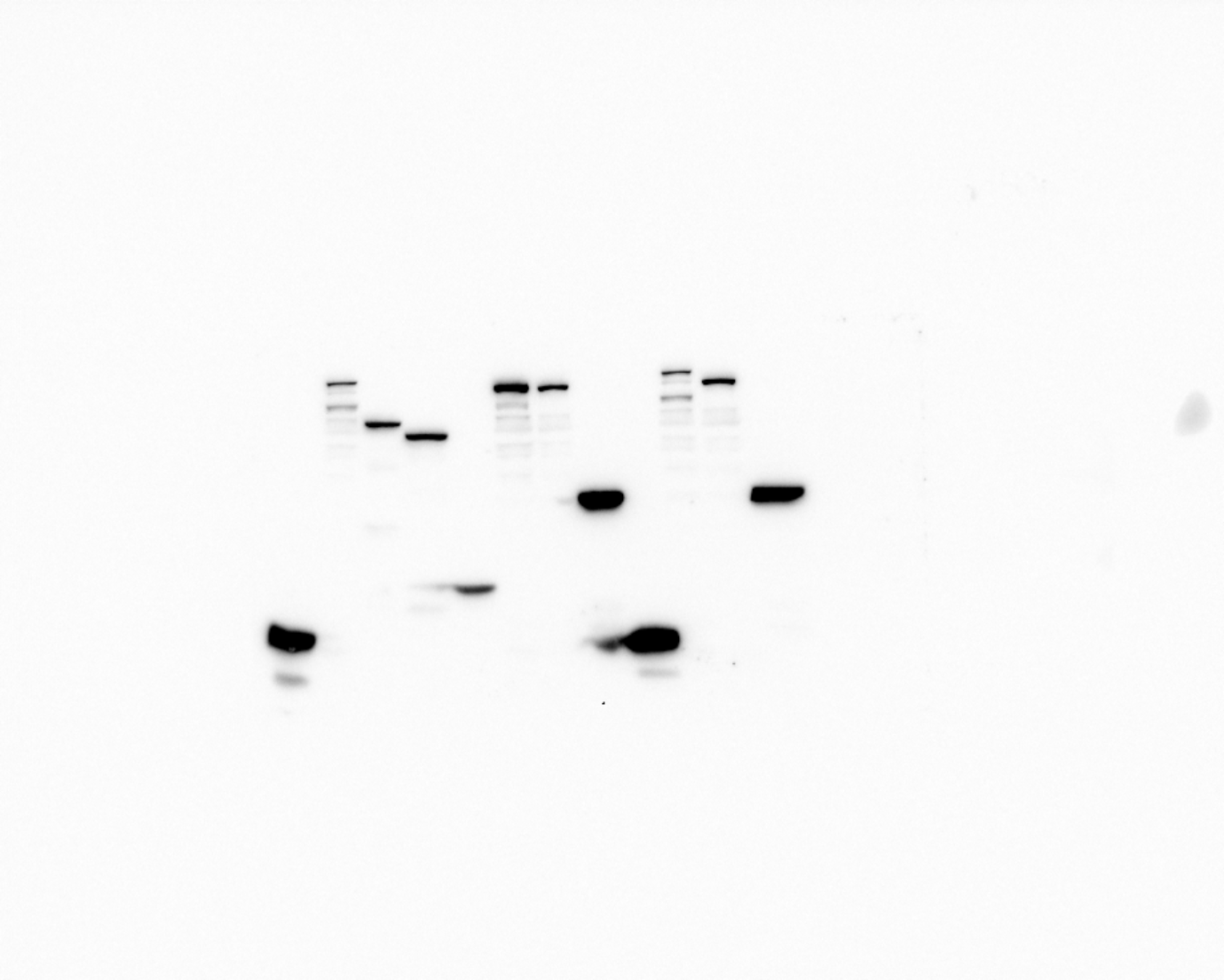

Supplement: Figure 6—figure supplement 1—source data 2. [file elife-105977-fig6-figsupp1-data2.zip › Figure 6-figure supplement 1 source data 2/Figure 6-figure supplement 1C source data 2/A-input-GFP-Cam20231219_114545_opt_9.TIF]
