# Supplementary material for: MARK2 regulates Golgi apparatus reorientation by phosphorylation of CAMSAP2 in directional cell migratio
Source: eLife. 2025 May 7;14:RP105977. doi: 10.7554/eLife.105977 (PMC12058119; doi:10.7554/eLife.105977)
Supplement: Figure 2—figure supplement 1—source data 1. [file elife-105977-fig2-figsupp1-data1.zip › Figure 2-figure supplement 1 source data 1/Figure 2-figure supplement 1B source data .pdf]

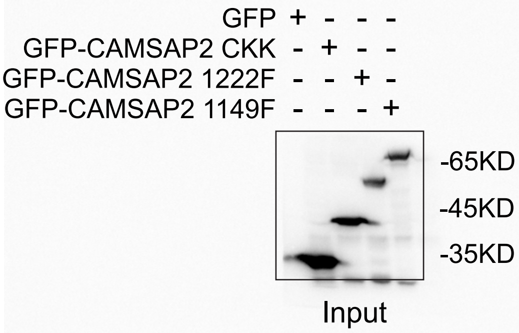

GFP + - - -
GFP-CAMSAP2 CKK - + - -
GFP-CAMSAP2 1222F - - + -
GFP-CAMSAP2 1149F - - - +
-65KD
-45KD
-35KD
Input

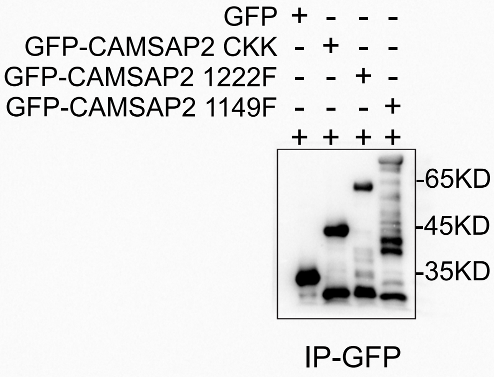

GFP + - - -
GFP-CAMSAP2 CKK - + - -
GFP-CAMSAP2 1222F - - + -
GFP-CAMSAP2 1149F - - - +
+ + + +
-65KD
-45KD
-35KD
IP-GFP

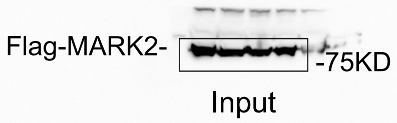

Flag-MARK2-
-75KD
Input

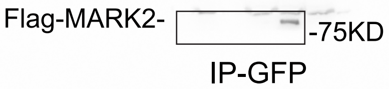

Flag-MARK2-
-75KD
IP-GFP
