# Supplementary material for: MARK2 regulates Golgi apparatus reorientation by phosphorylation of CAMSAP2 in directional cell migratio
Source: eLife. 2025 May 7;14:RP105977. doi: 10.7554/eLife.105977 (PMC12058119; doi:10.7554/eLife.105977)
Supplement: Figure 6—source data 1. [file elife-105977-fig6-data1.zip › Figure 6 - Source data 1/Figure6C-source data 1-PDF/Figure6C-source data 1-PDF.pdf]

Figure6C

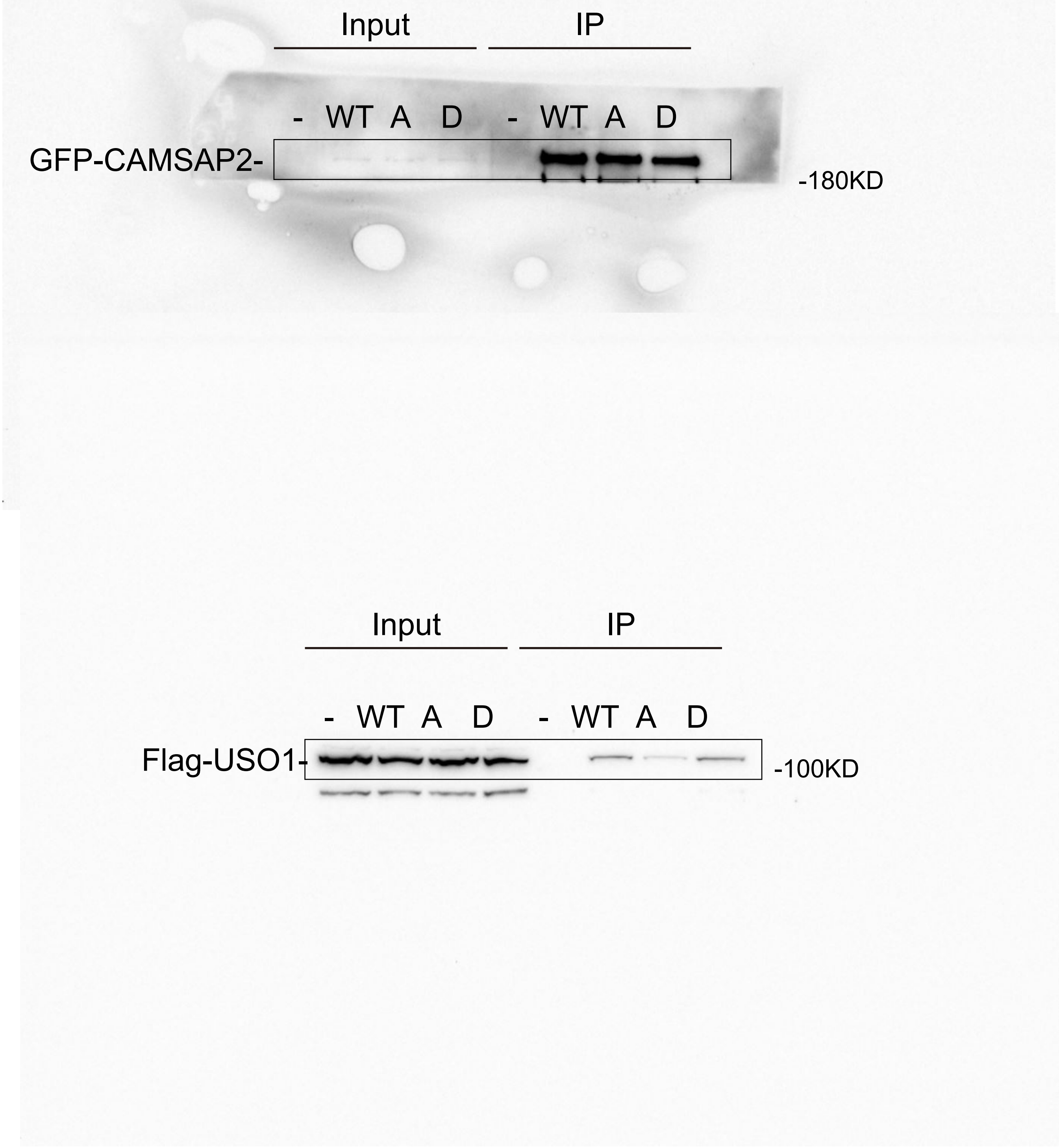

Input
IP
- WT A D - WT A D
GFP-CAMSAP2-
-180KD
Input
IP
- WT A D - WT A D
Flag-USO1-
-100KD
